# Supplementary material for: Could perturbed fetal development of the ovary contribute to the development of polycystic ovary syndrome in later life?
Source: PLoS One. 2020 Feb 20;15(2):e0229351. doi: 10.1371/journal.pone.0229351 (PMC7032716; doi:10.1371/journal.pone.0229351)

Fig S1. Representative images of bovine fetal ovary showing the cortical and medullar areas prior to and post LCM. The whole ovary was embedded into cryomold filled with OCT compound with the hilum on the side of the mold. For confirming the cortical and medullar areas, 5-20 section of 8 µm thickness were cut and mounted into colourfrost glass slides (HD Scientific Supplies). Slides were then fixed and stained in 1% cresyl violet acetate (pH 7.75) in 70% ethanol using the same protocol as described in this study. The cortical area was identified as the outer area lying beneath the tunica albugines, composed of connective tissue and fibers, as well as scattered ovigerous cords and/or primordial/primary follicles. The medullar area was identified as the middle area, composed of connective tissue and fibers with cvarious szes of vasculature. After those areas were identified using microscope Olympus BX-50, then 10-12 sections of 8 µm thickness were cut and transferred into the PET membrane frame slides. Scale bars: 50 µm. LCM, laser capture microdissection. OCT, optimal cutting temperature.

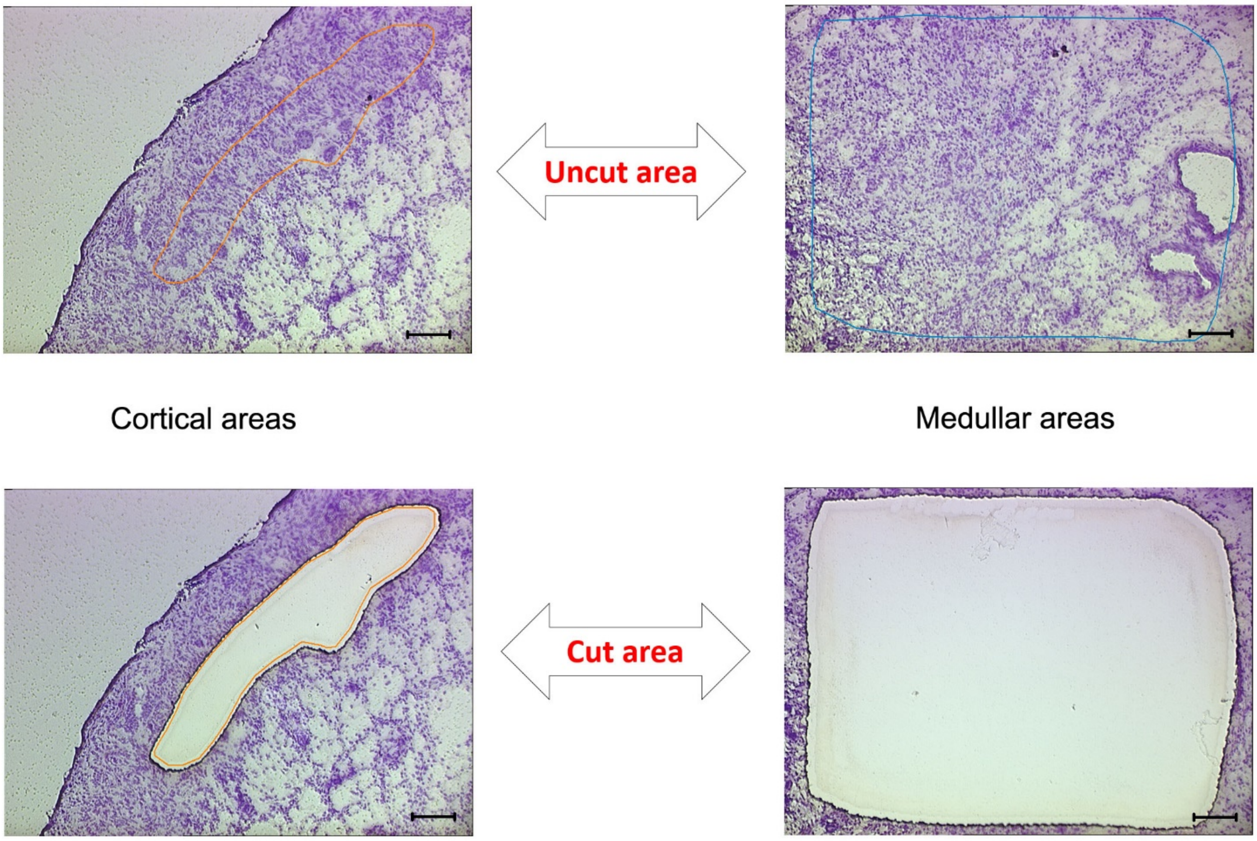

Supplement: S1 Fig — (PDF) [file pone.0229351.s001.pdf]
