# Supplementary material for: Could perturbed fetal development of the ovary contribute to the development of polycystic ovary syndrome in later life?
Source: PLoS One. 2020 Feb 20;15(2):e0229351. doi: 10.1371/journal.pone.0229351 (PMC7032716; doi:10.1371/journal.pone.0229351)
Supplement: S2 Fig — (PDF) [file pone.0229351.s002.pdf]

Fig S2. **Alignment of the exons of human *DENND1A.V1*; bovine *DENND1A*; predicted bovine *DENND1A.X1,2,3,4*; and *DENND1A.X1,2,3,4* primer sequences.** Alignment was generated with CLUSTAL O (1.2.4) multiple sequence alignment based on sequences available at Ensembl Asia with transcript ID: human *DENND1A.V1* (ENST00000373624.6) and bovine *DENND1A* (ENSBTAG00000003610) and at NCBI PubMed with accession numbers: predicted bovine *DENND1A.X1* (XM_005213126.4); *DENND1A.X2* (XM_015473579.2); *DENND1A.X3* (XM_015473580.2); and *DENND1A.X4* (XM_024998568.1). The alignment was analysed with T-COFFEE (1) and colored based on the consistency: red (high), yellow (average); green and blue (poor). Consistency is estimated from CORE index. Bright aqua highlighted areas represent the primer sequences. .... represent intronic areas not shown so as to reduce the size of this file.

1. Notredame C, Higgins DG, Heringa J. T-Coffee: A novel method for fast and accurate multiple sequence alignment. J Mol Biol. 2000;302(1):205-17. doi: 10.1006/jmbi.2000.4042.

```
DENND1A_BOVINE      ------------------------------------------------------------------------------------
DENND1A.V1_HUMAN    CGCGCGCCGGGCACGCGCGCCGGCGACCATGGCGTTCGCCGGGCTGGAGCGAGTACATTAACCCCTGGAGGCGGCGGCGGC---
DENND1A.X1_BOVINE   -------------------CCGGCGACCATGGCGTTCGCCCGGCTGAAGCGAGTGCATTAACCCCGGGAGGCGGCGGCGGCGGC
DENND1A.X2_BOVINE   --------------------CGGCGACCATGGCGTTCGCCCGGCTGAAGCGAGTGCATTAACCCCGGGAGGCGGCGGCGGCGGC
DENND1A.X3_BOVINE   --------------------CGGCGACCATGGCGTTCGCCCGGCTGAAGCGAGTGCATTAACCCCGGGAGGCGGCGGCGGCGGC
DENND1A.X4_BOVINE   -----------------------CGACCATGGCGTTCGCCCGGCTGAAGCGAGTGCATTAACCCCGGGAGGCGGCGGCGGCGGC
DENND1A.X1-4_F      ------------------------------------------------------------------------------------
DENND1A.X1-4_R      ------------------------------------------------------------------------------------

DENND1A_BOVINE      --------------------------------------------------------------------------------GGCC
DENND1A.V1_HUMAN    ---GGCGAGGGAGCGAGCCTCGAGCGGGCGGGCCCCAGCCTGAGGGAAGGGAGGAAGGGGCGGGGAGAGCGCCAGAGGGAGGCC
DENND1A.X1_BOVINE   TGCGGCTGGGGAGCGAGACTCGAGCGGGCGGGC-TCAGCCTGAGGGAAGGGAGGAAGGGGCGGGGAGAGCGCCGGAGGGAGGCC
DENND1A.X2_BOVINE   TGCGGCTGGGGAGCGAGACTCGAGCGGGCGGGC-TCAGCCTGAGGGAAGGGAGGAAGGGGCGGGGAGAGCGCCGGAGGGAGGCC
DENND1A.X3_BOVINE   TGCGGCTGGGGAGCGAGACTCGAGCGGGCGGGC-TCAGCCTGAGGGAAGGGAGGAAGGGGCGGGGAGAGCGCCGGAGGGAGGCC
DENND1A.X4_BOVINE   TGCGGCTGGGGAGCGAGACTCGAGCGGGCGGGC-TCAGCCTGAGGGAAGGGAGGAAGGGGCGGGGAGAGCGCCGGAGGGAGGCC
DENND1A.X1-4_F      ------------------------------------------------------------------------------------
DENND1A.X1-4_R      ------------------------------------------------------------------------------------

DENND1A_BOVINE      GGTCGGCCGGTCGGCCGCAGGCGGGCGGGCAGCGCAGCGCAGCGCCGAGCGGGGCCCGCGGGCCCATGAGGAGGCCGGGGGACC
DENND1A.V1_HUMAN    GGTCG--------GCCGCGGGCGGGCGGGCAG-----CGCAGCGCCGAGCGGGGCCCGCGGGCCCATGAGGAGGCCTGGGGACC
DENND1A.X1_BOVINE   GGTCGGCCGGTCGGCCGCAGGCGGGCGGGCAGCGCAGCGCAGCGCCGAGCGGGGCCCGCGGGCCCATGAGGAGGCCGGGGGACC
DENND1A.X2_BOVINE   GGTCGGCCGGTCGGCCGCAGGCGGGCGGGCAGCGCAGCGCAGCGCCGAGCGGGGCCCGCGGGCCCATGAGGAGGCCGGGGGACC
DENND1A.X3_BOVINE   GGTCGGCCGGTCGGCCGCAGGCGGGCGGGCAGCGCAGCGCAGCGCCGAGCGGGGCCCGCGGGCCCATGAGGAGGCCGGGGGACC
DENND1A.X4_BOVINE   GGTCGGCCGGTCGGCCGCAGGCGGGCGGGCAGCGCAGCGCAGCGCCGAGCGGGGCCCGCGGGCCCATGAGGAGGCCGGGGGACC
DENND1A.X1-4_F      ------------------------------------------------------------------------------------
DENND1A.X1-4_R      ------------------------------------------------------------------------------------

DENND1A_BOVINE      ATGGGCTCCAGGATCAAgtgagtgtggtcggcct............cagtaatcacggtctgtttcttttgta
DENND1A.V1_HUMAN    ATGGGCTCCAGGATCAAgtgagtgcggccgggcc............cagtaatcatgatctgtttcttttgta
DENND1A.X1_BOVINE   ATGGGCTCCAGGATCAA-------------------------------------------------------------------
DENND1A.X2_BOVINE   ATGGGCTCCAGGATCAA-------------------------------------------------------------------
DENND1A.X3_BOVINE   ATGGGCTCCAGGATCAA-------------------------------------------------------------------
DENND1A.X4_BOVINE   ATGGGCTCCAGGATCAA-------------------------------------------------------------------
DENND1A.X1-4_F      ------------------------------------------------------------------------------------
DENND1A.X1-4_R      ------------------------------------------------------------------------------------
```

Exon 1

```
DENND1A_BOVINE      GCAAAACCCAGAGACCACCTTTGAAGTGTATGTTGAAGTGGCCTGTCCTAGGACAGGTGGCACTCTTTCAGgta......attta
DENND1A.V1_HUMAN    GCAGAATCCAGAGACCACATTTGAAGTATATGTTGAAGTGGCCTATCCCAGGACAGGTGGCACTCTTTCAGgta......attta
DENND1A.X1_BOVINE   GCAAAACCCAGAGACCACCTTTGAAGTGTATGTTGAAGTGGCCTGTCCTAGGACAGGTGGCACTCTTTCAG-------------
DENND1A.X2_BOVINE   GCAAAACCCAGAGACCACCTTTGAAGTGTATGTTGAAGTGGCCTGTCCTAGGACAGGTGGCACTCTTTCAG-------------
DENND1A.X3_BOVINE   GCAAAACCCAGAGACCACCTTTGAAGTGTATGTTGAAGTGGCCTGTCCTAGGACAGGTGGCACTCTTTCAG-------------
DENND1A.X4_BOVINE   GCAAAACCCAGAGACCACCTTTGAAGTGTATGTTGAAGTGGCCTGTCCTAGGACAGGTGGCACTCTTTCAG-------------
DENND1A.X1-4_F      ------------------------------------------------------------------------------------
DENND1A.X1-4_R      ------------------------------------------------------------------------------------
```

Exon 2

```
DENND1A_BOVINE      ATCCTGAGGTGCAGAGGCAATTCCCGGAGGACTACAGTGACCAGgttcggaatg............tttctctccacag
DENND1A.V1_HUMAN    ATCCTGAGGTGCAGAGGCAATTCCCGGAGGACTACAGTGACCAGgttcggaatg............tttctctccacag
DENND1A.X1_BOVINE   ATCCTGAGGTGCAGAGGCAATTCCCGGAGGACTACAGTGACCAG----------------------------------------
DENND1A.X2_BOVINE   ATCCTGAGGTGCAGAGGCAATTCCCGGAGGACTACAGTGACCAG----------------------------------------
DENND1A.X3_BOVINE   ATCCTGAGGTGCAGAGGCAATTCCCGGAGGACTACAGTGACCAG----------------------------------------
DENND1A.X4_BOVINE   ATCCTGAGGTGCAGAGGCAATTCCCGGAGGACTACAGTGACCAG----------------------------------------
DENND1A.X1-4_F      ------------------------------------------------------------------------------------
DENND1A.X1-4_R      ------------------------------------------------------------------------------------
```

Exon 3

```
DENND1A_BOVINE      GAAGTTCTACAGACTCTGACCAAGTTTTGTTTCCCCTTCTATGTGGACAGgtagtatcagattt..........ctttggcgcag
```

```
DENND1A.V1_HUMAN     GAAGTTCTACAGACTTTGACCAAGTTTTGTTTCCCCTTCTATGTGGACAGgtagtatcagattt……ctttgctgcag
DENND1A.X1_BOVINE    GAAGTTCTACAGACTCTGACCAAGTTTTGTTTCCCCTTCTATGTGGACAG----------------------------------
DENND1A.X2_BOVINE    GAAGTTCTACAGACTCTGACCAAGTTTTGTTTCCCCTTCTATGTGGACAG----------------------------------
DENND1A.X3_BOVINE    GAAGTTCTACAGACTCTGACCAAGTTTTGTTTCCCCTTCTATGTGGACAG----------------------------------
DENND1A.X4_BOVINE    GAAGTTCTACAGACTCTGACCAAGTTTTGTTTCCCCTTCTATGTGGACAG----------------------------------
DENND1A.X1-4_F       ------------------------------------------------------------------------------------
DENND1A.X1-4_R       ------------------------------------------------------------------------------------
```
Exon 4

```
DENND1A_BOVINE       CCTCACAGTTAGCCAAGTTGGCCAGAACTTCACATTCGTGCTCACTGACATTGACAGCAAACAGAGATTCGGGTTCTGCCGCTT
DENND1A.V1_HUMAN     CCTCACAGTTAGCCAAGTTGGCCAGAACTTCACATTCGTGCTCACTGACATTGACAGCAAACAGAGATTCGGGTTCTGCCGCTT
DENND1A.X1_BOVINE    CCTCACAGTTAGCCAAGTTGGCCAGAACTTCACATTCGTGCTCACTGACATTGACAGCAAACAGAGATTCGGGTTCTGCCGCTT
DENND1A.X2_BOVINE    CCTCACAGTTAGCCAAGTTGGCCAGAACTTCACATTCGTGCTCACTGACATTGACAGCAAACAGAGATTCGGGTTCTGCCGCTT
DENND1A.X3_BOVINE    CCTCACAGTTAGCCAAGTTGGCCAGAACTTCACATTCGTGCTCACTGACATTGACAGCAAACAGAGATTCGGGTTCTGCCGCTT
DENND1A.X4_BOVINE    CCTCACAGTTAGCCAAGTTGGCCAGAACTTCACATTCGTGCTCACTGACATTGACAGCAAACAGAGATTCGGGTTCTGCCGCTT
DENND1A.X1-4_F       ------------------------------------------------------------------------------------
DENND1A.X1-4_R       ------------------------------------------------------------------------------------

DENND1A_BOVINE       ATCTTCAGGAGCAAAGAGTTGCTTCTGTATCTTAAGgtaaggaaaatgg……tcttcttccctcag
DENND1A.V1_HUMAN     ATCTTCAGGAGCGAAGAGCTGCTTCTGTATCTTAAGgtaagggagaagg……tcttcttcctccag
DENND1A.X1_BOVINE    ATCTTCAGGAGCAAAGAGTTGCTTCTGTATCTTAAG------------------------------------------------
DENND1A.X2_BOVINE    ATCTTCAGGAGCAAAGAGTTGCTTCTGTATCTTAAG------------------------------------------------
DENND1A.X3_BOVINE    ATCTTCAGGAGCAAAGAGTTGCTTCTGTATCTTAAG------------------------------------------------
DENND1A.X4_BOVINE    ATCTTCAGGAGCAAAGAGTTGCTTCTGTATCTTAAG------------------------------------------------
DENND1A.X1-4_F       ------------------------------------------------------------------------------------
DENND1A.X1-4_R       ------------------------------------------------------------------------------------
```
Exon 5

```
DENND1A_BOVINE       CTATCTCCCCTGGTTCGAAGTATTTTATAAGCTACTTAACATCTTGGCAGATTACACGACAAAAGGACAGgtat……tttcag
DENND1A.V1_HUMAN     CTATCTCCCCTGGTTCGAGGTATTTTATAAGCTGCTTAACATCCTGGCAGATTACACGACAAAAAGACAGgtat……tttcag
DENND1A.X1_BOVINE    CTATCTCCCCTGGTTCGAAGTATTTTATAAGCTACTTAACATCTTGGCAGATTACACGACAAAAGGACAG--------------
DENND1A.X2_BOVINE    CTATCTCCCCTGGTTCGAAGTATTTTATAAGCTACTTAACATCTTGGCAGATTACACGACAAAAGGACAG--------------
DENND1A.X3_BOVINE    CTATCTCCCCTGGTTCGAAGTATTTTATAAGCTACTTAACATCTTGGCAGATTACACGACAAAAGGACAG--------------
DENND1A.X4_BOVINE    CTATCTCCCCTGGTTCGAAGTATTTTATAAGCTACTTAACATCTTGGCAGATTACACGACAAAAGGACAG--------------
DENND1A.X1-4_F       ------------------------------------------------------------------------------------
DENND1A.X1-4_R       ------------------------------------------------------------------------------------
```
Exon 6

```
DENND1A_BOVINE       GAGAGTCAGTGGAATGAGCTTCTTGAAACTCTGTACAAACTTCCTATCCCTGACCCAGGAGTGTCTGTTCATCTCAGTGTGg…g
DENND1A.V1_HUMAN     GAAAATCAGTGGAATGAGCTTCTTGAAACTCTGCACAAACTTCCCATCCCTGACCCAGGAGTGTCTGTCCATCTCAGCGTGg…g
DENND1A.X1_BOVINE    GAGAGTCAGTGGAATGAGCTTCTTGAAACTCTGTACAAACTTCCTATCCCTGACCCAGGAGTGTCTGTTCATCTCAGTGTG---
DENND1A.X2_BOVINE    GAGAGTCAGTGGAATGAGCTTCTTGAAACTCTGTACAAACTTCCTATCCCTGACCCAGGAGTGTCTGTTCATCTCAGTGTG---
DENND1A.X3_BOVINE    GAGAGTCAGTGGAATGAGCTTCTTGAAACTCTGTACAAACTTCCTATCCCTGACCCAGGAGTGTCTGTTCATCTCAGTGTG---
DENND1A.X4_BOVINE    GAGAGTCAGTGGAATGAGCTTCTTGAAACTCTGTACAAACTTCCTATCCCTGACCCAGGAGTGTCTGTTCATCTCAGTGTG---
DENND1A.X1-4_F       ------------------------------------------------------------------------------------
DENND1A.X1-4_R       ------------------------------------------------------------------------------------
```
Exon 7

```
DENND1A_BOVINE       CATTCTTATTTTACTGTGCCTGATACCAGAGAACTTCCCAGCATCCCTGAGAATgtaagtactt……tttctcctag
DENND1A.V1_HUMAN     CATTCTTATTTTACTGTGCCTGATACCAGAGAACTTCCCAGCATACCTGAGAATgtaagtactt……tttctcctag
DENND1A.X1_BOVINE    CATTCTTATTTTACTGTGCCTGATACCAGAGAACTTCCCAGCATCCCTGAGAAT------------------------------
DENND1A.X2_BOVINE    CATTCTTATTTTACTGTGCCTGATACCAGAGAACTTCCCAGCATCCCTGAGAAT------------------------------
DENND1A.X3_BOVINE    CATTCTTATTTTACTGTGCCTGATACCAGAGAACTTCCCAGCATCCCTGAGAAT------------------------------
DENND1A.X4_BOVINE    CATTCTTATTTTACTGTGCCTGATACCAGAGAACTTCCCAGCATCCCTGAGAAT------------------------------
DENND1A.X1-4_F       ------------------------------------------------------------------------------------
DENND1A.X1-4_R       ------------------------------------------------------------------------------------
```
Exon 8

```
DENND1A_BOVINE       AGAAATCTGACAGAATATTTTGTGGCTGTGGATGTAAACAACATGTTACATCTGTATGCCAGTATGCTCTACGAACGCCGGATA
DENND1A.V1_HUMAN     AGAAATCTGACAGAATATTTTGTGGCTGTGGATGTTAACAACATGTTGCATCTGTACGCCAGTATGCTGTACGAACGCCGGATA
DENND1A.X1_BOVINE    AGAAATCTGACAGAATATTTTGTGGCTGTGGATGTAAACAACATGTTACATCTGTATGCCAGTATGCTCTACGAACGCCGGATA
DENND1A.X2_BOVINE    AGAAATCTGACAGAATATTTTGTGGCTGTGGATGTAAACAACATGTTACATCTGTATGCCAGTATGCTCTACGAACGCCGGATA
DENND1A.X3_BOVINE    AGAAATCTGACAGAATATTTTGTGGCTGTGGATGTAAACAACATGTTACATCTGTATGCCAGTATGCTCTACGAACGCCGGATA
DENND1A.X4_BOVINE    AGAAATCTGACAGAATATTTTGTGGCTGTGGATGTAAACAACATGTTACATCTGTATGCCAGTATGCTCTACGAACGCCGGATA
DENND1A.X1-4_F       ------------------------------------------------------------------------------------
DENND1A.X1-4_R       ------------------------------------------------------------------------------------

DENND1A_BOVINE       CTCATCATTTGCAGCAAACTCAGCACTgtgagtagacagtctt……aatctttaatctctgttgcag
DENND1A.V1_HUMAN     CTCATCATTTGCAGCAAACTCAGCACTgtgagtagacagtctt……ctctgctttgctctgttccag
DENND1A.X1_BOVINE    CTCATCATTTGCAGCAAACTCAGCACT---------------------------------------------------------
DENND1A.X2_BOVINE    CTCATCATTTGCAGCAAACTCAGCACT---------------------------------------------------------
DENND1A.X3_BOVINE    CTCATCATTTGCAGCAAACTCAGCACT---------------------------------------------------------
DENND1A.X4_BOVINE    CTCATCATTTGCAGCAAACTCAGCACT---------------------------------------------------------
DENND1A.X1-4_F       ------------------------------------------------------------------------------------
DENND1A.X1-4_R       ------------------------------------------------------------------------------------
```
Exon 9

```
DENND1A_BOVINE      TTGACTGCCTGCATCCACGGGTCTGCTGCGATGCTCTACCCCATGTTCTGGCAGCACGTGTACATCCCTGTCCTGCCTCCACAT
DENND1A.V1_HUMAN    CTGACTGCCTGCATCCACGGGTCTGCGGCGATGCTCTACCCCATGTACTGGCAGCACGTGTACATCCCCGTGCTGCCGCCGCAT
DENND1A.X1_BOVINE   TTGACTGCCTGCATCCACGGGTCTGCTGCGATGCTCTACCCCATGTTCTGGCAGCACGTGTACATCCCTGTCCTGCCTCCACAT
DENND1A.X2_BOVINE   TTGACTGCCTGCATCCACGGGTCTGCTGCGATGCTCTACCCCATGTTCTGGCAGCACGTGTACATCCCTGTCCTGCCTCCACAT
DENND1A.X3_BOVINE   TTGACTGCCTGCATCCACGGGTCTGCTGCGATGCTCTACCCCATGTTCTGGCAGCACGTGTACATCCCTGTCCTGCCTCCACAT
DENND1A.X4_BOVINE   TTGACTGCCTGCATCCACGGGTCTGCTGCGATGCTCTACCCCATGTTCTGGCAGCACGTGTACATCCCTGTCCTGCCTCCACAT
DENND1A.X1-4_F      ------------------------------------------------------------------------------------
DENND1A.X1-4_R      ------------------------------------------------------------------------------------

DENND1A_BOVINE      CTGCTGGACTACTGCTGgtaaggccactg.......................................gtctctgtttctcttcacag
DENND1A.V1_HUMAN    CTGCTGGACTACTGCTGgtaaggccactg.......................................gtctctgtttctcttcacag
DENND1A.X1_BOVINE   CTGCTGGACTACTGCTG-------------------------------------------------------------------
DENND1A.X2_BOVINE   CTGCTGGACTACTGCTG-------------------------------------------------------------------
DENND1A.X3_BOVINE   CTGCTGGACTACTGCTG-------------------------------------------------------------------
DENND1A.X4_BOVINE   CTGCTGGACTACTGCTG-------------------------------------------------------------------
DENND1A.X1-4_F      ------------------------------------------------------------------------------------
DENND1A.X1-4_R      ------------------------------------------------------------------------------------
```

Exon 10

```
DENND1A_BOVINE      TGCTCCCATGCCCTACCTCATAGGAATCCATTTAAGTTTAATGGAGgtaagttgacttct............ttttacttgcag
DENND1A.V1_HUMAN    TGCTCCCATGCCCTACCTCATAGGAATCCATTTAAGTTTAATGGAGgtaagttgacttct............ttctccttgcag
DENND1A.X1_BOVINE   TGCTCCCATGCCCTACCTCATAGGAATCCATTTAAGTTTAATGGAG--------------------------------------
DENND1A.X2_BOVINE   TGCTCCCATGCCCTACCTCATAGGAATCCATTTAAGTTTAATGGAG--------------------------------------
DENND1A.X3_BOVINE   TGCTCCCATGCCCTACCTCATAGGAATCCATTTAAGTTTAATGGAG--------------------------------------
DENND1A.X4_BOVINE   TGCTCCCATGCCCTACCTCATAGGAATCCATTTAAGTTTAATGGAG--------------------------------------
DENND1A.X1-4_F      ------------------------------------------------------------------------------------
DENND1A.X1-4_R      ------------------------------------------------------------------------------------
```

Exon 11

```
DENND1A_BOVINE      AAAGTCAGAAGCATGGCCCTGGATGATGTCGTGATCCTGAATGTGGACACCAACACCCTGGAAACCCCCTTTGATGACCTCCAG
DENND1A.V1_HUMAN    AAAGTCAGAAACATGGCCCTGGATGATGTCGTGATCCTGAATGTGGACACCAACACCCTGGAAACCCCCTTCGATGACCTCCAG
DENND1A.X1_BOVINE   AAAGTCAGAAGCATGGCCCTGGATGATGTCGTGATCCTGAATGTGGACACCAACACCCTGGAAACCCCCTTTGATGACCTCCAG
DENND1A.X2_BOVINE   AAAGTCAGAAGCATGGCCCTGGATGATGTCGTGATCCTGAATGTGGACACCAACACCCTGGAAACCCCCTTTGATGACCTCCAG
DENND1A.X3_BOVINE   AAAGTCAGAAGCATGGCCCTGGATGATGTCGTGATCCTGAATGTGGACACCAACACCCTGGAAACCCCCTTTGATGACCTCCAG
DENND1A.X4_BOVINE   AAAGTCAGAAGCATGGCCCTGGATGATGTCGTGATCCTGAATGTGGACACCAACACCCTGGAAACCCCCTTTGATGACCTCCAG
DENND1A.X1-4_F      --------------------------TGTCGTGATCCTGAATGTGG--------------------------------------
DENND1A.X1-4_R      ---------------------------------------------------------------------------------CAG

DENND1A_BOVINE      AGCCTCCCAAATGATGTGgtgggtaatgagctctcgagt............................ctcctttctgtcatcag
DENND1A.V1_HUMAN    AGCCTCCCAAACGACGTGgtaggtaatgagcttgcgagg............................cttctctccatcaccag
DENND1A.X1_BOVINE   AGCCTCCCAAATGATGTG------------------------------------------------------------------
DENND1A.X2_BOVINE   AGCCTCCCAAATGATGTG------------------------------------------------------------------
DENND1A.X3_BOVINE   AGCCTCCCAAATGATGTG------------------------------------------------------------------
DENND1A.X4_BOVINE   AGCCTCCCAAATGATGTG------------------------------------------------------------------
DENND1A.X1-4_F      ------------------------------------------------------------------------------------
DENND1A.X1-4_R      AGCCTCCCAAATGATGT-------------------------------------------------------------------
```

Exon 12

```
DENND1A_BOVINE      ATCTCTTCCTTGAAGAGCCGGCTGAAGAAGGTGTCCACGACAACTGGTGATGGTGTGGCCAGAGCCTTCCTCAAGGCCCAGGCC
DENND1A.V1_HUMAN    ATCTCTTCCCTGAAGAACAGGCTGAAAAAGGTCTCCACAACCACTGGGGATGGTGTGGCCAGAGCGTTCCTCAAGGCCCAGGCT
DENND1A.X1_BOVINE   ATCTCTTCCTTGAAGAGCCGGCTGAAGAAGGTGTCCACGACAACTGGTGATGGTGTGGCCAGAGCCTTCCTCAAGGCCCAGGCC
DENND1A.X2_BOVINE   ATCTCTTCCTTGAAGAGCCGGCTGAAGAAGGTGTCCACGACAACTGGTGATGGTGTGGCCAGAGCCTTCCTCAAGGCCCAGGCC
DENND1A.X3_BOVINE   ATCTCTTCCTTGAAGAGCCGGCTGAAGAAGGTGTCCACGACAACTGGTGATGGTGTGGCCAGAGCCTTCCTCAAGGCCCAGGCC
DENND1A.X4_BOVINE   ATCTCTTCCTTGAAGAGCCGGCTGAAGAAGGTGTCCACGACAACTGGTGATGGTGTGGCCAGAGCCTTCCTCAAGGCCCAGGCC
DENND1A.X1-4_F      ------------------------------------------------------------------------------------
DENND1A.X1-4_R      ------------------------------------------------------------------------------------

DENND1A_BOVINE      GCTTTCTTCGGCAGCTACCGAAACGCTCTGAAAATCGAGCCGgtgagtagc......................tctctgtctcccag
DENND1A.V1_HUMAN    GCTTTCTTCGGTAGCTACCGAAACGCTCTGAAAATCGAGCCGgtgagtagc......................tctctgtctcccag
DENND1A.X1_BOVINE   GCTTTCTTCGGCAGCTACCGAAACGCTCTGAAAATCGAGCCG------------------------------------------
DENND1A.X2_BOVINE   GCTTTCTTCGGCAGCTACCGAAACGCTCTGAAAATCGAGCCG------------------------------------------
DENND1A.X3_BOVINE   GCTTTCTTCGGCAGCTACCGAAACGCTCTGAAAATCGAGCCG------------------------------------------
DENND1A.X4_BOVINE   GCTTTCTTCGGCAGCTACCGAAACGCTCTGAAAATCGAGCCG------------------------------------------
DENND1A.X1-4_F      ------------------------------------------------------------------------------------
DENND1A.X1-4_R      ------------------------------------------------------------------------------------
```

Exon 13

```
DENND1A_BOVINE      GAGGAGCCAATCACCTTCTGCGAGGAAGCCTTCGTGTCGCACTATCGCTCAGGAGCCATGAGGCAGTTCCTGCAGAATGCCACC
DENND1A.V1_HUMAN    GAGGAGCCGATCACTTTCTGTGAGGAAGCCTTCGTGTCCCACTACCGCTCCGGAGCCATGAGGCAGTTCCTGCAGAACGCCACA
DENND1A.X1_BOVINE   GAGGAGCCAATCACCTTCTGCGAGGAAGCCTTCGTGTCGCACTATCGCTCAGGAGCCATGAGGCAGTTCCTGCAGAATGCCACC
DENND1A.X2_BOVINE   GAGGAGCCAATCACCTTCTGCGAGGAAGCCTTCGTGTCGCACTATCGCTCAGGAGCCATGAGGCAGTTCCTGCAGAATGCCACC
DENND1A.X3_BOVINE   GAGGAGCCAATCACCTTCTGCGAGGAAGCCTTCGTGTCGCACTATCGCTCAGGAGCCATGAGGCAGTTCCTGCAGAATGCCACC
DENND1A.X4_BOVINE   GAGGAGCCAATCACCTTCTGCGAGGAAGCCTTCGTGTCGCACTATCGCTCAGGAGCCATGAGGCAGTTCCTGCAGAATGCCACC
DENND1A.X1-4_F      ------------------------------------------------------------------------------------
DENND1A.X1-4_R      ------------------------------------------------------------------------------------
```

Exon 14

```
DENND1A_BOVINE      CAGCTGCAGCTCTTTAAACAGgtgcccagcctcccttg.......ttctttctttgtatag
DENND1A.V1_HUMAN    CAGCTGCAGCTCTTCAAGCAGgtgcctc-cctccctgg.......ttccttctttctatag
DENND1A.X1_BOVINE   CAGCTGCAGCTCTTTAAACAG--------------------------------------------------------------
DENND1A.X2_BOVINE   CAGCTGCAGCTCTTTAAACAG--------------------------------------------------------------
DENND1A.X3_BOVINE   CAGCTGCAGCTCTTTAAACAG--------------------------------------------------------------
DENND1A.X4_BOVINE   CAGCTGCAGCTCTTTAAACAG--------------------------------------------------------------
DENND1A.X1-4_F      ----------------------------------------------------------------------------------
DENND1A.X1-4_R      ----------------------------------------------------------------------------------
                                                                                     Exon 14

DENND1A_BOVINE      TTTATCGATGGTAGATTAGACCTTCTCAATTCTGGCGAGGGCTTCAGTGATGTTTTTGAAGAGGAGATCAACATGAGCGAGTAT
DENND1A.V1_HUMAN    TTTATTGATGGTCGATTAGATCTTCTCAATTCCGGCGAAGGTTTCAGTGATGTTTTTGAAGAGGAAATCAACATGGGCGAGTAC
DENND1A.X1_BOVINE   TTTATCGATGGTAGATTAGACCTTCTCAATTCTGGCGAGGGCTTCAGTGATGTTTTTGAAGAGGAGATCAACATGAGCGAGTAT
DENND1A.X2_BOVINE   TTTATCGATGGTAGATTAGACCTTCTCAATTCTGGCGAGGGCTTCAGTGATGTTTTTGAAGAGGAGATCAACATGAGCGAGTAT
DENND1A.X3_BOVINE   TTTATCGATGGTAGATTAGACCTTCTCAATTCTGGCGAGGGCTTCAGTGATGTTTTTGAAGAGGAGATCAACATGAGCGAGTAT
DENND1A.X4_BOVINE   TTTATCGATGGTAGATTAGACCTTCTCAATTCTGGCGAGGGCTTCAGTGATGTTTTTGAAGAGGAGATCAACATGAGCGAGTAT
DENND1A.X1-4_F      ----------------------------------------------------------------------------------
DENND1A.X1-4_R      ----------------------------------------------------------------------------------

DENND1A_BOVINE      GCTGgtaagg-gcagttcattttcttta.......cttctctgcctcctctttccccag
DENND1A.V1_HUMAN    GCTGgtgagaagcaactcattttccttcc.......cttctctgaattctctttccccag
DENND1A.X1_BOVINE   GCTG------------------------------------------------------------------------------
DENND1A.X2_BOVINE   GCTG------------------------------------------------------------------------------
DENND1A.X3_BOVINE   GCTG------------------------------------------------------------------------------
DENND1A.X4_BOVINE   GCTG------------------------------------------------------------------------------
DENND1A.X1-4_F      ----------------------------------------------------------------------------------
DENND1A.X1-4_R      ----------------------------------------------------------------------------------
                                                                                     Exon 15

DENND1A_BOVINE      GGAGTGATAAGCTATACCACCAGTGGCTCTCCACAGTCCGGgtaag–tgccaccccc.......tttttattatag
DENND1A.V1_HUMAN    GCAGTGACAAACTGTACCATCAGTGGCTCTCCACTGTCCGGgtaagcatgcacccaa.......tttttattatag
DENND1A.X1_BOVINE   GGAGTGATAAGCTATACCACCAGTGGCTCTCCACAGTCCGG-----------------------------------------
DENND1A.X2_BOVINE   GGAGTGATAAGCTATACCACCAGTGGCTCTCCACAGTCCGG-----------------------------------------
DENND1A.X3_BOVINE   GGAGTGATAAGCTATACCACCAGTGGCTCTCCACAGTCCGG-----------------------------------------
DENND1A.X4_BOVINE   GGAGTGATAAGCTATACCACCAGTGGCTCTCCACAGTCCGG-----------------------------------------
DENND1A.X1-4_F      ----------------------------------------------------------------------------------
DENND1A.X1-4_R      ----------------------------------------------------------------------------------
                                                                                     Exon 16

DENND1A_BOVINE      AAAGGAAGTGGAGCAATTTTGAATACTGTAAAAACGAAAGCAAACCCGGCCATGAAGACTGTCTATAAGTTCgtaa.......gcag
DENND1A.V1_HUMAN    AAAGGAAGTGGAGCAATTCTGAATACTGTAAAGACCAAAGCAAATCCGGCCATGAAGACTGTCTACAAGTTCgtaa.......gcag
DENND1A.X1_BOVINE   AAAGGAAGTGGAGCAATTTTGAATACTGTAAAAACGAAAGCAAACCCGGCCATGAAGACTGTCTATAAGTTC------------
DENND1A.X2_BOVINE   AAAGGAAGTGGAGCAATTTTGAATACTGTAAAAACGAAAGCAAACCCGGCCATGAAGACTGTCTATAAGTTC------------
DENND1A.X3_BOVINE   AAAGGAAGTGGAGCAATTTTGAATACTGTAAAAACGAAAGCAAACCCGGCCATGAAGACTGTCTATAAGTTC------------
DENND1A.X4_BOVINE   AAAGGAAGTGGAGCAATTTTGAATACTGTAAAAACGAAAGCAAACCCGGCCATGAAGACTGTCTATAAGTTC------------
DENND1A.X1-4_F      ----------------------------------------------------------------------------------
DENND1A.X1-4_R      ----------------------------------------------------------------------------------
                                                                                     Exon 17

DENND1A_BOVINE      GCAAAAGATCATGCAAAAATGGGAATAAAAGAGGTGAAAAACCGCTTGAAGCAAAAGgtacttgaag.......tccttgcag
DENND1A.V1_HUMAN    GCAAAAGATCATGCAAAAATGGGAATAAAAGAGGTGAAAAACCGCTTGAAGCAAAAGgtacttgaag.......tccctacag
DENND1A.X1_BOVINE   GCAAAAGATCATGCAAAAATGGGAATAAAAGAGGTGAAAAACCGCTTGAAGCAAAAG-------------------------
DENND1A.X2_BOVINE   GCAAAAGATCATGCAAAAATGGGAATAAAAGAGGTGAAAAACCGCTTGAAGCAAAAG-------------------------
DENND1A.X3_BOVINE   GCAAAAGATCATGCAAAAATGGGAATAAAAGAGGTGAAAAACCGCTTGAAGCAAAAG-------------------------
DENND1A.X4_BOVINE   GCAAAAGATCATGCAAAAATGGGAATAAAAGAGGTGAAAAACCGCTTGAAGCAAAAG-------------------------
DENND1A.X1-4_F      ----------------------------------------------------------------------------------
DENND1A.X1-4_R      ----------------------------------------------------------------------------------
                                                                                     Exon 18

DENND1A_BOVINE      GACATCACTGAGAATGGCTGTGCCCCCACCACAGAAGAGCAGCTGCCAAAGACTGTGCCGTCCCCACTGGTAGAGGCCAAGGAC
DENND1A.V1_HUMAN    GACATTGCCGAGAATGGCTGCGCCCCCACCCCAGAAGAGCAGCTGCCAAAGACTGCACCGTCCCCACTGGTGGAGGCCAAGGAC
DENND1A.X1_BOVINE   GACATCACTGAGAATGGCTGTGCCCCCACCACAGAAGAGCAGCTGCCAAAGACTGTGCCGTCCCCACTGGTAGAGGCCAAGGAC
DENND1A.X2_BOVINE   GACATCACTGAGAATGGCTGTGCCCCCACCACAGAAGAGCAGCTGCCAAAGACTGTGCCGTCCCCACTGGTAGAGGCCAAGGAC
DENND1A.X3_BOVINE   GACATCACTGAGAATGGCTGTGCCCCCACCACAGAAGAGCAGCTGCCAAAGACTGTGCCGTCCCCACTGGTAGAGGCCAAGGAC
DENND1A.X4_BOVINE   GACATCACTGAGAATGGCTGTGCCCCCACCACAGAAGAGCAGCTGCCAAAGACTGTGCCGTCCCCACTGGTAGAGGCCAAGGAC
DENND1A.X1-4_F      ----------------------------------------------------------------------------------
DENND1A.X1-4_R      ----------------------------------------------------------------------------------

DENND1A_BOVINE      CCCAAGTTCCGAGAGGACCGGCGGCCAATCACAGTCCACTTTGGACAGgtatgta.......cagccacagagactccgtcccactc
DENND1A.V1_HUMAN    CCCAAGCTCCGAGAAGACCGGCGGCCAATCACAGTCCACTTTGGACAGgtgtgta.......cagctgcagagactgcgtcccaccc
DENND1A.X1_BOVINE   CCCAAGTTCCGAGAGGACCGGCGGCCAATCACAGTCCACTTTGGACAG--------------CCACAGAGACTCCGTCCCACTC
DENND1A.X2_BOVINE   CCCAAGTTCCGAGAGGACCGGCGGCCAATCACAGTCCACTTTGGACAG----------------------------------
DENND1A.X3_BOVINE   CCCAAGTTCCGAGAGGACCGGCGGCCAATCACAGTCCACTTTGGACAG--------------CCACAGAGACTCCGTCCCACTC
                                                                                     Exon 19
```

```
DENND1A.X4_BOVINE     CCCAAGTTCCGAGAGGACCGGCGGCCAATCACAGTCCACTTTGGACAG------------------------------------
DENND1A.X1-4_F        ------------------------------------------------------------------------------------
DENND1A.X1-4_R        ------------------------------------------------------------------------------------

DENND1A_BOVINE        gcccactgcctcccaagatacagcgctcgaggcccgtgagtag…attttcccc-atgcccattctag
DENND1A.V1_HUMAN      g---accgcctcccaagatacagcgctcgaggcccgtgagtag…ctctccccctgtacctcctctag
DENND1A.X1_BOVINE     GCCCACTGCCTCCCAAGATACAGCGCTCGAGGCCC-------------------------------------------------
DENND1A.X2_BOVINE     ------------------------------------------------------------------------------------
DENND1A.X3_BOVINE     GCCCACTGCCTCCCAAGATACAGCGCTCGAGGCCC-------------------------------------------------
DENND1A.X4_BOVINE     ------------------------------------------------------------------------------------
DENND1A.X1-4_F        ------------------------------------------------------------------------------------
DENND1A.X1-4_R        ------------------------------------------------------------------------------------
                                                                                                   Exon 19

DENND1A_BOVINE        GTGCGCCCTCCCCGTCCACACGTCGTTAAAAGACCGAAGAGCAACATCACAGTGGAAGCCCGAAGGACGTCCGTCTCGAGCCCC
DENND1A.V1_HUMAN      GTGCGCCCACCTCGTCCACATGTTGTTAAGAGACCAAAGAGCAACATCGCAGTGGAAGGCCGGAGGACGTCTGTGCCGAGCCCT
DENND1A.X1_BOVINE     GTGCGCCCTCCCCGTCCACACGTCGTTAAAAGACCGAAGAGCAACATCACAGTGGAAGCCCGAAGGACGTCCGTCTCGAGCCCC
DENND1A.X2_BOVINE     GTGCGCCCTCCCCGTCCACACGTCGTTAAAAGACCGAAGAGCAACATCACAGTGGAAGCCCGAAGGACGTCCGTCTCGAGCCCC
DENND1A.X3_BOVINE     GTGCGCCCTCCCCGTCCACACGTCGTTAAAAGACCGAAGAGCAACATCACAGTGGAAGCCCGAAGGACGTCCGTCTCGAGCCCC
DENND1A.X4_BOVINE     ------------------------------------------------------------------------------------
DENND1A.X1-4_F        ------------------------------------------------------------------------------------
DENND1A.X1-4_R        ------------------------------------------------------------------------------------

DENND1A_BOVINE        GACCAgtgagtac…gccccagcctcgtaaggcccttgcgacactatgctgtcttcctctccgaagactcctctgacgatg
DENND1A.V1_HUMAN      GAGCAgtgagtat…gccccagcctggtaaagcccttgcgacactatgcggtcttcctctccgaagactcctctgatgatg
DENND1A.X1_BOVINE     GACCA--------------------CCTCGTAAGGCCCTTGCGACACTATGCTGTCTTCCTCTCCGAAGACTCCTCTGACGATG
DENND1A.X2_BOVINE     GACCA--------------------CCTCGTAAGGCCCTTGCGACACTATGCTGTCTTCCTCTCCGAAGACTCCTCTGACGATG
DENND1A.X3_BOVINE     GACCA-------------------------------------------------------------------------------
DENND1A.X4_BOVINE     ------------------------------------------------------------------------------------
DENND1A.X1-4_F        ------------------------------------------------------------------------------------
DENND1A.X1-4_R        ------------------------------------------------------------------------------------
                                                                                                   Exon 20

DENND1A_BOVINE        agtgccagcgggaagagggcccgagctctggcttcaccgaaagctttttcttctccactccctttgaatggtctc…cccccag
DENND1A.V1_HUMAN      aatgccagcgggaagagggcccgagctctggcttcaccgagagctttttcttctccgctccctttgaatggtctc…cccgacag
DENND1A.X1_BOVINE     AGTGCCAGCGGGAAGAGGGCCCGAGCTCTGGCTTCACCGAAAGCTTTTTCTTCTCCACTCCCTTTGAATGG-------------
DENND1A.X2_BOVINE     AGTGCCAGCGGGAAGAGGGCCCGAGCTCTGGCTTCACCGAAAGCTTTTTCTTCTCCACTCCCTTTGAATGG-------------
DENND1A.X3_BOVINE     ------------------------------------------------------------------------------------
DENND1A.X4_BOVINE     ------------------------------------------------------------------------------------
DENND1A.X1-4_F        ------------------------------------------------------------------------------------
DENND1A.X1-4_R        ------------------------------------------------------------------------------------

DENND1A_BOVINE        GCCACAGCCATATCGGACACTCAAGGAGTCAGACAGTGC---AGGGGACGAGGCCGAAAGCCCGGAGCAGCGAGCGCGGGAGCC
DENND1A.V1_HUMAN      GCCGCAGCCGTATCGGACACTCAGGGAGTCAGACAGCGCGGAAGGCGACGAGGCAGAGAGTCCAGAGCAGCAAGTGCGGAAGTC
DENND1A.X1_BOVINE     -CCACAGCCATATCGGACACTCAAGGAGTCAGACAGTGC---AGGGGACGAGGCCGAAAGCCCGGAGCAGCGAGCGCGGGAGCC
DENND1A.X2_BOVINE     -CCACAGCCATATCGGACACTCAAGGAGTCAGACAGTGC---AGGGGACGAGGCCGAAAGCCCGGAGCAGCGAGCGCGGGAGCC
DENND1A.X3_BOVINE     GCCACAGCCATATCGGACACTCAAGGAGTCAGACAGTGC---AGGGGACGAGGCCGAAAGCCCGGAGCAGCGAGCGCGGGAGCC
DENND1A.X4_BOVINE     GCCACAGCCATATCGGACACTCAAGGAGTCAGACAGTGC---AGGGGACGAGGCCGAAAGCCCGGAGCAGCGAGCGCGGGAGCC
DENND1A.X1-4_F        ------------------------------------------------------------------------------------
DENND1A.X1-4_R        ------------------------------------------------------------------------------------

DENND1A_BOVINE        TGTGGGCCCCACCCCAGCTCCACACGACCGGGCCGCCAGCATCAACCTCCTGGAGGATGTCTTCAGCAACCTCGACATGGAAGT
DENND1A.V1_HUMAN      CACAGGCCCTGTCCCAGCTCCCCCTGACCGGGCTGCCAGCATCGACCTTCTGGAAGACGTCTTCAGCAACCTGGACATGGAGGC
DENND1A.X1_BOVINE     TGTGGGCCCCACCCCAGCTCCACACGACCGGGCCGCCAGCATCAACCTCCTGGAGGATGTCTTCAGCAACCTCGACATGGAAGT
DENND1A.X2_BOVINE     TGTGGGCCCCACCCCAGCTCCACACGACCGGGCCGCCAGCATCAACCTCCTGGAGGATGTCTTCAGCAACCTCGACATGGAAGT
DENND1A.X3_BOVINE     TGTGGGCCCCACCCCAGCTCCACACGACCGGGCCGCCAGCATCAACCTCCTGGAGGATGTCTTCAGCAACCTCGACATGGAAGT
DENND1A.X4_BOVINE     TGTGGGCCCCACCCCAGCTCCACACGACCGGGCCGCCAGCATCAACCTCCTGGAGGATGTCTTCAGCAACCTCGACATGGAAGT
DENND1A.X1-4_F        ------------------------------------------------------------------------------------
DENND1A.X1-4_R        ------------------------------------------------------------------------------------
                                                                                                   Exon 21

DENND1A_BOVINE        CCCGCTGCAGCAGCTGGGCCAGGCCAAAAGCTTGGAAGACCTTCGGACCCCCAAAGACCTGAGGGAGCAGCCGGGGACCTTTGA
DENND1A.V1_HUMAN      CGCACTGCAGCCACTGGGCCAGGCCAAGAGCTTAGAGGACCTTCGTGCCCCCAAAGACCTGAGGGAGCAGCCAGGGACCTTTGA
DENND1A.X1_BOVINE     CCCGCTGCAGCAGCTGGGCCAGGCCAAAAGCTTGGAAGACCTTCGGACCCCCAAAGACCTGAGGGAGCAGCCGGGGACCTTTGA
DENND1A.X2_BOVINE     CCCGCTGCAGCAGCTGGGCCAGGCCAAAAGCTTGGAAGACCTTCGGACCCCCAAAGACCTGAGGGAGCAGCCGGGGACCTTTGA
DENND1A.X3_BOVINE     CCCGCTGCAGCAGCTGGGCCAGGCCAAAAGCTTGGAAGACCTTCGGACCCCCAAAGACCTGAGGGAGCAGCCGGGGACCTTTGA
DENND1A.X4_BOVINE     CCCGCTGCAGCAGCTGGGCCAGGCCAAAAGCTTGGAAGACCTTCGGACCCCCAAAGACCTGAGGGAGCAGCCGGGGACCTTTGA
DENND1A.X1-4_F        ------------------------------------------------------------------------------------
DENND1A.X1-4_R        ------------------------------------------------------------------------------------

DENND1A_BOVINE        CTATCAGgtatgg--tggttggag…gcagtcattgcctccctcgctctctcc-ggcag
DENND1A.V1_HUMAN      CTATCAGgtatggcatgggcaagg…gtggtcactgccgccctccttccctcccaacag
DENND1A.X1_BOVINE     CTATCAG-----------------------------------------------------------------------------
DENND1A.X2_BOVINE     CTATCAG-----------------------------------------------------------------------------
```

```
DENND1A.X3_BOVINE      CTATCAG-----------------------------------------------------------------------------
DENND1A.X4_BOVINE      CTATCAG-----------------------------------------------------------------------------
DENND1A.X1-4_F         ------------------------------------------------------------------------------------
DENND1A.X1-4_R         ------------------------------------------------------------------------------------

DENND1A_BOVINE         AGGCTGGACCTGGGCAGAAGTGACAGGAGCCGTGGGACACCAGGGGCCTTGAAGCTCGCCCACCCGCACAGCAGGCTCTGGAGC
DENND1A.V1_HUMAN       AGGCTGGATCTGGGCGGGAGTGAGAGGAGCCGCGGGGTGACAGTGGCCTTGAAGCTTACCCACCCGTACAACAAGCTCTGGAGC
DENND1A.X1_BOVINE      AGGCTGGACCTGGGCAGAAGTGACAGGAGCCGTGGGACACCAGGGGCCTTGAAGCTCGCCCACCCGCACAGCAGGCTCTGGAGC
DENND1A.X2_BOVINE      AGGCTGGACCTGGGCAGAAGTGACAGGAGCCGTGGGACACCAGGGGCCTTGAAGCTCGCCCACCCGCACAGCAGGCTCTGGAGC
DENND1A.X3_BOVINE      AGGCTGGACCTGGGCAGAAGTGACAGGAGCCGTGGGACACCAGGGGCCTTGAAGCTCGCCCACCCGCACAGCAGGCTCTGGAGC
DENND1A.X4_BOVINE      AGGCTGGACCTGGGCAGAAGTGACAGGAGCCGTGGGACACCAGGGGCCTTGAAGCTCGCCCACCCGCACAGCAGGCTCTGGAGC
DENND1A.X1-4_F         ------------------------------------------------------------------------------------
DENND1A.X1-4_R         ------------------------------------------------------------------------------------

DENND1A_BOVINE         CTAGGCCAGGATGACATGGCCATCCCCAGCAAGCCGCCCGCCGCCTGCCCCGAGAAGCCCTCAGCCCTGCTCGGGAACTCCCTG
DENND1A.V1_HUMAN       CTGGGCCAGGACGACATGGCCATCCCCAGCAAGCCCCCAGCTGCCTCCCCTGAGAAGCCCTCGGCCCTGCTCGGGAACTCCCTG
DENND1A.X1_BOVINE      CTAGGCCAGGATGACATGGCCATCCCCAGCAAGCCGCCCGCCGCCTGCCCCGAGAAGCCCTCAGCCCTGCTCGGGAACTCCCTG
DENND1A.X2_BOVINE      CTAGGCCAGGATGACATGGCCATCCCCAGCAAGCCGCCCGCCGCCTGCCCCGAGAAGCCCTCAGCCCTGCTCGGGAACTCCCTG
DENND1A.X3_BOVINE      CTAGGCCAGGATGACATGGCCATCCCCAGCAAGCCGCCCGCCGCCTGCCCCGAGAAGCCCTCAGCCCTGCTCGGGAACTCCCTG
DENND1A.X4_BOVINE      CTAGGCCAGGATGACATGGCCATCCCCAGCAAGCCGCCCGCCGCCTGCCCCGAG------------------------
DENND1A.X1-4_F         ------------------------------------------------------------------------------------
DENND1A.X1-4_R         ------------------------------------------------------------------------------------

DENND1A_BOVINE         GCCTCACCCTGCAGGCCCCAGAACCAGAATGGCCTCCTGAACCCCAGCGACAAGGAGGAGGTGCCCACACCCACCCTGGGCAGC
DENND1A.V1_HUMAN       GCCCTGCCTCGAAGGCCCCAGAACCGGGACAGCATCCTGAACCCCAGTGACAAGGAGGAGGTGCCCACCCCTACTCTGGGCAGC
DENND1A.X1_BOVINE      GCCTCACCCTGCAGGCCCCAGAACCAGAATGGCCTCCTGAACCCCAGCGACAAGGAGGAGGTGCCCACACCCACCCTGGGCAGC
DENND1A.X2_BOVINE      GCCTCACCCTGCAGGCCCCAGAACCAGAATGGCCTCCTGAACCCCAGCGACAAGGAGGAGGTGCCCACACCCACCCTGGGCAGC
DENND1A.X3_BOVINE      GCCTCACCCTGCAGGCCCCAGAACCAGAATGGCCTCCTGAACCCCAGCGACAAGGAGGAGGTGCCCACACCCACCCTGGGCAGC
DENND1A.X4_BOVINE      ------------------------------------------------------------------------------------
DENND1A.X1-4_F         ------------------------------------------------------------------------------------
DENND1A.X1-4_R         ------------------------------------------------------------------------------------

DENND1A_BOVINE         ATCACTATCCCCCGGCCCCAGGGCAGGAAGACCCCAGAGCTGGGCATCGTGCCCCCGCCTCCTACTGCCCGCCCAGCCAAGCTC
DENND1A.V1_HUMAN       ATCACCATCCCCCGGCCCCAAGGCAGGAAGACCCCAGAGCTGGGCATCGTGCCTCCACCGCCCATTCCCCGCCCGGCCAAGCTC
DENND1A.X1_BOVINE      ATCACTATCCCCCGGCCCCAGGGCAGGAAGACCCCAGAGCTGGGCATCGTGCCCCCGCCTCCTACTGCCCGCCCAGCCAAGCTC
DENND1A.X2_BOVINE      ATCACTATCCCCCGGCCCCAGGGCAGGAAGACCCCAGAGCTGGGCATCGTGCCCCCGCCTCCTACTGCCCGCCCAGCCAAGCTC
DENND1A.X3_BOVINE      ATCACTATCCCCCGGCCCCAGGGCAGGAAGACCCCAGAGCTGGGCATCGTGCCCCCGCCTCCTACTGCCCGCCCAGCCAAGCTC
DENND1A.X4_BOVINE      ------------------------------------------------------------------------------------
DENND1A.X1-4_F         ------------------------------------------------------------------------------------
DENND1A.X1-4_R         ------------------------------------------------------------------------------------

DENND1A_BOVINE         CAGGCCCCCGGCATCGCCCTTGGCGACTTCTTGGAACAACTGCCGGCTGAGCGGGAAAGGCGGGCTGCCCTCAGCTCAACCCCA
DENND1A.V1_HUMAN       CAGGCTGCCGGCGCCGCACTTGGTGACGTCTCAGAGCGGCTGCAGACGGATCGGGACAGGCGAGCTGCCCTGAGTCC-------
DENND1A.X1_BOVINE      CAGGCCCCCGGCATCGCCCTTGGCGACTTCTTGGAACAACTGCCGGCTGAGCGGGAAAGGCGGGCTGCCCTCAGCTCAACCCCA
DENND1A.X2_BOVINE      CAGGCCCCCGGCATCGCCCTTGGCGACTTCTTGGAACAACTGCCGGCTGAGCGGGAAAGGCGGGCTGCCCTCAGCTCAACCCCA
DENND1A.X3_BOVINE      CAGGCCCCCGGCATCGCCCTTGGCGACTTCTTGGAACAACTGCCGGCTGAGCGGGAAAGGCGGGCTGCCCTCAGCTCAACCCCA
DENND1A.X4_BOVINE      ------------------------------------------------------------------------------------
DENND1A.X1-4_F         ------------------------------------------------------------------------------------
DENND1A.X1-4_R         ------------------------------------------------------------------------------------

DENND1A_BOVINE         TTCCCTGGGCTCCTCTCCAGTGCTGCACCCCAAGACCCCACTGAACTGCTCCAGCCACTCAGCCTGGCCCCAGGGGCTGCCGGC
DENND1A.V1_HUMAN       -----AGGGCTCCTGCCTGGTGTTGTCCCCCAAGGCCCCACTGAACTGCTCCAGCCGCTCAGCCCTGGCCCCGGGGCTGCCGGC
DENND1A.X1_BOVINE      TTCCCTGGGCTCCTCTCCAGTGCTGCACCCCAAGACCCCACTGAACTGCTCCAGCCACTCAGCCTGGCCCCAGGGGCTGCCGGC
DENND1A.X2_BOVINE      TTCCCTGGGCTCCTCTCCAGTGCTGCACCCCAAGACCCCACTGAACTGCTCCAGCCACTCAGCCTGGCCCCAGGGGCTGCCGGC
DENND1A.X3_BOVINE      TTCCCTGGGCTCCTCTCCAGTGCTGCACCCCAAGACCCCACTGAACTGCTCCAGCCACTCAGCCTGGCCCCAGGGGCTGCCGGC
DENND1A.X4_BOVINE      ------------------------------------------------------------------------------------
DENND1A.X1-4_F         ------------------------------------------------------------------------------------
DENND1A.X1-4_R         ------------------------------------------------------------------------------------

DENND1A_BOVINE         AGCAGCAGTGATGCCCTGCTGGCCCTGCTGGATCCACTTAACACAACCTGGTCGGGCAGCTCCCTTCCACCAGGCCCTACAGCC
DENND1A.V1_HUMAN       ACGAGCAGTGACGCCCTGCTCGCCCTCCTGGACCCGCTCAGCACAGCCTGGTCAGGCAGCACCCTCCCGTCACGCCCCGCCACC
DENND1A.X1_BOVINE      AGCAGCAGTGATGCCCTGCTGGCCCTGCTGGATCCACTTAACACAACCTGGTCGGGCAGCTCCCTTCCACCAGGCCCTACAGCC
DENND1A.X2_BOVINE      AGCAGCAGTGATGCCCTGCTGGCCCTGCTGGATCCACTTAACACAACCTGGTCGGGCAGCTCCCTTCCACCAGGCCCTACAGCC
DENND1A.X3_BOVINE      AGCAGCAGTGATGCCCTGCTGGCCCTGCTGGATCCACTTAACACAACCTGGTCGGGCAGCTCCCTTCCACCAGGCCCTACAGCC
DENND1A.X4_BOVINE      ------------------------------------------------------------------------------------
DENND1A.X1-4_F         ------------------------------------------------------------------------------------
DENND1A.X1-4_R         ------------------------------------------------------------------------------------

DENND1A_BOVINE         CCAAATGTAGCCACCCCATTTACTCCCCAATTTAGTTTTCCCCCCATGGGGACCCCCACCCCATTTCCACAGCCATCACTCAAC
DENND1A.V1_HUMAN       CCGAATGTAGCCACCCCATTCACCCCCCAATTCAGCTTCCCCCCTGCAGGGACACCCACCCCATTCCCACAGCCACCACTCAAC
DENND1A.X1_BOVINE      CCAAATGTAGCCACCCCATTTACTCCCCAATTTAGTTTTCCCCCCATGGGGACCCCCACCCCATTTCCACAGCCATCACTCAAC
```

Exon 22

```
DENND1A.X2_BOVINE     CCAAATGTAGCCACCCCATTTACTCCCCAATTTAGTTTTCCCCCCATGGGACCCCCACCCCATTTCCACAGCCATCACTCAAC
DENND1A.X3_BOVINE     CCAAATGTAGCCACCCCATTTACTCCCCAATTTAGTTTTCCCCCCATGGGACCCCCACCCCATTTCCACAGCCATCACTCAAC
DENND1A.X4_BOVINE     -----------------------------------------------------------------------------------
DENND1A.X1-4_F        -----------------------------------------------------------------------------------
DENND1A.X1-4_R        -----------------------------------------------------------------------------------

DENND1A_BOVINE        CCCTTTGTCCCACCTCTGCCAGCGACACTGCCCACCATGCCCCTGGTCTCTGCACCAGCTGGGCCTTTTGGGGCCCCTCCTGCT
DENND1A.V1_HUMAN      CCCTTTGTCCCATCCATGCCAGCAGCCCCACCCACCCTGCCCCTGGTCTCCACACCAGCCGGGCCTTTCGGGGCCCCTCCAGCT
DENND1A.X1_BOVINE     CCCTTTGTCCCACCTCTGCCAGCGACACTGCCCACCATGCCCCTGGTCTCTGCACCAGCTGGGCCTTTTGGGGCCCCTCCTGCT
DENND1A.X2_BOVINE     CCCTTTGTCCCACCTCTGCCAGCGACACTGCCCACCATGCCCCTGGTCTCTGCACCAGCTGGGCCTTTTGGGGCCCCTCCTGCT
DENND1A.X3_BOVINE     CCCTTTGTCCCACCTCTGCCAGCGACACTGCCCACCATGCCCCTGGTCTCTGCACCAGCTGGGCCTTTTGGGGCCCCTCCTGCT
DENND1A.X4_BOVINE     -----------------------------------------------------------------------------------
DENND1A.X1-4_F        -----------------------------------------------------------------------------------
DENND1A.X1-4_R        -----------------------------------------------------------------------------------

DENND1A_BOVINE        TCCCTGGGGCCAGCCTTTGCCCCCAGCCTCCTGCTGTCCAATTCTGGCTTCTGTGCCCCACATCGATCTCAGCCCAACCTGTCT
DENND1A.V1_HUMAN      TCCCTGGGGCCGGCTTTTGCGTCCGGCCTCCTGCTGTCCAGTGCTGGCTTCTGTGCCCCTCACAGGTCTCAGCCCAACCTCTCC
DENND1A.X1_BOVINE     TCCCTGGGGCCAGCCTTTGCCCCCAGCCTCCTGCTGTCCAATTCTGGCTTCTGTGCCCCACATCGATCTCAGCCCAACCTGTCT
DENND1A.X2_BOVINE     TCCCTGGGGCCAGCCTTTGCCCCCAGCCTCCTGCTGTCCAATTCTGGCTTCTGTGCCCCACATCGATCTCAGCCCAACCTGTCT
DENND1A.X3_BOVINE     TCCCTGGGGCCAGCCTTTGCCCCCAGCCTCCTGCTGTCCAATTCTGGCTTCTGTGCCCCACATCGATCTCAGCCCAACCTGTCT
DENND1A.X4_BOVINE     -----------------------------------------------------------------------------------
DENND1A.X1-4_F        -----------------------------------------------------------------------------------
DENND1A.X1-4_R        -----------------------------------------------------------------------------------

DENND1A_BOVINE        GCCCTCTCCATGCCCAACCTCTTTGGCCAAGTGCCCATGGGTACCCAC------TCCCTGCAGCCTCTGGGTCCCCCAGCAGTC
DENND1A.V1_HUMAN      GCCCTCTCCATGCCCAACCTCTTTGGCCAGATGCCCATGGGCACCCACACGAGCCCCCTACAGCCGCTGGGTCCCCCAGCAGTT
DENND1A.X1_BOVINE     GCCCTCTCCATGCCCAACCTCTTTGGCCAAGTGCCCATGGGTACCCAC------TCCCTGCAGCCTCTGGGTCCCCCAGCAGTC
DENND1A.X2_BOVINE     GCCCTCTCCATGCCCAACCTCTTTGGCCAAGTGCCCATGGGTACCCAC------TCCCTGCAGCCTCTGGGTCCCCCAGCAGTC
DENND1A.X3_BOVINE     GCCCTCTCCATGCCCAACCTCTTTGGCCAAGTGCCCATGGGTACCCAC------TCCCTGCAGCCTCTGGGTCCCCCAGCAGTC
DENND1A.X4_BOVINE     -----------------------------------------------------------------------------------
DENND1A.X1-4_F        -----------------------------------------------------------------------------------
DENND1A.X1-4_R        -----------------------------------------------------------------------------------

DENND1A_BOVINE        GCCCCCTCAAGGATCCGAACATTGCCCCTGGCCCGCTCAAGTGCCAGGGCCGCCGAGGCCAAGCAAGGGCTGGCCCTGAGGCCT
DENND1A.V1_HUMAN      GCCCCGTCGAGGATCCGAACGTTGCCCCTGGCCCGCTCAAGTGCCAGGGCTGCTGAGACCAAGCAGGGGCTGGCCCTGAGGCCT
DENND1A.X1_BOVINE     GCCCCCTCAAGGATCCGAACATTGCCCCTGGCCCGCTCAAGTGCCAGGGCCGCCGAGGCCAAGCAAGGGCTGGCCCTGAGGCCT
DENND1A.X2_BOVINE     GCCCCCTCAAGGATCCGAACATTGCCCCTGGCCCGCTCAAGTGCCAGGGCCGCCGAGGCCAAGCAAGGGCTGGCCCTGAGGCCT
DENND1A.X3_BOVINE     GCCCCCTCAAGGATCCGAACATTGCCCCTGGCCCGCTCAAGTGCCAGGGCCGCCGAGGCCAAGCAAGGGCTGGCCCTGAGGCCT
DENND1A.X4_BOVINE     -----------------------------------------------------------------------------------
DENND1A.X1-4_F        -----------------------------------------------------------------------------------
DENND1A.X1-4_R        -----------------------------------------------------------------------------------

DENND1A_BOVINE        GGAGAACCCCCACTCCTCCCAGCCAGGCCCCCCCAGGGCCTGGAGCCAGCACTGCAGCCCTCTGCTCCACGAGAGGCCAGAGAC
DENND1A.V1_HUMAN      GGAGACCCCCCGCTTCTGCCTCCCAGGCCCCCTCAAGGCCTGGAGCCAACACTGCAGCCCTCTGCTCCTCAACAGGCCAGAGAC
DENND1A.X1_BOVINE     GGAGAACCCCCACTCCTCCCAGCCAGGCCCCCCCAGGGCCTGGAGCCAGCACTGCAGCCCTCTGCTCCACGAGAGGCCAGAGAC
DENND1A.X2_BOVINE     GGAGAACCCCCACTCCTCCCAGCCAGGCCCCCCCAGGGCCTGGAGCCAGCACTGCAGCCCTCTGCTCCACGAGAGGCCAGAGAC
DENND1A.X3_BOVINE     GGAGAACCCCCACTCCTCCCAGCCAGGCCCCCCCAGGGCCTGGAGCCAGCACTGCAGCCCTCTGCTCCACGAGAGGCCAGAGAC
DENND1A.X4_BOVINE     -----------------------------------------------------------------------------------
DENND1A.X1-4_F        -----------------------------------------------------------------------------------
DENND1A.X1-4_R        -----------------------------------------------------------------------------------

DENND1A_BOVINE        CCCTTTGAGGATTTGTTACGGAAAACCAAGCAAGATGTGAGCTCG------------GCCCCAGCCCCGGCTCCGTGGAGCAG
DENND1A.V1_HUMAN      CCCTTTGAGGATTTGTTACAGAAAACCAAGCAAGACGTGAGCCCGAGTCCGGCCCTGGCCCCGGCCCCAGACTCGGTGGAGCAG
DENND1A.X1_BOVINE     CCCTTTGAGGATTTGTTACGGAAAACCAAGCAAGATGTGAGCTCG------------GCCCCAGCCCCGGCTCCGTGGAGCAG
DENND1A.X2_BOVINE     CCCTTTGAGGATTTGTTACGGAAAACCAAGCAAGATGTGAGCTCG------------GCCCCAGCCCCGGCTCCGTGGAGCAG
DENND1A.X3_BOVINE     CCCTTTGAGGATTTGTTACGGAAAACCAAGCAAGATGTGAGCTCG------------GCCCCAGCCCCGGCTCCGTGGAGCAG
DENND1A.X4_BOVINE     -----------------------------------------------------------------------------------
DENND1A.X1-4_F        -----------------------------------------------------------------------------------
DENND1A.X1-4_R        -----------------------------------------------------------------------------------

DENND1A_BOVINE        CTCAGGAAGCAATGGGAGACCTTCGAGTGAGCAGC--GCTGAGGGTGGGGCGAG--CCAAGGCCGGCCGGCCCTGCTTCCCCTG
DENND1A.V1_HUMAN      CTCAGGAAGCAGTGGGAGACCTTCGAGTGAGCCGGGCCCTGAGGGTGGGGATGCACCGAGGCCCGAGGG---TCCGTCCACTG
DENND1A.X1_BOVINE     CTCAGGAAGCAATGGGAGACCTTCGAGTGAGCAGC--GCTGAGGGTGGGGCGAG--CCAAGGCCGGCCGGCCCTGCTTCCCCTG
DENND1A.X2_BOVINE     CTCAGGAAGCAATGGGAGACCTTCGAGTGAGCAGC--GCTGAGGGTGGGGCGAG--CCAAGGCCGGCCGGCCCTGCTTCCCCTG
DENND1A.X3_BOVINE     CTCAGGAAGCAATGGGAGACCTTCGAGTGAGCAGC--GCTGAGGGTGGGGCGAG--CCAAGGCCGGCCGGCCCTGCTTCCCCTG
DENND1A.X4_BOVINE     -----------------------------------------------------------------------------------
DENND1A.X1-4_F        -----------------------------------------------------------------------------------
DENND1A.X1-4_R        -----------------------------------------------------------------------------------

DENND1A_BOVINE        CTG-------------------------CTGTTTCTACCCAGGTTCTACTGGTGGGAAGGGATGGGAACCCTCTCTGCCGCC-CC
DENND1A.V1_HUMAN      CTGCGGTTCCGAGGCTCCCCCGCCACTCTCTCTCTGCCCAGGTTCTGCTGGTGGGAAGGGATGGGACCCCTCTCTGCTGCCCCC
```

Exon 22

```
DENND1A.X1_BOVINE     CTG-------------------------CTGTTTCTACCCAGGTTCTACTGGTGGGAAGGGATGGAACCCTCTCTGCCGCC-CC
DENND1A.X2_BOVINE     CTG-------------------------CTGTTTCTACCCAGGTTCTACTGGTGGGAAGGGATGGAACCCTCTCTGCCGCC-CC
DENND1A.X3_BOVINE     CTG-------------------------CTGTTTCTACCCAGGTTCTACTGGTGGGAAGGGATGGAACCCTCTCTGCCGCC-CC
DENND1A.X4_BOVINE     ------------------------------------------------------------------------------------
DENND1A.X1-4_F        ------------------------------------------------------------------------------------
DENND1A.X1-4_R        ------------------------------------------------------------------------------------
DENND1A_BOVINE        TCCTCCTTTCCACACTGCCCATCTCT-------------GGAGAATGGCGCCAGTTCCAGCCTGGGAATCGACCCAGCTCCTGG
DENND1A.V1_HUMAN      TCCTCCCCTCCACACTGCCCATCTCTGATGTCTGGCCCTGGGGAATGGCACCAGTTCCAGCCTGGGAATCAACCCAGTTCCTGA
DENND1A.X1_BOVINE     TCCTCCTTTCCACACTGCCCATCTCT-------------GGAGAATGGCGCCAGTTCCAGCCTGGGAATCGACCCAGCTCCTGG
DENND1A.X2_BOVINE     TCCTCCTTTCCACACTGCCCATCTCT-------------GGAGAATGGCGCCAGTTCCAGCCTGGGAATCGACCCAGCTCCTGG
DENND1A.X3_BOVINE     TCCTCCTTTCCACACTGCCCATCTCT-------------GGAGAATGGCGCCAGTTCCAGCCTGGGAATCGACCCAGCTCCTGG
DENND1A.X4_BOVINE     ------------------------------------------------------------------------------------
DENND1A.X1-4_F        ------------------------------------------------------------------------------------
DENND1A.X1-4_R        ------------------------------------------------------------------------------------

DENND1A_BOVINE        GTGCCTGTCCTGTCCCGACCC-----TTCCCCTGCCCCCCTAGTTGGGTTTTGCACTAAAGAGGTCAGCTGGGCCAGCG--ATT
DENND1A.V1_HUMAN      GTGCCCATCCCACCCCGCGGTTGCCTCTCCTCGGCACCCTTGATTGGGTTTTGCACTAAAGAGGTCAGCTGGGCCAATGATATT
DENND1A.X1_BOVINE     GTGCCTGTCCTGTCCCGACCC-----TTCCCCTGCCCCCCTAGTTGGGTTTTGCACTAAAGAGGTCAGCTGGGCCAGCG--ATT
DENND1A.X2_BOVINE     GTGCCTGTCCTGTCCCGACCC-----TTCCCCTGCCCCCCTAGTTGGGTTTTGCACTAAAGAGGTCAGCTGGGCCAGCG--ATT
DENND1A.X3_BOVINE     GTGCCTGTCCTGTCCCGACCC-----TTCCCCTGCCCCCCTAGTTGGGTTTTGCACTAAAGAGGTCAGCTGGGCCAGCG--ATT
DENND1A.X4_BOVINE     ------------------------------------------------------------------------------------
DENND1A.X1-4_F        ------------------------------------------------------------------------------------
DENND1A.X1-4_R        ------------------------------------------------------------------------------------

DENND1A_BOVINE        GCCCCAGGCCACATCTTACCCACCTTCCCTCTGGAAACTGCCCACCAGGAGCCCCGCGCGTCCTCAGGATGTCTC-CTCCTGAG
DENND1A.V1_HUMAN      GCTCCAGACCGAGTCCTACCCACCTTCCCCCGGAAG-TGTCCCAAGAGGCTCC-------------GAAGGCCTCCCCTCCGAG
DENND1A.X1_BOVINE     GCCCCAGGCCACATCTTACCCACCTTCCCTCTGGAAACTGCCCACCAGGAGCCCCGCGCGTCCTCAGGATGTCTC-CTCCTGAG
DENND1A.X2_BOVINE     GCCCCAGGCCACATCTTACCCACCTTCCCTCTGGAAACTGCCCACCAGGAGCCCCGCGCGTCCTCAGGATGTCTC-CTCCTGAG
DENND1A.X3_BOVINE     GCCCCAGGCCACATCTTACCCACCTTCCCTCTGGAAACTGCCCACCAGGAGCCCCGCGCGTCCTCAGGATGTCTC-CTCCTGAG
DENND1A.X4_BOVINE     ------------------------------------------------------------------------------------
DENND1A.X1-4_F        ------------------------------------------------------------------------------------
DENND1A.X1-4_R        ------------------------------------------------------------------------------------

DENND1A_BOVINE        CCCCGTTCTCTTGCCCCAGCTGCAGCCCAGCCCGCACACCCCACCTTGATGGGCACGAGTGTTGGCGGTACA--------CCAG
DENND1A.V1_HUMAN      CCCAGCTCTCCTGTCTCCTCCACAGCCAGGCCCTGCACGCCCACCTCCTCGGACACAGGTGACAGGGTTACCCTCCAGTTTGAG
DENND1A.X1_BOVINE     CCCCGTTCTCTTGCCCCAGCTGCAGCCCAGCCCGCACACCCCACCTTGATGGGCACGAGTGTTGGCGGTACA--------CCAG
DENND1A.X2_BOVINE     CCCCGTTCTCTTGCCCCAGCTGCAGCCCAGCCCGCACACCCCACCTTGATGGGCACGAGTGTTGGCGGTACA--------CCAG
DENND1A.X3_BOVINE     CCCCGTTCTCTTGCCCCAGCTGCAGCCCAGCCCGCACACCCCACCTTGATGGGCACGAGTGTTGGCGGTACA--------CCAG
DENND1A.X4_BOVINE     ------------------------------------------------------------------------------------
DENND1A.X1-4_F        ------------------------------------------------------------------------------------
DENND1A.X1-4_R        ------------------------------------------------------------------------------------

DENND1A_BOVINE        CTCGTCTGCACGAGAGGCACGTGGTTTGGAGTTTGAAGTAAGGAACCCCCCTCCCCCACCCCTCCCCAGCTGGAGGATCTACCT
DENND1A.V1_HUMAN      CTCATCTGCACGAGACACAGGTAGCTTGGGGT-TGAAGTTAGGACT--------------CCTCCTGGGCTGGAGGATTTACCT
DENND1A.X1_BOVINE     CTCGTCTGCACGAGAGGCACGTGGTTTGGAGTTTGAAGTAAGGAACCCCCCTCCCCCACCCCTCCCCAGCTGGAGGATCTACCT
DENND1A.X2_BOVINE     CTCGTCTGCACGAGAGGCACGTGGTTTGGAGTTTGAAGTAAGGAACCCCCCTCCCCCACCCCTCCCCAGCTGGAGGATCTACCT
DENND1A.X3_BOVINE     CTCGTCTGCACGAGAGGCACGTGGTTTGGAGTTTGAAGTAAGGAACCCCCCTCCCCCACCCCTCCCCAGCTGGAGGATCTACCT
DENND1A.X4_BOVINE     ------------------------------------------------------------------------------------
DENND1A.X1-4_F        ------------------------------------------------------------------------------------
DENND1A.X1-4_R        ------------------------------------------------------------------------------------

DENND1A_BOVINE        GGCGAGGCATTTCCAAACCCTGTCTAGCAATATGCACACTCTTTCTTTACTGAGTCTTCACCCCAACCCCTCCACCTCCG-TTT
DENND1A.V1_HUMAN      GGTGGGGCACTTCCAGACTGTTTCTAGCAATATACACACACGTTCTTTCCTGTGTCTTCACCCCAAAACTTC------AGTTGA
DENND1A.X1_BOVINE     GGCGAGGCATTTCCAAACCCTGTCTAGCAATATGCACACTCTTTCTTTACTGAGTCTTCACCCCAACCCCTCCACCTCCG-TTT
DENND1A.X2_BOVINE     GGCGAGGCATTTCCAAACCCTGTCTAGCAATATGCACACTCTTTCTTTACTGAGTCTTCACCCCAACCCCTCCACCTCCG-TTT
DENND1A.X3_BOVINE     GGCGAGGCATTTCCAAACCCTGTCTAGCAATATGCACACTCTTTCTTTACTGAGTCTTCACCCCAACCCCTCCACCTCCG-TTT
DENND1A.X4_BOVINE     ------------------------------------------------------------------------------------
DENND1A.X1-4_F        ------------------------------------------------------------------------------------
DENND1A.X1-4_R        ------------------------------------------------------------------------------------

DENND1A_BOVINE        TTCTGACTTGGTTGGGATCTGGGGGC----------------------------------------------------------
DENND1A.V1_HUMAN      TTCTGACCTGGG-AGGATCTGGGGACCAGGGGG-TCTTGGGCTGCCTTGTGATACAC------AGCCCCAGCCA-CCCTGCATG
DENND1A.X1_BOVINE     TTCTGACTTGGTTGGGATCTGGGGGCTGAGGGATCCACGGGATCCCTTGTGGTAACCAAACCAAGCCCCAGCCAGCCCTGCATG
DENND1A.X2_BOVINE     TTCTGACTTGGTTGGGATCTGGGGGCTGAGGGATCCACGGGATCCCTTGTGGTAACCAAACCAAGCCCCAGCCAGCCCTGCATG
DENND1A.X3_BOVINE     TTCTGACTTGGTTGGGATCTGGGGGCTGAGGGATCCACGGGATCCCTTGTGGTAACCAAACCAAGCCCCAGCCAGCCCTGCATG
DENND1A.X4_BOVINE     ------------------------------------------------------------------------------------
DENND1A.X1-4_F        ------------------------------------------------------------------------------------
DENND1A.X1-4_R        ------------------------------------------------------------------------------------

DENND1A_BOVINE        ------------------------------------------------------------------------------------
DENND1A.V1_HUMAN      GGGGCTGCGAGCACCAGCAACTTTGATTTATAGAAGGAAA-ATGGAAACCCC--CATCTGAGTATTTTGGGAGGAGCCCCCAGC
```

Exon 22

```
DENND1A.X1_BOVINE      AGGGCCGGGAGCACCAGCAACTTTGATT-ATAGAAGACAAGACGGCAACAGGCAAACCCAAGAGCATTGGGACGGGTCCG--GC
DENND1A.X2_BOVINE      AGGGCCGGGAGCACCAGCAACTTTGATT-ATAGAAGACAAGACGGCAACAGGCAAACCCAAGAGCATTGGGACGGGTCCG--GC
DENND1A.X3_BOVINE      AGGGCCGGGAGCACCAGCAACTTTGATT-ATAGAAGACAAGACGGCAACAGGCAAACCCAAGAGCATTGGGACGGGTCCG--GC
DENND1A.X4_BOVINE      ------------------------------------------------------------------------------------
DENND1A.X1-4_F         ------------------------------------------------------------------------------------
DENND1A.X1-4_R         ------------------------------------------------------------------------------------
DENND1A_BOVINE         ------------------------------------------------------------------------------------
DENND1A.V1_HUMAN       CCTCATCCAGCTCTGGCACGCTGATACCTCCAGGTACTCCC--------CTCACTGTCAAAGCTGGGGCTCAGCCTCTTGTCAT
DENND1A.X1_BOVINE      CGCTGTCCA-CACTCTCCCTTGGGTCCCTAGAGGTTCTCCTGGTCTCCAGTTATGATC--ACATGAGGCTCAGACTCTTGTCGA
DENND1A.X2_BOVINE      CGCTGTCCA-CACTCTCCCTTGGGTCCCTAGAGGTTCTCCTGGTCTCCAGTTATGATC--ACATGAGGCTCAGACTCTTGTCGA
DENND1A.X3_BOVINE      CGCTGTCCA-CACTCTCCCTTGGGTCCCTAGAGGTTCTCCTGGTCTCCAGTTATGATC--ACATGAGGCTCAGACTCTTGTCGA
DENND1A.X4_BOVINE      ------------------------------------------------------------------------------------
DENND1A.X1-4_F         ------------------------------------------------------------------------------------
DENND1A.X1-4_R         ------------------------------------------------------------------------------------

DENND1A_BOVINE         ------------------------------------------------------------------------------------
DENND1A.V1_HUMAN       CTGGAGCTTTGTGGGCAAAGCTGAGAAGCTGCAACCCAGATTTCAACCCAAAAAGGTCAAGCTGAATGCCTCAGACTGATGTGG
DENND1A.X1_BOVINE      GTGGGTCTCAGGGGGCAGAGCTGAGAAGCAGACACCCAGACCCCCACT--GGAAGGCCAAGCTTACCACCACAGA----GATGC
DENND1A.X2_BOVINE      GTGGGTCTCAGGGGGCAGAGCTGAGAAGCAGACACCCAGACCCCCACT--GGAAGGCCAAGCTTACCACCACAGA----GATGC
DENND1A.X3_BOVINE      GTGGGTCTCAGGGGGCAGAGCTGAGAAGCAGACACCCAGACCCCCACT--GGAAGGCCAAGCTTACCACCACAGA----GATGC
DENND1A.X4_BOVINE      ------------------------------------------------------------------------------------
DENND1A.X1-4_F         ------------------------------------------------------------------------------------
DENND1A.X1-4_R         ------------------------------------------------------------------------------------

DENND1A_BOVINE         ------------------------------------------------------------------------------------
DENND1A.V1_HUMAN       AAGGCAG------CTGGCCTTCCTGGGTTGGAACGAGGCAGTGGCCCTGAGCCCCTTCTCCAGGGCCAGGTAGAAAGGACAAAC
DENND1A.X1_BOVINE      AAGGGTCCCAGTGCCTGGCTCCTTGGGGCAGGAGGGGGCAG-GGCACTAAGCC-CCTCTCCAGGCCCCTGGTGAGTGAGCAGGC
DENND1A.X2_BOVINE      AAGGGTCCCAGTGCCTGGCTCCTTGGGGCAGGAGGGGGCAG-GGCACTAAGCC-CCTCTCCAGGCCCCTGGTGAGTGAGCAGGC
DENND1A.X3_BOVINE      AAGGGTCCCAGTGCCTGGCTCCTTGGGGCAGGAGGGGGCAG-GGCACTAAGCC-CCTCTCCAGGCCCCTGGTGAGTGAGCAGGC
DENND1A.X4_BOVINE      ------------------------------------------------------------------------------------
DENND1A.X1-4_F         ------------------------------------------------------------------------------------
DENND1A.X1-4_R         ------------------------------------------------------------------------------------

DENND1A_BOVINE         ------------------------------------------------------------------------------------
DENND1A.V1_HUMAN       TTGGTCTCTGCCTCG-GGGAAGCAGGAGGAGGGCTAGAAGCCAGTCCCTCCCCACCTGCCCAGAGCTCCAGGCCAGCACAGAAA
DENND1A.X1_BOVINE      TTGGCTTCTGGCAGCCTGGAG---GCAGGAGGGCTTGGAGTCACCCCCTCCCTGCCCACCTTGTGATTCCTGCCAGCATAGAAG
DENND1A.X2_BOVINE      TTGGCTTCTGGCAGCCTGGAG---GCAGGAGGGCTTGGAGTCACCCCCTCCCTGCCCACCTTGTGATTCCTGCCAGCATAGAAG
DENND1A.X3_BOVINE      TTGGCTTCTGGCAGCCTGGAG---GCAGGAGGGCTTGGAGTCACCCCCTCCCTGCCCACCTTGTGATTCCTGCCAGCATAGAAG
DENND1A.X4_BOVINE      ------------------------------------------------------------------------------------
DENND1A.X1-4_F         ------------------------------------------------------------------------------------
DENND1A.X1-4_R         ------------------------------------------------------------------------------------

DENND1A_BOVINE         ------------------------------------------------------------------------------------
DENND1A.V1_HUMAN       TTCCTGAGGCCAACGTCACCAAAGTTAGATTGAAT-GTT-------------TATTATCTTTCTTTTTCCTTTTTACCTTATTG
DENND1A.X1_BOVINE      TTCCTGAGGCCAACATCACCAAAGCTGAATTGAAATGTTTTGTTATTATTTCTTTTATCTTTCTTTTCCTTTTTACTTTTATTG
DENND1A.X2_BOVINE      TTCCTGAGGCCAACATCACCAAAGCTGAATTGAAATGTTTTGTTATTATTTCTTTTATCTTTCTTTTCCTTTTTACTTTTATTG
DENND1A.X3_BOVINE      TTCCTGAGGCCAACATCACCAAAGCTGAATTGAAATGTTTTGTTATTATTTCTTTTATCTTTCTTTTCCTTTTTACTTTTATTG
DENND1A.X4_BOVINE      ------------------------------------------------------------------------------------
DENND1A.X1-4_F         ------------------------------------------------------------------------------------
DENND1A.X1-4_R         ------------------------------------------------------------------------------------

DENND1A_BOVINE         ------------------------------------------------------------------------------------
DENND1A.V1_HUMAN       ATTTGATG-AATCTTGAAATGGATTCATTTCCATAAACCAAGTTAAAGTATGGCCCGACCATTTAAGAAAACAACCATCTGAGA
DENND1A.X1_BOVINE      ATTTGATGGAATCTTGAAATTGACTAGTTTCAATAAACCAAGTTAAAATATGGCCCAACCATTTAAGAAAACAACCATCTGAGA
DENND1A.X2_BOVINE      ATTTGATGGAATCTTGAAATTGACTAGTTTCAATAAACCAAGTTAAAATATGGCCCAACCATTTAAGAAAACAACCATCTGAGA
DENND1A.X3_BOVINE      ATTTGATGGAATCTTGAAATTGACTAGTTTCAATAAACCAAGTTAAAATATGGCCCAACCATTTAAGAAAACAACCATCTGAGA
DENND1A.X4_BOVINE      ------------------------------------------------------------------------------------
DENND1A.X1-4_F         ------------------------------------------------------------------------------------
DENND1A.X1-4_R         ------------------------------------------------------------------------------------

DENND1A_BOVINE         ------------------------------------------------------------------------------------
DENND1A.V1_HUMAN       CACGCAGGAAATTGTGAGCATTTCGACCCGAGCTCTCATTTCCTATTTGTGAAGGGTCAGACACAGTCTACCCAGGGGTGTCTG
DENND1A.X1_BOVINE      CATGCAGGAAATTGTGAACATTTTGACCTGATTTCTCATTTCCTATTTGTGAATGGTCAGACACACAGT-CTCAGGGGTGTCTG
DENND1A.X2_BOVINE      CATGCAGGAAATTGTGAACATTTTGACCTGATTTCTCATTTCCTATTTGTGAATGGTCAGACACACAGT-CTCAGGGGTGTCTG
DENND1A.X3_BOVINE      CATGCAGGAAATTGTGAACATTTTGACCTGATTTCTCATTTCCTATTTGTGAATGGTCAGACACACAGT-CTCAGGGGTGTCTG
DENND1A.X4_BOVINE      ------------------------------------------------------------------------------------
DENND1A.X1-4_F         ------------------------------------------------------------------------------------
DENND1A.X1-4_R         ------------------------------------------------------------------------------------

DENND1A_BOVINE         ------------------------------------------------------------------------------------
DENND1A.V1_HUMAN       GG-GGACAAGGGGGTCTCTGGAGATG----TC-ACCCAGGGAGCCCCCTCTATGTCTGAGAGGCTGCCACTGCTGCAC-A-TGC
```

Exon 22

```
DENND1A.X1_BOVINE      AGGGGGCAAGGGGGTTTCTAGAGGCCCCTATAACCCCAGGGAGCCTTGTCATCATCTGAGGGACTGGGAGATCACCCCCACCCG
DENND1A.X2_BOVINE      AGGGGGCAAGGGGGTTTCTAGAGGCCCCTATAACCCCAGGGAGCCTTGTCATCATCTGAGGGACTGGGAGATCACCCCCACCCG
DENND1A.X3_BOVINE      AGGGGGCAAGGGGGTTTCTAGAGGCCCCTATAACCCCAGGGAGCCTTGTCATCATCTGAGGGACTGGGAGATCACCCCCACCCG
DENND1A.X4_BOVINE      ------------------------------------------------------------------------------------
DENND1A.X1-4_F         ------------------------------------------------------------------------------------
DENND1A.X1-4_R         ------------------------------------------------------------------------------------

DENND1A_BOVINE         ------------------------------------------------------------------------------------
DENND1A.V1_HUMAN       TCAGT-----------------GAGGCTTGGC-------------------GGCCATCCTGG-----CACATGGCTCTT-----
DENND1A.X1_BOVINE      TGAGTGAGCGAGGCCCGACCCTGAGGTTTGCTCCAACAGCATGGGGTGTGAAGGGCTGCTGGCCCCACACGAGCACCTTCGTCA
DENND1A.X2_BOVINE      TGAGTGAGCGAGGCCCGACCCTGAGGTTTGCTCCAACAGCATGGGGTGTGAAGGGCTGCTGGCCCCACACGAGCACCTTCGTCA
DENND1A.X3_BOVINE      TGAGTGAGCGAGGCCCGACCCTGAGGTTTGCTCCAACAGCATGGGGTGTGAAGGGCTGCTGGCCCCACACGAGCACCTTCGTCA
DENND1A.X4_BOVINE      ------------------------------------------------------------------------------------
DENND1A.X1-4_F         ------------------------------------------------------------------------------------
DENND1A.X1-4_R         ------------------------------------------------------------------------------------

DENND1A_BOVINE         ------------------------------------------------------------------------------------
DENND1A.V1_HUMAN       ---------CCTGGGTCAACCGTGACCTGTCTGGCTCAGGAATGGGCTCTGGCTGCTGG-----GGGAGCCGTGTCACTCCTGG
DENND1A.X1_BOVINE      GTCCCGGCCCCTGGGGCAAGTCCTCTCTTTCAGTCT-CGCAATGTCCTCTGGCCACCGGAAGCAGGGCGCTGTGTCACGCCTGG
DENND1A.X2_BOVINE      GTCCCGGCCCCTGGGGCAAGTCCTCTCTTTCAGTCT-CGCAATGTCCTCTGGCCACCGGAAGCAGGGCGCTGTGTCACGCCTGG
DENND1A.X3_BOVINE      GTCCCGGCCCCTGGGGCAAGTCCTCTCTTTCAGTCT-CGCAATGTCCTCTGGCCACCGGAAGCAGGGCGCTGTGTCACGCCTGG
DENND1A.X4_BOVINE      ------------------------------------------------------------------------------------
DENND1A.X1-4_F         ------------------------------------------------------------------------------------
DENND1A.X1-4_R         ------------------------------------------------------------------------------------

DENND1A_BOVINE         ------------------------------------------------------------------------------------
DENND1A.V1_HUMAN       GCCATGGGGGCACCTCCTGGGCACTTAGGTGTTTCAGCATAGATTCCAGTTTCGCACCCTGGGCAGACCCCCAGGCCCCATCCG
DENND1A.X1_BOVINE      GCCACACGGGTGCCTCTAGGACACTCAGGTGTTTCAGCAGAGATTGCAGATC--CATCCTGGGCAGACCCCCGAGCCCTGTCCT
DENND1A.X2_BOVINE      GCCACACGGGTGCCTCTAGGACACTCAGGTGTTTCAGCAGAGATTGCAGATC--CATCCTGGGCAGACCCCCGAGCCCTGTCCT
DENND1A.X3_BOVINE      GCCACACGGGTGCCTCTAGGACACTCAGGTGTTTCAGCAGAGATTGCAGATC--CATCCTGGGCAGACCCCCGAGCCCTGTCCT
DENND1A.X4_BOVINE      ------------------------------------------------------------------------------------
DENND1A.X1-4_F         ------------------------------------------------------------------------------------
DENND1A.X1-4_R         ------------------------------------------------------------------------------------

DENND1A_BOVINE         ------------------------------------------------------------------------------------
DENND1A.V1_HUMAN       GGATA-GGGCAG-----------AGGAGGTGCTG-GCGGCCCCAGGGAAGGAGGGTGTGTACCCCAAGGC-CCCCTGGCTGTGC
DENND1A.X1_BOVINE      GACGGGGGGCAGAGTCTTAAGGCAAGAGGTGCTCGGTGGCCTCAGGGAAGCCGAG--CCCACCCCATGGTCTCCCTGGCTGGGG
DENND1A.X2_BOVINE      GACGGGGGGCAGAGTCTTAAGGCAAGAGGTGCTCGGTGGCCTCAGGGAAGCCGAG--CCCACCCCATGGTCTCCCTGGCTGGGG
DENND1A.X3_BOVINE      GACGGGGGGCAGAGTCTTAAGGCAAGAGGTGCTCGGTGGCCTCAGGGAAGCCGAG--CCCACCCCATGGTCTCCCTGGCTGGGG
DENND1A.X4_BOVINE      ------------------------------------------------------------------------------------
DENND1A.X1-4_F         ------------------------------------------------------------------------------------
DENND1A.X1-4_R         ------------------------------------------------------------------------------------

DENND1A_BOVINE         ------------------------------------------------------------------------------------
DENND1A.V1_HUMAN       TGAGGGGCTGGGGTGAGCGCTCCATGTTCACATGAGCACTGCTGCCTCTTCACTTGTGGGACTT--TTTGCAAACCCAAGGATG
DENND1A.X1_BOVINE      ------CCAGTGGCAAGAGCACCATGTTCACAAGAGCACTGCTGTCTCTTCCCTTGCTGGACTGAGTCTGCAAATCCAAGGATG
DENND1A.X2_BOVINE      ------CCAGTGGCAAGAGCACCATGTTCACAAGAGCACTGCTGTCTCTTCCCTTGCTGGACTGAGTCTGCAAATCCAAGGATG
DENND1A.X3_BOVINE      ------CCAGTGGCAAGAGCACCATGTTCACAAGAGCACTGCTGTCTCTTCCCTTGCTGGACTGAGTCTGCAAATCCAAGGATG
DENND1A.X4_BOVINE      ------------------------------------------------------------------------------------
DENND1A.X1-4_F         ------------------------------------------------------------------------------------
DENND1A.X1-4_R         ------------------------------------------------------------------------------------

DENND1A_BOVINE         ------------------------------------------
DENND1A.V1_HUMAN       AACTTTGT--GTGCATTCAATAAAATCATCTTGGGGAAGAGG
DENND1A.X1_BOVINE      AATTTTGTGTGTGCGTTCAATAAAATCTTCTTGGAGAGAAA-
DENND1A.X2_BOVINE      AATTTTGTGTGTGCGTTCAATAAAATCTTCTTGGAGAGAAA-
DENND1A.X3_BOVINE      AATTTTGTGTGTGCGTTCAATAAAATCTTCTTGGAGAGAAA-
DENND1A.X4_BOVINE      ------------------------------------------
DENND1A.X1-4_F         ------------------------------------------
DENND1A.X1-4_R         ------------------------------------------
```

Exon 22
