# Supplementary material for: Could perturbed fetal development of the ovary contribute to the development of polycystic ovary syndrome in later life?
Source: PLoS One. 2020 Feb 20;15(2):e0229351. doi: 10.1371/journal.pone.0229351 (PMC7032716; doi:10.1371/journal.pone.0229351)
Supplement: S3 Fig — (PDF) [file pone.0229351.s003.pdf]

Fig S3. Alignment of the exons of human *DENND1A V1, V3* and *V4* and *DENND1A.V1,3,4* primer sequences. Alignment was generated with CLUSTAL O (1.2.4) multiple sequence alignment based on sequences available at Ensembl Asia with transcript ID: human *DENND1A.V1* (ENST00000373624.6) and at NCBI PubMed with accession numbers: *DENND1A.V3* (NM_001352964) and *DENND1A.V4* (NM_001352965). The alignment was analysed with T-COFFEE (1) and colored based the consistency: red (high), yellow (average); green and blue (poor). Consistency is estimated from CORE index. Aqua highlighted areas represent the primer sequences. .... represent intronic areas not shown so as to reduce the size of this file.

1. Notredame C, Higgins DG, Heringa J. T-Coffee: A novel method for fast and accurate multiple sequence alignment. J Mol Biol. 2000;302(1):205-17. doi: 10.1006/jmbi.2000.4042.

Exon 1

```
DENND1A.V1         CGCGCGCCGGGCACGCGCGCCGGCGACCATGGCGTTCGCCGGGCTGGAGCGAGTACATTAACCCCTGGAGGCGGCGGCGGCGGCGAGGGAGCG
DENND1A.V3         CGCGCGCCGGGCACGCGCGCCGGCGACCATGGCGTTCGCCGGGCTGGAGCGAGTACATTAACCCCTGGAGGCGGCGGCGGCGGCGAGGGAGCG
DENND1A.V4         CGCGCGCCGGGCACGCGCGCCGGCGACCATGGCGTTCGCCGGGCTGGAGCGAGTACATTAACCCCTGGAGGCGGCGGCGGCGGCGAGGGAGCG
DENND1A.V1,3,4_F   ---------------------------------------------------------------------------------------------
DENND1A.V1,3,4_R   ---------------------------------------------------------------------------------------------

DENND1A.V1         AGCCTCGAGCGGGCGGGCCCCAGCCTGAGGGAAGGGAGGAAGGGGCGGGGAGAGCGCCAGAGGGAGGCCGGTCGGCCGCGGGCGGGCGGGCAG
DENND1A.V3         AGCCTCGAGCGGGCGGGCCCCAGCCTGAGGGAAGGGAGGAAGGGGCGGGGAGAGCGCCAGAGGGAGGCCGGTCGGCCGCGGGCGGGCGGGCAG
DENND1A.V4         AGCCTCGAGCGGGCGGGCCCCAGCCTGAGGGAAGGGAGGAAGGGGCGGGGAGAGCGCCAGAGGGAGGCCGGTCGGCCGCGGGCGGGCGGGCAG
DENND1A.V1,3,4_F   ---------------------------------------------------------------------------------------------
DENND1A.V1,3,4_R   ---------------------------------------------------------------------------------------------

DENND1A.V1         CGCAGCGCCGAGCGGGGCCCGCGGGCCCATGAGGAGGCCTGGGGACCATGGGCTCCAGGATCAAgtgagtgcggcc.........gtttcttttgtag
DENND1A.V3         CGCAGCGCCGAGCGGGGCCCGCGGGCCCATGAGGAGGCCTGGGGACCATGGGCTCCAGGATCAA
DENND1A.V4         CGCAGCGCCGAGCGGGGCCCGCGGGCCCATGAGGAGGCCTGGGGACCATGGGCTCCAGGATCAA-----------------------------
DENND1A.V1,3,4_F   ---------------------------------------------------------------------------------------------
DENND1A.V1,3,4_R   ---------------------------------------------------------------------------------------------
```

Exon 2

```
DENND1A.V1         GCAGAATCCAGAGACCACATTTGAAGTATATGTTGAAGTGGCCTATCCCAGGACAGGTGGCACTCTTTCAGgtacttt...............ttatttacag
DENND1A.V3         GCAGAATCCAGAGACCACATTTGAAGTATATGTTGAAGTGGCCTATCCCAGGACAGGTGGCACTCTTTCAG
DENND1A.V4         ---------------------------------------------------------------------------------------------
DENND1A.V1,3,4_F   ---------------------------------------------------------------------------------------------
DENND1A.V1,3,4_R   ---------------------------------------------------------------------------------------------
```

Exon 3

```
DENND1A.V1         ATCCTGAGGTGCAGAGGCAATTCCCGGAGGACTACAGTGACCAGgttcggaatgcgt..........................................................tctttctctccacag
DENND1A.V3         ATCCTGAGGTGCAGAGGCAATTCCCGGAGGACTACAGTGACCAG-------------------------------------------------
DENND1A.V4         ---------------------------------------------------------------------------------------------
DENND1A.V1,3,4_F   ---------------------------------------------------------------------------------------------
DENND1A.V1,3,4_R   ---------------------------------------------------------------------------------------------
```

Exon 4

```
DENND1A.V1         GAAGTATCTACAGACTTTGACCAAGTTTTGTTTCCCCTTCTATGTGGACAGgtagtgtcagattttcaaa............gtagtgtcagattttcaaa
DENND1A.V3         GAAGTATCTACAGACTTTGACCAAGTTTTGTTTCCCCTTCTATGTGGACAG------------------------------------------
DENND1A.V4         GAAGTATCTACAGACTTTGACCAAGTTTTGTTTCCCCTTCTATGTGGACAG------------------------------------------
DENND1A.V1,3,4_F   ---------------------------------------------------------------------------------------------
DENND1A.V1,3,4_R   ---------------------------------------------------------------------------------------------
```

Exon 5

```
DENND1A.V1         CCTCACAGTTAGCCAAGTTGGCCAGAACTTCACATTCGTGCTCACTGCATTGACAGCAAACAGAGATTCGGGTTCTGCCGCTTATCTTCAGGA
DENND1A.V3         CCTCACAGTTAGCCAAGTTGGCCAGAACTTCACATTCGTGCTCACTGCATTGACAGCAAACAGAGATTCGGGTTCTGCCGCTTATCTTCAGGA
DENND1A.V4         CCTCACAGTTAGCCAAGTTGGCCAGAACTTCACATTCGTGCTCACTGCATTGACAGCAAACAGAGATTCGGGTTCTGCCGCTTATCTTCAGGA
DENND1A.V1,3,4_F   ---------------------------------------------------------------------------------------------
DENND1A.V1,3,4_R   ---------------------------------------------------------------------------------------------

DENND1A.V1         GCGAAGAGCTGCTTCTGTATCTTAAGgtaagggagaaggcttgggctgt.........................................................................tttcctcttcttcctccag
DENND1A.V3         GCGAAGAGCTGCTTCTGTATCTTAAG-------------------------------------------------------------------
DENND1A.V4         GCGAAGAGCTGCTTCTGTATCTTAAG-------------------------------------------------------------------
DENND1A.V1,3,4_F   ---------------------------------------------------------------------------------------------
DENND1A.V1,3,4_R   ---------------------------------------------------------------------------------------------
```

Exon 6

```
DENND1A.V1         CTATCTCCCCTGGTTCGAGGTATTTTATAAGCTGCTTAACATCCTGGCAGATTACACGACAAAAAGACAGgtatttacc......... tttctttcag
DENND1A.V3         CTATCTCCCCTGGTTCGAGGTATTTTATAAGCTGCTTAACATCCTGGCAGATTACACGACAAAAAGACAG-----------------------
DENND1A.V4         CTATCTCCCCTGGTTCGAGGTATTTTATAAGCTGCTTAACATCCTGGCAGATTACACGACAAAAAGACAG-----------------------
DENND1A.V1,3,4_F   ---------------------------------------------------------------------------------------------
DENND1A.V1,3,4_R   ---------------------------------------------------------------------------------------------
```

Exon 7

```
DENND1A.V1         GAAAATCAGTGGAATGAGCTTCTTGAAACTCTGCACAAACTTCCCATCCCTGACCCAGGAGTGTCTGTCCATCTCAGCGTGgtaa......ttctag
DENND1A.V3         GAAAATCAGTGGAATGAGCTTCTTGAAACTCTGCACAAACTTCCCATCCCTGACCCAGGAGTGTCTGTCCATCTCAGCGTG------------
DENND1A.V4         GAAAATCAGTGGAATGAGCTTCTTGAAACTCTGCACAAACTTCCCATCCCTGACCCAGGAGTGTCTGTCCATCTCAGCGTG------------
```

```
DENND1A.V1,3,4_F   ---------------------------------------------------------------------------------------------
DENND1A.V1,3,4_R   ---------------------------------------------------------------------------------------------

DENND1A.V1         CATTCTTATTTTACTGTGCCTGATACCAGAGAACTTCCCAGCATACCTGAGAATgtaagtacttggg..........cttctttctcctag
DENND1A.V3         CATTCTTATTTTACTGTGCCTGATACCAGAGAACTTCCCAGCATACCTGAGAAT---------------------------------------
DENND1A.V4         CATTCTTATTTTACTGTGCCTGATACCAGAGAACTTCCCAGCATACCTGAGAAT---------------------------------------
DENND1A.V1,3,4_F   ---------------------------------------------------------------------------------------------
DENND1A.V1,3,4_R   ---------------------------------------------------------------------------------------------   Exon 8

DENND1A.V1         AGAAATCTGACAGAATATTTTGTGGCTGTGGATGTTAACAACATGTTGCATCTGTACGCCAGTATGCTGTACGAACGCCGGATACTCATCATT
DENND1A.V3         AGAAATCTGACAGAATATTTTGTGGCTGTGGATGTTAACAACATGTTGCATCTGTACGCCAGTATGCTGTACGAACGCCGGATACTCATCATT
DENND1A.V4         AGAAATCTGACAGAATATTTTGTGGCTGTGGATGTTAACAACATGTTGCATCTGTACGCCAGTATGCTGTACGAACGCCGGATACTCATCATT
DENND1A.V1,3,4_F   ---------------------------------------------------------------------------------------------
DENND1A.V1,3,4_R   ---------------------------------------------------------------------------------------------

DENND1A.V1         TGCAGCAAACTCAGCACTgtgagtagacagtcttaagact..........................ctctgctttgctctgttccag
DENND1A.V3         TGCAGCAAACTCAGCACT---------------------------------------------------------------------------
DENND1A.V4         TGCAGCAAACTCAGCACT---------------------------------------------------------------------------
DENND1A.V1,3,4_F   ---------------------------------------------------------------------------------------------
DENND1A.V1,3,4_R   ---------------------------------------------------------------------------------------------   Exon 9

DENND1A.V1         CTGACTGCCTGCATCCACGGGTCTGCGGCGATGCTCTACCCCATGTACTGGCAGCACGTGTACATCCCCGTGCTGCCGCCGCATCTGCTGGAC
DENND1A.V3         CTGACTGCCTGCATCCACGGGTCTGCGGCGATGCTCTACCCCATGTACTGGCAGCACGTGTACATCCCCGTGCTGCCGCCGCATCTGCTGGAC
DENND1A.V4         CTGACTGCCTGCATCCACGGGTCTGCGGCGATGCTCTACCCCATGTACTGGCAGCACGTGTACATCCCCGTGCTGCCGCCGCATCTGCTGGAC
DENND1A.V1,3,4_F   ---------------------------------------------------------------------------------------------
DENND1A.V1,3,4_R   ---------------------------------------------------------------------------------------------

DENND1A.V1         TACTGCTGgtaaggtggctggccctgtcctctgcttctcctttgccctg..........aaagcagctgtgtgtctctgtttctcttcacag
DENND1A.V3         TACTGCTG-------------------------------------------------------------------------------------
DENND1A.V4         TACTGCTG-------------------------------------------------------------------------------------
DENND1A.V1,3,4_F   ---------------------------------------------------------------------------------------------
DENND1A.V1,3,4_R   ---------------------------------------------------------------------------------------------   Exon 10

DENND1A.V1         TGCTCCCATGCCCTACCTCATAGGAATCCATTTAAGTTTAATGGAGgtaagttggcttctttcct.........gtaagttggcttctttcct
DENND1A.V3         TGCTCCCATGCCCTACCTCATAGGAATCCATTTAAGTTTAATGGAG-----------------------------------------------
DENND1A.V4         TGCTCCCATGCCCTACCTCATAGGAATCCATTTAAGTTTAATGGAG-----------------------------------------------
DENND1A.V1,3,4_F   ---------------------------------------------------------------------------------------------
DENND1A.V1,3,4_R   ---------------------------------------------------------------------------------------------   Exon 11

DENND1A.V1         AAAGTCAGAAACATGGCCCTGGATGATGTCGTGATCCTGAATGTGGACACCAACACCCTGGAAACCCCCTTCGATGACCTCCAGAGCCTCCCA
DENND1A.V3         AAAGTCAGAAACATGGCCCTGGATGATGTCGTGATCCTGAATGTGGACACCAACACCCTGGAAACCCCCTTCGATGACCTCCAGAGCCTCCCA
DENND1A.V4         AAAGTCAGAAACATGGCCCTGGATGATGTCGTGATCCTGAATGTGGACACCAACACCCTGGAAACCCCCTTCGATGACCTCCAGAGCCTCCCA
DENND1A.V1,3,4_F   ---------------------------------------------------------------------------------------------
DENND1A.V1,3,4_R   ---------------------------------------------------------------------------------------------

DENND1A.V1         AACGACGTGgtaggtaatgagcttgcgaggatctcacttctg.....................tgtgttttccttctctccatcaccag
DENND1A.V3         AACGACGTG------------------------------------------------------------------------------------
DENND1A.V4         AACGACGTG------------------------------------------------------------------------------------
DENND1A.V1,3,4_F   ---------------------------------------------------------------------------------------------
DENND1A.V1,3,4_R   ---------------------------------------------------------------------------------------------   Exon 12

DENND1A.V1         ATCTCTTCCCTGAAGAACAGGCTGAAAAAGGTCTCCACAACCACTGGGGATGGTGTGGCCAGAGCGTTCCTCAAGGCCCAGGCTGCTTTCTTC
DENND1A.V3         ATCTCTTCCCTGAAGAACAGGCTGAAAAAGGTCTCCACAACCACTGGGGATGGTGTGGCCAGAGCGTTCCTCAAGGCCCAGGCTGCTTTCTTC
DENND1A.V4         ATCTCTTCCCTGAAGAACAGGCTGAAAAAGGTCTCCACAACCACTGGGGATGGTGTGGCCAGAGCGTTCCTCAAGGCCCAGGCTGCTTTCTTC
DENND1A.V1,3,4_F   ---------------------------------------------------------------------------------------------
DENND1A.V1,3,4_R   ---------------------------------------------------------------------------------------------

DENND1A.V1         GGTAGCTACCGAAACGCTCTGAAAATCGAGCCGgtgagtagccttggcatgtca..........cccacctctcccctctgcacttcccag
DENND1A.V3         GGTAGCTACCGAAACGCTCTGAAAATCGAGCCG------------------------------------------------------------
DENND1A.V4         GGTAGCTACCGAAACGCTCTGAAAATCGAGCCG------------------------------------------------------------
DENND1A.V1,3,4_F   ---------------------------------------------------------------------------------------------
DENND1A.V1,3,4_R   ---------------------------------------------------------------------------------------------   Exon 13

DENND1A.V1         GAGGAGCCGATCACTTTCTGTGAGGAAGCCTTCGTGTCCCACTACCGCTCCGGAGCCATGAGGCAGTTCCTGCAGAACGCCACACAGCTGCAG
DENND1A.V3         GAGGAGCCGATCACTTTCTGTGAGGAAGCCTTCGTGTCCCACTACCGCTCCGGAGCCATGAGGCAGTTCCTGCAGAACGCCACACAGCTGCAG
DENND1A.V4         GAGGAGCCGATCACTTTCTGTGAGGAAGCCTTCGTGTCCCACTACCGCTCCGGAGCCATGAGGCAGTTCCTGCAGAACGCCACACAGCTGCAG
DENND1A.V1,3,4_F   ---------------------------------------------------------------------------------------------
DENND1A.V1,3,4_R   ---------------------------------------------------------------------------------------------   Exon 14

DENND1A.V1         CTCTTCAAGCAGgtgcctccctccctggtctggcctgggtctgg........ttgtacgagctgttgtgcttttgttccttctttctatag
DENND1A.V3         CTCTTCAAGCAG---------------------------------------------------------------------------------
DENND1A.V4         CTCTTCAAGCAG---------------------------------------------------------------------------------   Exon 14
```

```
DENND1A.V1,3,4_F   ---------------------------------------------------------------------------------------------
DENND1A.V1,3,4_R   ---------------------------------------------------------------------------------------------

DENND1A.V1         TTTATTGATGGTCGATTAGATCTTCTCAATTCCGGCGAAGGTTTCAGTGATGTTTTTGAAGAGGAAATCAACATGGGCGAGTACGCTGgt…ag
DENND1A.V3         TTTATTGATGGTCGATTAGATCTTCTCAATTCCGGCGAAGGTTTCAGTGATGTTTTTGAAGAGGAAATCAACATGGGCGAGTACGCTG-----   Exon 15
DENND1A.V4         TTTATTGATGGTCGATTAGATCTTCTCAATTCCGGCGAAGGTTTCAGTGATGTTTTTGAAGAGGAAATCAACATGGGCGAGTACGCTG-----
DENND1A.V1,3,4_F   ---------------------------------------------------------------------------------------------
DENND1A.V1,3,4_R   ---------------------------------------------------------------------------------------------

DENND1A.V1         GCAGTGACAAACTGTACCATCAGTGGCTCTCCACTGTCCGGgtaagcatgcacccaattcaga………aattgctaattgacatttttattatag
DENND1A.V3         GCAGTGACAAACTGTACCATCAGTGGCTCTCCACTGTCCGG----------------------------------------------------   Exon 16
DENND1A.V4         GCAGTGACAAACTGTACCATCAGTGGCTCTCCACTGTCCGG----------------------------------------------------
DENND1A.V1,3,4_F   ---------------------------------------------------------------------------------------------
DENND1A.V1,3,4_R   ---------------------------------------------------------------------------------------------

DENND1A.V1         AAAGGAAGTGGAGCAATTCTGAATACTGTAAAGACCAAAGCAAATCCGGCCATGAAGACTGTCTACAAGTTCgtaagtact……tttcatgcag
DENND1A.V3         AAAGGAAGTGGAGCAATTCTGAATACTGTAAAGACCAAAGCAAATCCGGCCATGAAGACTGTCTACAAGTTC---------------------   Exon 17
DENND1A.V4         AAAGGAAGTGGAGCAATTCTGAATACTGTAAAGACCAAAGCAAATCCGGCCATGAAGACTGTCTACAAGTTC---------------------
DENND1A.V1,3,4_F   ---------------------------------------------------------------------------------------------
DENND1A.V1,3,4_R   ---------------------------------------------------------------------------------------------

DENND1A.V1         GCAAAAGATCATGCAAAAATGGGAATAAAAGAGGTGAAAAACCGCTTGAAGCAAAAGgtacttgaagttcttatt…………ccttctccctacag
DENND1A.V3         GCAAAAGATCATGCAAAAATGGGAATAAAAGAGGTGAAAAACCGCTTGAAGCAAAAG------------------------------------   Exon 18
DENND1A.V4         GCAAAAGATCATGCAAAAATGGGAATAAAAGAGGTGAAAAACCGCTTGAAGCAAAAG------------------------------------
DENND1A.V1,3,4_F   ---------------------------------------------------------------------------------------------
DENND1A.V1,3,4_R   ---------------------------------------------------------------------------------------------

DENND1A.V1         GACATTGCCGAGAATGGCTGCGCCCCCACCCCAGAAGAGCAGCTGCCAAAGACTGCACCGTCCCCACTGGTGGAGGCCAAGGACCCCAAGCTC
DENND1A.V3         GACATTGCCGAGAATGGCTGCGCCCCCACCCCAGAAGAGCAGCTGCCAAAGACTGCACCGTCCCCACTGGTGGAGGCCAAGGACCCCAAGCTC
DENND1A.V4         GACATTGCCGAGAATGGCTGCGCCCCCACCCCAGAAGAGCAGCTGCCAAAGACTGCACCGTCCCCACTGGTGGAGGCCAAGGACCCCAAGCTC
DENND1A.V1,3,4_F   ---------------------------------------------------------------------------------------------
DENND1A.V1,3,4_R   ---------------------------------------------------------------------------------------------

DENND1A.V1         CGAGAAGACCGGCGGCCAATCACAGTCCACTTTGGACAGgtgtgtaccctggccctcct………ttccactttaatgcagctgcagagactgcgt
DENND1A.V3         CGAGAAGACCGGCGGCCAATCACAGTCCACTTTGGACAG---------------------------------------CTGCAGAGACTGCGT   Exon 19
DENND1A.V4         CGAGAAGACCGGCGGCCAATCACAGTCCACTTTGGACAG------------------------------------------------------
DENND1A.V1,3,4_F   ---------------------------------------------------------------------------------------------
DENND1A.V1,3,4_R   ---------------------------------------------------------------------------------------------

DENND1A.V1         cccacccgaccgcctcccaagatacagcgctcgaggcccgtgagtagctgg…………………………………………………………………ccccctgtacctcctctag
DENND1A.V3         CCCACCCGACCGCCTCCCAAGATACAGCGCTCGAGGCCCG-----------------------------------------------------
DENND1A.V4         ---------------------------------------------------------------------------------------------
DENND1A.V1,3,4_F   ---------------------------------------------------------------------------------------------
DENND1A.V1,3,4_R   ---------------------------------------------------------------------------------------------

DENND1A.V1         GTGCGCCCACCTCGTCCACATGTTGTTAAGAGACCAAAGAGCAACATCGCAGTGGAAGGCCGGAGGACGTCTGTGCCGAGCCCTGAGCAgtg…
DENND1A.V3         GTGCGCCCACCTCGTCCACATGTTGTTAAGAGACCAAAGAGCAACATCGCAGTGGAAGGCCGGAGGACGTCTGTGCCGAGCCCTGAGCA----
DENND1A.V4         GTGCGCCCACCTCGTCCACATGTTGTTAAGAGACCAAAGAGCAACATCGCAGTGGAAGGCCGGAGGACGTCTGTGCCGAGCCCTGAGCA----
DENND1A.V1,3,4_F   ---------------------------------------------------------------------------------------------
DENND1A.V1,3,4_R   ---------------------------------------------------------------------------------------------

DENND1A.V1         ccccagcctggtaaagcccttgcgacactatgcggtcttcctctccgaagactcctctgatgatgaatgccagcgggaagagggcccgagctc
DENND1A.V3         ------CCTGGTAAAGCCCTTGCGACACTATGCGGTCTTCCTCTCCGAAGACTCCTCTGATGATGAATGCCAGCGGGAAGAGGGCCCGAGCTC   Exon 20
DENND1A.V4         ------CCTGGTAAAGCCCTTGCGACACTATGCGGTCTTCCTCTCCGAAGACTCCTCTGATGATGAATGCCAGCGGGAAGAGGGCCCGAGCTC
DENND1A.V1,3,4_F   ---------------------------------------------------------------------------------------------
DENND1A.V1,3,4_R   ---------------------------------------------------------------------------------------------

DENND1A.V1         tggcttcaccgagagctttttcttctccgctccctttgaatggtctctccttcc……………………………………………………………cctgctctgtccccgacag
DENND1A.V3         TGGCTTCACCGAGAGCTTTTTCTTCTCCGCTCCCTTTGAATGG--------------------------------------------------
DENND1A.V4         TGGCTTCACCGAGAGCTTTTTCTTCTCCGCTCCCTTTGAATGG--------------------------------------------------
DENND1A.V1,3,4_F   ---------------------------------------------------------------------------------------------
DENND1A.V1,3,4_R   ---------------------------------------------------------------------------------------------

DENND1A.V1         GCCGCAGCCGTATCGGACACTCAGGGAGTCAGACAGCGCGGAAGGCGACGAGGCAGAGAGTCCAGAGCAGCAAGTGCGGAAGTCCACAGGCCC
DENND1A.V3         GCCGCAGCCGTATCGGACACTCAGGGAGTCAGACAGCGCGGAAGGCGACGAGGCAGAGAGTCCAGAGCAGCAAGTGCGGAAGTCCACAGGCCC   Exon 21
DENND1A.V4         GCCGCAGCCGTATCGGACACTCAGGGAGTCAGACAGCGCGGAAGGCGACGAGGCAGAGAGTCCAGAGCAGCAAGTGCGGAAGTCCACAGGCCC
DENND1A.V1,3,4_F   ---------------------------------------------------------------------------------------------
DENND1A.V1,3,4_R   ---------------------------------------------------------------------------------------------

DENND1A.V1         TGTCCCAGCTCCCCCTGACCGGGCTGCCAGCATCGACCTTCTGGAAGACGTCTTCAGCAACCTGGACATGGAGGCCGCACTGCAGCCACTGGG
DENND1A.V3         TGTCCCAGCTCCCCCTGACCGGGCTGCCAGCATCGACCTTCTGGAAGACGTCTTCAGCAACCTGGACATGGAGGCCGCACTGCAGCCACTGGG
DENND1A.V4         TGTCCCAGCTCCCCCTGACCGGGCTGCCAGCATCGACCTTCTGGAAGACGTCTTCAGCAACCTGGACATGGAGGCCGCACTGCAGCCACTGGG
```

```
DENND1A.V1,3,4_F    ------------------------------------------------------------------------------------------
DENND1A.V1,3,4_R    ------------------------------------------------------------------------------------------

DENND1A.V1          CCAGGCCAAGAGCTTAGAGGACCTTCGTGCCCCCAAAGACCTGAGGGAGCAGCCAGGGACCTTTGACTATCAGgtatggc……………ccaacag
DENND1A.V3          CCAGGCCAAGAGCTTAGAGGACCTTCGTGCCCCCAAAGACCTGAGGGAGCAGCCAGGGACCTTTGACTATCAG-----------------
DENND1A.V4          CCAGGCCAAGAGCTTAGAGGACCTTCGTGCCCCCAAAGACCTGAGGGAGCAGCCAGGGACCTTTGACTATCAG-----------------
DENND1A.V1,3,4_F    -------------------------------------------------CAGCCAGGGACCTTTGACTA---------------------
DENND1A.V1,3,4_R    ------------------------------------------------------------------------------------------

DENND1A.V1          AGGCTGGATCTGGGCGGGAGTGAGGAGCCGCGGGGTGACAGTGGCCTTGAAGCTTACCCACCCGTACAACAAGCTCTGGAGCCTGGGCAG
DENND1A.V3          AGGCTGGATCTGGGCGGGAGTGAGGAGCCGCGGGGTGACAGTGGCCTTGAAGCTTACCCACCCGTACAACAAGCTCTGGAGCCTGGGCAG
DENND1A.V4          AGGCTGGATCTGGGCGGGAGTGAGGAGCCGCGGGGTGACAGTGGCCTTGAAGCTTACCCACCCGTACAACAAGCTCTGGAGCCTGGGCAG
DENND1A.V1,3,4_F    ------------------------------------------------------------------------------------------
DENND1A.V1,3,4_R    ----------------------------------------------------------CACCCGTACAACAAGCTCTG------------

DENND1A.V1          GACGACATGGCCATCCCCAGCAAGCCCCCAGCTGCCTCCCCTGAGAAGCCCTCGGCCCTGCTCGGGAACTCCCTGGCCCTGCCTCGAAGGCCC
DENND1A.V3          GACGACATGGCCATCCCCAGCAAGCCCCCAGCTGCCTCCCCTGAGAAGCCCTCGGCCCTGCTCGGGAACTCCCTGGCCCTGCCTCGAAGGCCC
DENND1A.V4          GACGACATGGCCATCCCCAGCAAGCCCCCAGCTGCCTCCCCTGAGAAGCCCTCGGCCCTGCTCGGGAACTCCCTGGCCCTGCCTCGAAGGCCC
DENND1A.V1,3,4_F    ------------------------------------------------------------------------------------------
DENND1A.V1,3,4_R    ------------------------------------------------------------------------------------------

DENND1A.V1          CAGAACCGGGACAGCATCCTGAACCCCAGTGACAAGGAGGAGGTGCCCACCCCTACTCTGGGCAGCATCACCATCCCCCGGCCCCAAGGCAG
DENND1A.V3          CAGAACCGGGACAGCATCCTGAACCCCAGTGACAAGGAGGAGGTGCCCACCCCTACTCTGGGCAGCATCACCATCCCCCGGCCCCAAGGCAG
DENND1A.V4          CAGAACCGGGACAGCATCCTGAACCCCAGTGACAAGGAGGAGGTGCCCACCCCTACTCTGGGCAGCATCACCATCCCCCGGCCCCAAGGCAG
DENND1A.V1,3,4_F    ------------------------------------------------------------------------------------------
DENND1A.V1,3,4_R    ------------------------------------------------------------------------------------------

DENND1A.V1          GAAGACCCCAGAGCTGGGCATCGTGCCTCCACCGCCCATTCCCCGCCCGGCCAAGCTCCAGGCTGCCGGCGCCGCACTTGGTGACGTCTCAGA
DENND1A.V3          GAAGACCCCAGAGCTGGGCATCGTGCCTCCACCGCCCATTCCCCGCCCGGCCAAGCTCCAGGCTGCCGGCGCCGCACTTGGTGACGTCTCAGA
DENND1A.V4          GAAGACCCCAGAGCTGGGCATCGTGCCTCCACCGCCCATTCCCCGCCCGGCCAAGCTCCAGGCTGCCGGCGCCGCACTTGGTGACGTCTCAGA
DENND1A.V1,3,4_F    ------------------------------------------------------------------------------------------
DENND1A.V1,3,4_R    ------------------------------------------------------------------------------------------

DENND1A.V1          GCGGCTGCAGACGGATCGGGACAGGCGAGCTGCCCTGAGTCCAGGGCTCCTGCCTGGTGTTGTCCCCCAAGGCCCCACTGAACTGCTCCAGCC
DENND1A.V3          GCGGCTGCAGACGGATCGGGACAGGCGAGCTGCCCTGAGTCCAGGGCTCCTGCCTGGTGTTGTCCCCCAAGGCCCCACTGAACTGCTCCAGCC
DENND1A.V4          GCGGCTGCAGACGGATCGGGACAGGCGAGCTGCCCTGAGTCCAGGGCTCCTGCCTGGTGTTGTCCCCCAAGGCCCCACTGAACTGCTCCAGCC
DENND1A.V1,3,4_F    ------------------------------------------------------------------------------------------
DENND1A.V1,3,4_R    ------------------------------------------------------------------------------------------

DENND1A.V1          GCTCAGCCCTGGCCCCGGGGCTGCAGGCACGAGCAGTGACGCCCTGCTCGCCCTCCTGGACCCGCTCAGCACAGCCTGGTCAGGCAGCACCCT
DENND1A.V3          GCTCAGCCCTGGCCCCGGGGCTGCAGGCACGAGCAGTGACGCCCTGCTCGCCCTCCTGGACCCGCTCAGCACAGCCTGGTCAGGCAGCACCCT
DENND1A.V4          GCTCAGCCCTGGCCCCGGGGCTGCAGGCACGAGCAGTGACGCCCTGCTCGCCCTCCTGGACCCGCTCAGCACAGCCTGGTCAGGCAGCACCC
DENND1A.V1,3,4_F    ------------------------------------------------------------------------------------------
DENND1A.V1,3,4_R    ------------------------------------------------------------------------------------------

DENND1A.V1          CCCGTCACGCCCCGCCACCCCGAATGTAGCCACCCCATTCACCCCCCAATTCAGCTTCCCCCCTGCAGGGACACCCACCCCATTCCCACAGCC
DENND1A.V3          CCCGTCACGCCCCGCCACCCCGAATGTAGCCACCCCATTCACCCCCCAATTCAGCTTCCCCCCTGCAGGGACACCCACCCCATTCCCACAGCC
DENND1A.V4          CCCGTCACGCCCCGCCACCCCGAATGTAGCCACCCCATTCACCCCCCAATTCAGCTTCCCCCCTGCAGGGACACCCACCCCATTCCCACAGCC
DENND1A.V1,3,4_F    ------------------------------------------------------------------------------------------
DENND1A.V1,3,4_R    ------------------------------------------------------------------------------------------

DENND1A.V1          ACCACTCAACCCCTTTGTCCCATCCATGCCAGCAGCCCCACCCACCCTGCCCCTGGTCTCCACACCAGCCGGGCCTTTCGGGGCCCCTCCAGC
DENND1A.V3          ACCACTCAACCCCTTTGTCCCATCCATGCCAGCAGCCCCACCCACCCTGCCCCTGGTCTCCACACCAGCCGGGCCTTTCGGGGCCCCTCCAGC
DENND1A.V4          ACCACTCAACCCCTTTGTCCCATCCATGCCAGCAGCCCCACCCACCCTGCCCCTGGTCTCCACACCAGCCGGGCCTTTCGGGGCCCCTCCAGC
DENND1A.V1,3,4_F    ------------------------------------------------------------------------------------------
DENND1A.V1,3,4_R    ------------------------------------------------------------------------------------------

DENND1A.V1          TTCCCTGGGGCCGGCTTTTGCGTCCGGCCTCCTGCTGTCCAGTGCTGGCTTCTGTGCCCCTCACAGGTCTCAGCCCAACCTCTCCGCCCTCTC
DENND1A.V3          TTCCCTGGGGCCGGCTTTTGCGTCCGGCCTCCTGCTGTCCAGTGCTGGCTTCTGTGCCCCTCACAGGTCTCAGCCCAACCTCTCCGCCCTCTC
DENND1A.V4          TTCCCTGGGGCCGGCTTTTGCGTCCGGCCTCCTGCTGTCCAGTGCTGGCTTCTGTGCCCCTCACAGGTCTCAGCCCAACCTCTCCGCCCTCTC
DENND1A.V1,3,4_F    ------------------------------------------------------------------------------------------
DENND1A.V1,3,4_R    ------------------------------------------------------------------------------------------

DENND1A.V1          CATGCCCAACCTCTTTGGCCAGATGCCCATGGGCACCCACACGAGCCCCCTACAGCCGCTGGGTCCCCCAGCAGTTGCCCCGTCGAGGATCCG
DENND1A.V3          CATGCCCAACCTCTTTGGCCAGATGCCCATGGGCACCCACACGAGCCCCCTACAGCCGCTGGGTCCCCCAGCAGTTGCCCCGTCGAGGATCCG
DENND1A.V4          CATGCCCAACCTCTTTGGCCAGATGCCCATGGGCACCCACACGAGCCCCCTACAGCCGCTGGGTCCCCCAGCAGTTGCCCCGTCGAGGATCCG
DENND1A.V1,3,4_F    ------------------------------------------------------------------------------------------
DENND1A.V1,3,4_R    ------------------------------------------------------------------------------------------

DENND1A.V1          AACGTTGCCCCTGGCCCGCTCAAGTGCCAGGGCTGCTGAGACCAAGCAGGGGCTGGCCCTGAGGCCTGGAGACCCCCCGCTTCTGCCTCCCAG
DENND1A.V3          AACGTTGCCCCTGGCCCGCTCAAGTGCCAGGGCTGCTGAGACCAAGCAGGGGCTGGCCCTGAGGCCTGGAGACCCCCCGCTTCTGCCTCCCAG
DENND1A.V4          AACGTTGCCCCTGGCCCGCTCAAGTGCCAGGGCTGCTGAGACCAAGCAGGGGCTGGCCCTGAGGCCTGGAGACCCCCCGCTTCTGCCTCCCAG
```

Exon 21

Exon 22

```
DENND1A.V1,3,4_F    ----------------------------------------------------------------------------------------------------
DENND1A.V1,3,4_R    ----------------------------------------------------------------------------------------------------

DENND1A.V1          GCCCCCTCAAGGCCTGGAGCCAACACTGCAGCCCTCTGCTCCTCAACAGGCCAGAGACCCCTTTGAGGATTTGTTACAGAAAACCAAGCAAGA
DENND1A.V3          GCCCCCTCAAGGCCTGGAGCCAACACTGCAGCCCTCTGCTCCTCAACAGGCCAGAGACCCCTTTGAGGATTTGTTACAGAAAACCAAGCAAGA
DENND1A.V4          GCCCCCTCAAGGCCTGGAGCCAACACTGCAGCCCTCTGCTCCTCAACAGGCCAGAGACCCCTTTGAGGATTTGTTACAGAAAACCAAGCAAGA
DENND1A.V1,3,4_F    ----------------------------------------------------------------------------------------------------
DENND1A.V1,3,4_R    ----------------------------------------------------------------------------------------------------

DENND1A.V1          CGTGAGCCCGAGTCCGGCCCTGGCCCCGGCCCCAGACTCGGTGGAGCAGCTCAGGAAGCAGTGGGAGACCTTCGAGTGAGCCGGGCCCTGAGG
DENND1A.V3          CGTGAGCCCGAGTCCGGCCCTGGCCCCGGCCCCAGACTCGGTGGAGCAGCTCAGGAAGCAGTGGGAGACCTTCGAGTGAGCCGGGCCCTGAGG
DENND1A.V4          CGTGAGCCCGAGTCCGGCCCTGGCCCCGGCCCCAGACTCGGTGGAGCAGCTCAGGAAGCAGTGGGAGACCTTCGAGTGAGCCGGGCCCTGAGG
DENND1A.V1,3,4_F    ----------------------------------------------------------------------------------------------------
DENND1A.V1,3,4_R    ----------------------------------------------------------------------------------------------------

DENND1A.V1          GTGGGGGATGCACCGAGGCCCGAGGGTCCGTCCACTGCTGCGGTTCCGAGGCTCCCCCGCCACTCTCTCTCTGCCCAGGTTCTGCTGGTGGGA
DENND1A.V3          GTGGGGGATGCACCGAGGCCCGAGGGTCCGTCCACTGCTGCGGTTCCGAGGCTCCCCCGCCACTCTCTCTCTGCCCAGGTTCTGCTGGTGGGA
DENND1A.V4          GTGGGGGATGCACCGAGGCCCGAGGGTCCGTCCACTGCTGCGGTTCCGAGGCTCCCCCGCCACTCTCTCTCTGCCCAGGTTCTGCTGGTGGGA
DENND1A.V1,3,4_F    ----------------------------------------------------------------------------------------------------
DENND1A.V1,3,4_R    ----------------------------------------------------------------------------------------------------

DENND1A.V1          AGGGATGGGACCCCTCTCTGCTGCCCCCTCCTCCCCTCCACACTGCCCATCTCTGATGTCTGGCCCTGGGGAATGGCACCAGTTCCAGCCTGG
DENND1A.V3          AGGGATGGGACCCCTCTCTGCTGCCCCCTCCTCCCCTCCACACTGCCCATCTCTGATGTCTGGCCCTGGGGAATGGCACCAGTTCCAGCCTGG
DENND1A.V4          AGGGATGGGACCCCTCTCTGCTGCCCCCTCCTCCCCTCCACACTGCCCATCTCTGATGTCTGGCCCTGGGGAATGGCACCAGTTCCAGCCTGG
DENND1A.V1,3,4_F    ----------------------------------------------------------------------------------------------------
DENND1A.V1,3,4_R    ----------------------------------------------------------------------------------------------------

DENND1A.V1          GAATCAACCCAGTTCCTGAGTGCCCATCCCACCCCGCGGTTGCCTCTCCTCGGCACCCTTGATTGGGTTTTGCACTAAAGAGGTCAGCTGGGC
DENND1A.V3          GAATCAACCCAGTTCCTGAGTGCCCATCCCACCCCGCGGTTGCCTCTCCTCGGCACCCTTGATTGGGTTTTGCACTAAAGAGGTCAGCTGGGC
DENND1A.V4          GAATCAACCCAGTTCCTGAGTGCCCATCCCACCCCGCGGTTGCCTCTCCTCGGCACCCTTGATTGGGTTTTGCACTAAAGAGGTCAGCTGGGC
DENND1A.V1,3,4_F    ----------------------------------------------------------------------------------------------------
DENND1A.V1,3,4_R    ----------------------------------------------------------------------------------------------------

DENND1A.V1          CAATGATATTGCTCCAGACCGAGTCCTACCCACCTTCCCCCGGAAGTGTCCCAAGAGGCTCCGAAGGCCTCCCCTCCGAGCCCAGCTCTCCTG
DENND1A.V3          CAATGATATTGCTCCAGACCGAGTCCTACCCACCTTCCCCCGGAAGTGTCCCAAGAGGCTCCGAAGGCCTCCCCTCCGAGCCCAGCTCTCCTG
DENND1A.V4          CAATGATATTGCTCCAGACCGAGTCCTACCCACCTTCCCCCGGAAGTGTCCCAAGAGGCTCCGAAGGCCTCCCCTCCGAGCCCAGCTCTCCTG
DENND1A.V1,3,4_F    ----------------------------------------------------------------------------------------------------
DENND1A.V1,3,4_R    ----------------------------------------------------------------------------------------------------

DENND1A.V1          TCTCCTCCACAGCCAGGCCCTGCACGCCCACCTCCTCGGACACAGGTGACAGGGTTACCCTCCAGTTTGAGCTCATCTGCACGAGACACAGGT
DENND1A.V3          TCTCCTCCACAGCCAGGCCCTGCACGCCCACCTCCTCGGACACAGGTGACAGGGTTACCCTCCAGTTTGAGCTCATCTGCACGAGACACAGGT
DENND1A.V4          TCTCCTCCACAGCCAGGCCCTGCACGCCCACCTCCTCGGACACAGGTGACAGGGTTACCCTCCAGTTTGAGCTCATCTGCACGAGACACAGGT
DENND1A.V1,3,4_F    ----------------------------------------------------------------------------------------------------
DENND1A.V1,3,4_R    ----------------------------------------------------------------------------------------------------

DENND1A.V1          AGCTTGGGGTTGAAGTTAGGACTCCTCCTGGGCTGGAGGATTTACCTGGTGGGGCACTTCCAGACTGTTTCTAGCAATATACACACACGTTCT
DENND1A.V3          AGCTTGGGGTTGAAGTTAGGACTCCTCCTGGGCTGGAGGATTTACCTGGTGGGGCACTTCCAGACTGTTTCTAGCAATATACACACACGTTCT
DENND1A.V4          AGCTTGGGGTTGAAGTTAGGACTCCTCCTGGGCTGGAGGATTTACCTGGTGGGGCACTTCCAGACTGTTTCTAGCAATATACACACACGTTCT
DENND1A.V1,3,4_F    ----------------------------------------------------------------------------------------------------
DENND1A.V1,3,4_R    ----------------------------------------------------------------------------------------------------

DENND1A.V1          TTCCTGTGTCTTCACCCCAAAACTTCAGTTGATTCTGACCTGGGAGGATCTGGGGACCAGGGGGTCTTGGGCTGCCTTGTGATACACAGCCCC
DENND1A.V3          TTCCTGTGTCTTCACCCCAAAACTTCAGTTGATTCTGACCTGGGAGGATCTGGGGACCAGGGGGTCTTGGGCTGCCTTGTGATACACAGCCCC
DENND1A.V4          TTCCTGTGTCTTCACCCCAAAACTTCAGTTGATTCTGACCTGGGAGGATCTGGGGACCAGGGGGTCTTGGGCTGCCTTGTGATACACAGCCCC
DENND1A.V1,3,4_F    ----------------------------------------------------------------------------------------------------
DENND1A.V1,3,4_R    ----------------------------------------------------------------------------------------------------

DENND1A.V1          AGCCACCCTGCACGGGGGCTGCGAGCACCAGCAACTTTGATTTATAGAAGGAAAATGGAAACCCCCATCTGAGTATTTTGGGAGGAGCCCCCA
DENND1A.V3          AGCCACCCTGCACGGGGGCTGCGAGCACCAGCAACTTTGATTTATAGAAGGAAAATGGAAACCCCCATCTGAGTATTTTGGGAGGAGCCCCCA
DENND1A.V4          AGCCACCCTGCACGGGGGCTGCGAGCACCAGCAACTTTGATTTATAGAAGGAAAATGGAAACCCCCATCTGAGTATTTTGGGAGGAGCCCCCA
DENND1A.V1,3,4_F    ----------------------------------------------------------------------------------------------------
DENND1A.V1,3,4_R    ----------------------------------------------------------------------------------------------------

DENND1A.V1          GCCCTCATCCAGCTCTGGCACGCTGATACCTCCAGGTACTCCCCTCACTGTCAAAGCTGGGGCTCAGCCTCTTGTCATCTGGAGCTTTGTGGG
DENND1A.V3          GCCCTCATCCAGCTCTGGCACGCTGATACCTCCAGGTACTCCCCTCACTGTCAAAGCTGGGGCTCAGCCTCTTGTCATCTGGAGCTTTGTGGG
DENND1A.V4          GCCCTCATCCAGCTCTGGCACGCTGATACCTCCAGGTACTCCCCTCACTGTCAAAGCTGGGGCTCAGCCTCTTGTCATCTGGAGCTTTGTGGG
DENND1A.V1,3,4_F    ----------------------------------------------------------------------------------------------------
DENND1A.V1,3,4_R    ----------------------------------------------------------------------------------------------------

DENND1A.V1          CAAAGCTGAGAAGCTGCAACCCAGATTTCAACCCAAAAAGGTCAAGCTGAATGCCTCAGACTGATGTGGAAGGCAGCTGGCCTTCCTGGGTTG
DENND1A.V3          CAAAGCTGAGAAGCTGCAACCCAGATTTCAACCCAAAAAGGTCAAGCTGAATGCCTCAGACTGATGTGGAAGGCAGCTGGCCTTCCTGGGTTG
DENND1A.V4          CAAAGCTGAGAAGCTGCAACCCAGATTTCAACCCAAAAAGGTCAAGCTGAATGCCTCAGACTGATGTGGAAGGCAGCTGGCCTTCCTGGGTTG
```

Exon 22

```
DENND1A.V1,3,4_F   ---------------------------------------------------------------------------------------------
DENND1A.V1,3,4_R   ---------------------------------------------------------------------------------------------

DENND1A.V1         GAACGAGGCAGTGGCCCTGAGCCCCTTCTCCAGGGCCAGGTAGAAAGGACAAACTTGGTCTCTGCCTCGGGGAAGCAGGAGGAGGGCTAGAAG
DENND1A.V3         GAACGAGGCAGTGGCCCTGAGCCCCTTCTCCAGGGCCAGGTAGAAAGGACAAACTTGGTCTCTGCCTCGGGGAAGCAGGAGGAGGGCTAGAAG
DENND1A.V4         GAACGAGGCAGTGGCCCTGAGCCCCTTCTCCAGGGCCAGGTAGAAAGGACAAACTTGGTCTCTGCCTCGGGGAAGCAGGAGGAGGGCTAGAAG
DENND1A.V1,3,4_F   ---------------------------------------------------------------------------------------------
DENND1A.V1,3,4_R   ---------------------------------------------------------------------------------------------

DENND1A.V1         CCAGTCCCTCCCCACCTGCCCAGAGCTCCAGGCCAGCACAGAAATTCCTGAGGCCAACGTCACCAAAGTTAGATTGAATGTTTATTATCTTTC
DENND1A.V3         CCAGTCCCTCCCCACCTGCCCAGAGCTCCAGGCCAGCACAGAAATTCCTGAGGCCAACGTCACCAAAGTTAGATTGAATGTTTATTATCTTTC
DENND1A.V4         CCAGTCCCTCCCCACCTGCCCAGAGCTCCAGGCCAGCACAGAAATTCCTGAGGCCAACGTCACCAAAGTTAGATTGAATGTTTATTATCTTTC
DENND1A.V1,3,4_F   ---------------------------------------------------------------------------------------------
DENND1A.V1,3,4_R   ---------------------------------------------------------------------------------------------

DENND1A.V1         TTTTTCCTTTTTACCTTATTGATTTGATGAATCTTGAAATGGATTCATTTCCATAAACCAAGTTAAAGTATGGCCCGACCATTTAAGAAAACA
DENND1A.V3         TTTTTCCTTTTTACCTTATTGATTTGATGAATCTTGAAATGGATTCATTTCCATAAACCAAGTTAAAGTATGGCCCGACCATTTAAGAAAACA
DENND1A.V4         TTTTTCCTTTTTACCTTATTGATTTGATGAATCTTGAAATGGATTCATTTCCATAAACCAAGTTAAAGTATGGCCCGACCATTTAAGAAAACA
DENND1A.V1,3,4_F   ---------------------------------------------------------------------------------------------
DENND1A.V1,3,4_R   ---------------------------------------------------------------------------------------------

DENND1A.V1         ACCATCTGAGACACGCAGGAAATTGTGAGCATTTCGACCCGAGCTCTCATTTCCTATTTGTGAAGGGTCAGACACAGTCTACCCAGGGGTGTC
DENND1A.V3         ACCATCTGAGACACGCAGGAAATTGTGAGCATTTCGACCCGAGCTCTCATTTCCTATTTGTGAAGGGTCAGACACAGTCTACCCAGGGGTGTC
DENND1A.V4         ACCATCTGAGACACGCAGGAAATTGTGAGCATTTCGACCCGAGCTCTCATTTCCTATTTGTGAAGGGTCAGACACAGTCTACCCAGGGGTGTC
DENND1A.V1,3,4_F   ---------------------------------------------------------------------------------------------
DENND1A.V1,3,4_R   ---------------------------------------------------------------------------------------------

DENND1A.V1         TGGGGGACAAGGGGGTCTCTGGAGATGTCACCCAGGGAGCCCCCTCTATGTCTGAGAGGCTGCCACTGCTGCACATGCTCAGTGAGGCTTGGC
DENND1A.V3         TGGGGGACAAGGGGGTCTCTGGAGATGTCACCCAGGGAGCCCCCTCTATGTCTGAGAGGCTGCCACTGCTGCACATGCTCAGTGAGGCTTGGC
DENND1A.V4         TGGGGGACAAGGGGGTCTCTGGAGATGTCACCCAGGGAGCCCCCTCTATGTCTGAGAGGCTGCCACTGCTGCACATGCTCAGTGAGGCTTGGC
DENND1A.V1,3,4_F   ---------------------------------------------------------------------------------------------
DENND1A.V1,3,4_R   ---------------------------------------------------------------------------------------------

DENND1A.V1         GGCCATCCTGGCACATGGCTCTTCCTGGGTCAACCGTGACCTGTCTGGCTCAGGAATGGGCTCTGGCTGCTGGGGGAGCCGTGTCACTCCTGG
DENND1A.V3         GGCCATCCTGGCACATGGCTCTTCCTGGGTCAACCGTGACCTGTCTGGCTCAGGAATGGGCTCTGGCTGCTGGGGGAGCCGTGTCACTCCTGG
DENND1A.V4         GGCCATCCTGGCACATGGCTCTTCCTGGGTCAACCGTGACCTGTCTGGCTCAGGAATGGGCTCTGGCTGCTGGGGGAGCCGTGTCACTCCTGG
DENND1A.V1,3,4_F   ---------------------------------------------------------------------------------------------
DENND1A.V1,3,4_R   ---------------------------------------------------------------------------------------------

DENND1A.V1         GCCATGGGGGCACCTCCTGGGCACTTAGGTGTTTCAGCATAGATTCCAGTTTCGCACCCTGGGCAGACCCCCAGGCCCCATCCGGGATAGGGC
DENND1A.V3         GCCATGGGGGCACCTCCTGGGCACTTAGGTGTTTCAGCATAGATTCCAGTTTCGCACCCTGGGCAGACCCCCAGGCCCCATCCGGGATAGGGC
DENND1A.V4         GCCATGGGGGCACCTCCTGGGCACTTAGGTGTTTCAGCATAGATTCCAGTTTCGCACCCTGGGCAGACCCCCAGGCCCCATCCGGGATAGGGC
DENND1A.V1,3,4_F   ---------------------------------------------------------------------------------------------
DENND1A.V1,3,4_R   ---------------------------------------------------------------------------------------------

DENND1A.V1         AGAGGAGGTGCTGGCGGCCCCAGGGAAGGAGGGTGTGTACCCCAAGGCCCCCTGGCTGTGCTGAGGGGCTGGGGTGAGCGCTCCATGTTCACA
DENND1A.V3         AGAGGAGGTGCTGGCGGCCCCAGGGAAGGAGGGTGTGTACCCCAAGGCCCCCTGGCTGTGCTGAGGGGCTGGGGTGAGCGCTCCATGTTCACA
DENND1A.V4         AGAGGAGGTGCTGGCGGCCCCAGGGAAGGAGGGTGTGTACCCCAAGGCCCCCTGGCTGTGCTGAGGGGCTGGGGTGAGCGCTCCATGTTCACA
DENND1A.V1,3,4_F   ---------------------------------------------------------------------------------------------
DENND1A.V1,3,4_R   ---------------------------------------------------------------------------------------------

DENND1A.V1         TGAGCACTGCTGCCTCTTCACTTGTGGGACTTTTTGCAAACCCAAGGATGAACTTTGTGTGCATTCAATAAAATCATCTTGGGGAAGAGG
DENND1A.V3         TGAGCACTGCTGCCTCTTCACTTGTGGGACTTTTTGCAAACCCAAGGATGAACTTTGTGTGCATTCAATAAAATCATCTTGGGGAAGAGG
DENND1A.V4         TGAGCACTGCTGCCTCTTCACTTGTGGGACTTTTTGCAAACCCAAGGATGAACTTTGTGTGCATTCAATAAAATCATCTTGGGGAAGAGG
DENND1A.V1,3,4_F   ------------------------------------------------------------------------------------------
DENND1A.V1,3,4_R   ------------------------------------------------------------------------------------------
```

Exon 22
