# Supplementary material for: Could perturbed fetal development of the ovary contribute to the development of polycystic ovary syndrome in later life?
Source: PLoS One. 2020 Feb 20;15(2):e0229351. doi: 10.1371/journal.pone.0229351 (PMC7032716; doi:10.1371/journal.pone.0229351)
Supplement: S4 Fig — (PDF) [file pone.0229351.s004.pdf]

Fig S4. Alignment of human *DENND1A.V2*; bovine *DENND1A*; and predicted bovine *DENND1A.V2* primer sequences. Alignment was generated with CLUSTAL multiple sequence alignment by Kalign (2.0) based on sequences available at Ensembl Asia with transcript ID: human *DENND1A.V2* (ENST00000373620.7) and bovine *DENND1A* (ENSBTAG00000003610). Uppercase letters represent the exons, whereas lowercase letters represent the introns and only the sequence encompassing the exons is shown. Highlighted areas represent the primer sequences. .... represent areas of intronic sequence that are not illustrated in order to reduce the size of this file.

```
DENND1A.V2_HUMAN        CGCGCCGGGCACGCGCCGGCGACCATGGCGTTCGCCGGGCTGGAGCGAGTACATTAACCCCTGGAGGCGGCGGCGGC
DENND1A_BOVINE          -----------------------------------------------------------------------------------
DENND1A.V2_F1_BOVINE    -----------------------------------------------------------------------------------
DENND1A.V2_F2_BOVINE    -----------------------------------------------------------------------------------
DENND1A.V2_R_BOVINE     -----------------------------------------------------------------------------------

DENND1A.V2_HUMAN        GAGGGAGCGAGCCTCGAGCGGGCGGCCCCAGCCTGAGGGAAGGGAGGAAGGGGCGGGGAGAGCGCCAGAGGGAGGCCGGTCGG
DENND1A_BOVINE          -----------------------------------------------------------------GGCCGGTCGGCCGGTCGG
DENND1A.V2_F1_BOVINE    -----------------------------------------------------------------------------------
DENND1A.V2_F2_BOVINE    -----------------------------------------------------------------------------------
DENND1A.V2_R_BOVINE     -----------------------------------------------------------------------------------

DENND1A.V2_HUMAN        CCGCGGGCGGGCGGGCAGC----GCAGCGCCGAGCGGGGCCCGCGGGCCCATGAGGAGGCCTGGGGACCATGGGCTCCAGGAT
DENND1A_BOVINE          CCGCAGGCGGGCGGGCAGCGCAGCGCAGCGCCGAGCGGGGCCCGCGGGCCCATGAGGAGGCCGGGGGACCATGGGCTCCAGGAT
DENND1A.V2_F1_BOVINE    -----------------------------------------------------------------------------------
DENND1A.V2_F2_BOVINE    -----------------------------------------------------------------------------------
DENND1A.V2_R_BOVINE     -----------------------------------------------------------------------------------

DENND1A.V2_HUMAN        CAAgtgagtgcggccgggccgggcggagccgggccgggcgcggg............gtaatcatgatctgtttcttttgtag
DENND1A_BOVINE          CAAgtgagtgtggtcggcctgggcggagccgggccgggcgaggg............gtaatcacggtctgtttcttttgtag
DENND1A.V2_F1_BOVINE    -----------------------------------------------------------------------------------
DENND1A.V2_F2_BOVINE    -----------------------------------------------------------------------------------
DENND1A.V2_R_BOVINE     -----------------------------------------------------------------------------------
                                                                                                    Exon 1

DENND1A.V2_HUMAN        GCAGAATCCAGAGACCACATTTGAAGTATATGTTGAAGTGGCCTATCCCAGGACAGGTGGCACTCTTTCAGgta......tacag
DENND1A_BOVINE          GCAAAACCCAGAGACCACCTTTGAAGTGTATGTTGAAGTGGCCTGTCCTAGGACAGGTGGCACTCTTTCAGgta......tacag
DENND1A.V2_F1_BOVINE    -----------------------------------------------------------------------------------
DENND1A.V2_F2_BOVINE    -----------------------------------------------------------------------------------
DENND1A.V2_R_BOVINE     -----------------------------------------------------------------------------------
                                                                                                    Exon 2

DENND1A.V2_HUMAN        ATCCTGAGGTGCAGAGGCAATTCCCGGAGGACTACAGTGACCAGgttcggaatgcgtaatt.....atctctctttctctccacag
DENND1A_BOVINE          ATCCTGAGGTGCAGAGGCAATTCCCGGAGGACTACAGTGACCAGgttcggaatgcgtaatt.....atctctctttctctccacag
DENND1A.V2_F1_BOVINE    -----------------------------------------------------------------------------------
DENND1A.V2_F2_BOVINE    -----------------------------------------------------------------------------------
DENND1A.V2_R_BOVINE     -----------------------------------------------------------------------------------
                                                                                                    Exon 3

DENND1A.V2_HUMAN        GAAGTTCTACAGACTTTGACCAAGTTTTGTTTCCCCTTCTATGTGGACAGgtagtgtcag........gttctgcctttgctgcag
DENND1A_BOVINE          GAAGTTCTACAGACTCTGACCAAGTTTTGTTTCCCCTTCTATGTGGACAGgtagtgtcag........tgttct--tttggcgcag
DENND1A.V2_F1_BOVINE    -----------------------------------------------------------------------------------
DENND1A.V2_F2_BOVINE    -----------------------------------------------------------------------------------
DENND1A.V2_R_BOVINE     -----------------------------------------------------------------------------------
                                                                                                    Exon 4

DENND1A.V2_HUMAN        CCTCACAGTTAGCCAAGTTGGCCAGAACTTCACATTCGTGCTCACTGACATTGACAGCAAACAGAGATTCGGGTTCTGCCGCTT
DENND1A_BOVINE          CCTCACAGTTAGCCAAGTTGGCCAGAACTTCACATTCGTGCTCACTGACATTGACAGCAAACAGAGATTCGGGTTCTGCCGCTT
DENND1A.V2_F1_BOVINE    -----------------------------------------------------------------------------------
DENND1A.V2_F2_BOVINE    -----------------------------------------------------------------------------------
DENND1A.V2_R_BOVINE     -----------------------------------------------------------------------------------

DENND1A.V2_HUMAN        ATCTTCAGGAGCGAAGAGCTGCTTCTGTATCTTAAGgtaagggagaaggcttgggctg..........tttcctcttcttcctccag
DENND1A_BOVINE          ATCTTCAGGAGCAAAGAGTTGCTTCTGTATCTTAAGgtaagggagaaggcttgggctg.........   .tttcctcttcttcctccag
DENND1A.V2_F1_BOVINE    -----------------------------------------------------------------------------------
DENND1A.V2_F2_BOVINE    -----------------------------------------------------------------------------------
DENND1A.V2_R_BOVINE     -----------------------------------------------------------------------------------
                                                                                                    Exon 5

DENND1A.V2_HUMAN        CTATCTCCCCTGGTTCGAGGTATTTTATAAGCTGCTTAACATCCTGGCAGATTACACGACAAAAAGACAGgtat......ttcag
DENND1A_BOVINE          CTATCTCCCCTGGTTCGAAGTATTTTATAAGCTACTTAACATCTTGGCAGATTACACGACAAAAGGACAGgtat......ttcag
DENND1A.V2_F1_BOVINE    -----------------------------------------------------------------------------------
DENND1A.V2_F2_BOVINE    -----------------------------------------------------------------------------------
DENND1A.V2_R_BOVINE     -----------------------------------------------------------------------------------
                                                                                                    Exon 6
```

Exon 7

```
DENND1A.V2_HUMAN        GAAAATCAGTGGAATGAGCTTCTTGAAACTCTGCACAAACTTCCCATCCCTGACCCAGGAGTGTCTGTCCATCTCAGCGTGgta
DENND1A_BOVINE          GAGAGTCAGTGGAATGAGCTTCTTGAAACTCTGTACAAACTTCCTATCCCTGACCCAGGAGTGTCTGTTCATCTCAGTGTGgta
DENND1A.V2_F1_BOVINE    ------------------------------------------------------------------------------------
DENND1A.V2_F2_BOVINE    ------------------------------------------------------------------------------------
DENND1A.V2_R_BOVINE     ------------------------------------------------------------------------------------

DENND1A.V2_HUMAN        agtgggg-cggagtattagccatcactgaaaacg……………………attttagcagaaataaatcacttttgcttttcttttctag
DENND1A_BOVINE          agtgggggtagaatattagcaatcactgagaaac……………………actttttcaggaataaat-atttttatcttcctttacag
DENND1A.V2_F1_BOVINE    ------------------------------------------------------------------------------------
DENND1A.V2_F2_BOVINE    ------------------------------------------------------------------------------------
DENND1A.V2_R_BOVINE     ------------------------------------------------------------------------------------
```

Exon 8

```
DENND1A.V2_HUMAN        CATTCTTATTTTACTGTGCCTGATACCAGAGAACTTCCCAGCATACCTGAGAATgtaagtactt…………tcttctttctcctag
DENND1A_BOVINE          CATTCTTATTTTACTGTGCCTGATACCAGAGAACTTCCCAGCATCCCTGAGAATgtaagtactt…………tcttctttctcctag
DENND1A.V2_F1_BOVINE    ------------------------------------------------------------------------------------
DENND1A.V2_F2_BOVINE    ------------------------------------------------------------------------------------
DENND1A.V2_R_BOVINE     ------------------------------------------------------------------------------------
```

Exon 9

```
DENND1A.V2_HUMAN        AGAAATCTGACAGAATATTTTGTGGCTGTGGATGTTAACAACATGTTGCATCTGTACGCCAGTATGCTGTACGAACGCCGGATA
DENND1A_BOVINE          AGAAATCTGACAGAATATTTTGTGGCTGTGGATGTAAACAACATGTTACATCTGTATGCCAGTATGCTCTACGAACGCCGGATA
DENND1A.V2_F1_BOVINE    ------------------------------------------------------------------------------------
DENND1A.V2_F2_BOVINE    ------------------------------------------------------------------------------------
DENND1A.V2_R_BOVINE     ------------------------------------------------------------------------------------

DENND1A.V2_HUMAN        CTCATCATTTGCAGCAAACTCAGCACTgtgagtagacagtc……………………………tcattgatctctgctttgctctgttccag
DENND1A_BOVINE          CTCATCATTTGCAGCAAACTCAGCACTgtgagtagacagtc……………………………tagctctgaatctttaatctctgttgcag
DENND1A.V2_F1_BOVINE    ------------------------------------------------------------------------------------
DENND1A.V2_F2_BOVINE    ------------------------------------------------------------------------------------
DENND1A.V2_R_BOVINE     ------------------------------------------------------------------------------------
```

Exon 10

```
DENND1A.V2_HUMAN        CTGACTGCCTGCATCCACGGGTCTGCGGCGATGCTCTACCCCATGTACTGGCAGCACGTGTACATCCCCGTGCTGCCGCCGCAT
DENND1A_BOVINE          TTGACTGCCTGCATCCACGGGTCTGCTGCGATGCTCTACCCCATGTTCTGGCAGCACGTGTACATCCCTGTCCTGCCTCCACAT
DENND1A.V2_F1_BOVINE    ------------------------------------------------------------------------------------
DENND1A.V2_F2_BOVINE    ------------------------------------------------------------------------------------
DENND1A.V2_R_BOVINE     ------------------------------------------------------------------------------------

DENND1A.V2_HUMAN        CTGCTGGACTACTGCTGgtaaggtggctg---gccctg………………acattaaagcagctgtgtgtctctgtttctcttcacag
DENND1A_BOVINE          CTGCTGGACTACTGCTGgtaaggccactgcccaccctg………………acattaaagcagctgtgtgtctctgtttctcttcacag
DENND1A.V2_F1_BOVINE    ------------------------------------------------------------------------------------
DENND1A.V2_F2_BOVINE    ------------------------------------------------------------------------------------
DENND1A.V2_R_BOVINE     ------------------------------------------------------------------------------------
```

Exon 11

```
DENND1A.V2_HUMAN        TGCTCCCATGCCCTACCTCATAGGAATCCATTTAAGTTTAATGGAGgtaagttggc…………………tttttt--tctccttgcag
DENND1A_BOVINE          TGCTCCCATGCCCTACCTCATAGGAATCCATTTAAGTTTAATGGAGgtaagttgac…………………ttttttcttttacttgcag
DENND1A.V2_F1_BOVINE    ------------------------------------------------------------------------------------
DENND1A.V2_F2_BOVINE    ------------------------------------------------------------------------------------
DENND1A.V2_R_BOVINE     ------------------------------------------------------------------------------------
```

Exon 12

```
DENND1A.V2_HUMAN        AAAGTCAGAAACATGGCCCTGGATGATGTCGTGATCCTGAATGTGGACACCAACACCCTGGAAACCCCCTTCGATGACCTCCAG
DENND1A_BOVINE          AAAGTCAGAAGCATGGCCCTGGATGATGTCGTGATCCTGAATGTGGACACCAACACCCTGGAAACCCCCTTTGATGACCTCCAG
DENND1A.V2_F1_BOVINE    ------------------------------------------------------------------------------------
DENND1A.V2_F2_BOVINE    ------------------------------------------------------------------------------------
DENND1A.V2_R_BOVINE     ------------------------------------------------------------------------------------

DENND1A.V2_HUMAN        AGCCTCCCAAACGACGTGgtaggtaatgagcttgcgaggatctcac……………………ctgtgttttccttctctccatcaccag
DENND1A_BOVINE          AGCCTCCCAAATGATGTGgtgggtaatgagctctcgagt-tctgac……………………ctgtat----ctcctttctgtcatcag
DENND1A.V2_F1_BOVINE    ------------------------------------------------------------------------------------
DENND1A.V2_F2_BOVINE    ------------------------------------------------------------------------------------
DENND1A.V2_R_BOVINE     ------------------------------------------------------------------------------------
```

Exon 13

```
DENND1A.V2_HUMAN        ATCTCTTCCCTGAAGAACAGGCTGAAAAAGGTCTCCACAACCACTGGGGATGGTGTGGCCAGAGCGTTCCTCAAGGCCCAGGCT
DENND1A_BOVINE          ATCTCTTCCTTGAAGAGCCGGCTGAAGAAGGTGTCCACGACAACTGGTGATGGTGTGGCCAGAGCCTTCCTCAAGGCCCAGGCC
DENND1A.V2_F1_BOVINE    ------------------------------------------------------------------------------------
DENND1A.V2_F2_BOVINE    ------------------------------------------------------------------------------------
DENND1A.V2_R_BOVINE     ------------------------------------------------------------------------------------

DENND1A.V2_HUMAN        GCTTTCTTCGGTAGCTACCGAAACGCTCTGAAAATCGAGCCGgtgagtagc—c………………ccc------tctgcacttcccag
DENND1A_BOVINE          GCTTTCTTCGGCAGCTACCGAAACGCTCTGAAAATCGAGCCGgtgagtagctt………………tccatcttatctctgtctcccag
DENND1A.V2_F1_BOVINE    ------------------------------------------------------------------------------------
DENND1A.V2_F2_BOVINE    ------------------------------------------------------------------------------------
DENND1A.V2_R_BOVINE     ------------------------------------------------------------------------------------
```

Exon 14

```
DENND1A.V2_HUMAN      GAGGAGCCGATCACTTTCTGTGAGGAAGCCTTCGTGTCCCACTACCGCTCCGGAGCCATGAGGCAGTTCCTGCAGAACGCCACA
DENND1A_BOVINE        GAGGAGCCAATCACCTTCTGCGAGGAAGCCTTCGTGTCGCACTATCGCTCAGGAGCCATGAGGCAGTTCCTGCAGAATGCCACC
DENND1A.V2_F1_BOVINE  ------------------------------------------------------------------------------------
DENND1A.V2_F2_BOVINE  ------------------------------------------------------------------------------------
DENND1A.V2_R_BOVINE   ------------------------------------------------------------------------------------

DENND1A.V2_HUMAN      CAGCTGCAGCTCTTCAAGCAGgtgcctc-cctccctggtctggcctgggtctgg..........tttgttccttctttctatag
DENND1A_BOVINE        CAGCTGCAGCTCTTTAAACAGgtgcccagcctcccttgcctggtctgcgtctgg..........tttgttctttctttgtatag
DENND1A.V2_F1_BOVINE  ------------------------------------------------------------------------------------
DENND1A.V2_F2_BOVINE  ------------------------------------------------------------------------------------
DENND1A.V2_R_BOVINE   ------------------------------------------------------------------------------------
```

Exon 15

```
DENND1A.V2_HUMAN      TTTATTGATGGTCGATTAGATCTTCTCAATTCCGGCGAAGGTTTCAGTGATGTTTTTGAAGAGGAAATCAACATGGGCGAGTAC
DENND1A_BOVINE        TTTATCGATGGTAGATTAGACCTTCTCAATTCTGGCGAGGGCTTCAGTGATGTTTTTGAAGAGGAGATCAACATGAGCGAGTAT
DENND1A.V2_F1_BOVINE  ------------------------------------------------------------------------------------
DENND1A.V2_F2_BOVINE  ------------------------------------------------------------------------------------
DENND1A.V2_R_BOVINE   ------------------------------------------------------------------------------------

DENND1A.V2_HUMAN      GCTGgtgagaagcaac.........................................gtgacagcttctctgaattctctttccccag
DENND1A_BOVINE        GCTGgtaag-ggcagt.........................................ataccagcttctctgcctcctctttccccag
DENND1A.V2_F1_BOVINE  ------------------------------------------------------------------------------------
DENND1A.V2_F2_BOVINE  ------------------------------------------------------------------------------------
DENND1A.V2_R_BOVINE   ------------------------------------------------------------------------------------
```

Exon 16

```
DENND1A.V2_HUMAN      GCAGTGACAAACTGTACCATCAGTGGCTCTCCACTGTCCGGgtaagcatgcacccaat...........acattttattatag
DENND1A_BOVINE        GGAGTGATAAGCTATACCACCAGTGGCTCTCCACAGTCCGGgtaag--tgccacccc..........  .acattttattatag
DENND1A.V2_F1_BOVINE  ------------------------------------------------------------------------------------
DENND1A.V2_F2_BOVINE  ------------------------------------------------------------------------------------
DENND1A.V2_R_BOVINE   ------------------------------------------------------------------------------------
```

Exon 17

```
DENND1A.V2_HUMAN      AAAGGAAGTGGAGCAATTCTGAATACTGTAAAGACCAAAGCAAATCCGGCCATGAAGACTGTCTACAAGTTCgtaa...tgcag
DENND1A_BOVINE        AAAGGAAGTGGAGCAATTTTGAATACTGTAAAAACGAAAGCAAACCCGGCCATGAAGACTGTCTATAAGTTCgtaa...tgcag
DENND1A.V2_F1_BOVINE  ------------------------------------------------------------------------------------
DENND1A.V2_F2_BOVINE  ------------------------------------------------------------------------------------
DENND1A.V2_R_BOVINE   ------------------------------------------------------------------------------------
```

Exon 18

```
DENND1A.V2_HUMAN      GCAAAAGATCATGCAAAAATGGGAATAAAAGAGGTGAAAAACCGCTTGAAGCAAAAGgtacttgaagtt....tctccctacag
DENND1A_BOVINE        GCAAAAGATCATGCAAAAATGGGAATAAAAGAGGTGAAAAACCGCTTGAAGCAAAAGgtacttgaagtt....tctccttgcag
DENND1A.V2_F1_BOVINE  ------------------------------------------------------------------------------------
DENND1A.V2_F2_BOVINE  ------------------------------------------------------------------------------------
DENND1A.V2_R_BOVINE   ------------------------------------------------------------------------------------
```

Exon 19

```
DENND1A.V2_HUMAN      GACATTGCCGAGAATGGCTGCGCCCCCACCCCAGAAGAGCAGCTGCCAAAGACTGCACCGTCCCCACTGGTGGAGGCCAAGGAC
DENND1A_BOVINE        GACATCACTGAGAATGGCTGTGCCCCCACCACAGAAGAGCAGCTGCCAAAGACTGTGCCGTCCCCACTGGTAGAGGCCAAGGAC
DENND1A.V2_F1_BOVINE  ------------------------------------------------------------------------------------
DENND1A.V2_F2_BOVINE  ------------------------------------------------------------------------------------
DENND1A.V2_R_BOVINE   ------------------------------------------------------------------------------------

DENND1A.V2_HUMAN      CCCAAGCTCCGAGAAGACCGGCGGCCAATCACAGTCCACTTTGGACAGgtgtgtaccctggcc....ccctgtacctcctctag
DENND1A_BOVINE        CCCAAGTTCCGAGAGGACCGGCGGCCAATCACAGTCCACTTTGGACAGgtatgtacctggccc....ccc-atgcccattctag
DENND1A.V2_F1_BOVINE  ------------------------------------------------------------------------------------
DENND1A.V2_F2_BOVINE  ------------------------------------------------------------------------------------
DENND1A.V2_R_BOVINE   ------------------------------------------------------------------------------------
```

Exon 20

```
DENND1A.V2_HUMAN      GTGCGCCCACCTCGTCCACATGTTGTTAAGAGACCAAAGAGCAACATCGCAGTGGAAGGCCGGAGGACGTCTGTGCCGAGCCCT
DENND1A_BOVINE        GTGCGCCCTCCCCGTCCACACGTCGTTAAAAGACCGAAGAGCAACATCACAGTGGAAGCCCGAAGGACGTCCGTCTCGAGCCCC
DENND1A.V2_F1_BOVINE  --------------------------------ACCGAAGAGCAACATCACAG--------------------------------
DENND1A.V2_F2_BOVINE  ---------------------------------------AGCAACATCACAGTGGAAGC-------------------------
DENND1A.V2_R_BOVINE   ------------------------------------------------------------------------------------

DENND1A.V2_HUMAN      GAGCAgtgagtattgtgcctctcccctctgtctgtaagaac.................ccagaatattcttgcattaca-cag
DENND1A_BOVINE        GACCAgtgagtaccccgtcctcccctcccctctgcaggagc................ .ccagaatatccttgcattgtaccag
DENND1A.V2_F1_BOVINE  ------------------------------------------------------------------------------------
DENND1A.V2_F2_BOVINE  ------------------------------------------------------------------------------------
DENND1A.V2_R_BOVINE   ------------------------------------------------------------------------------------
```

Exon 21

```
DENND1A.V2_HUMAN      AAACACCATTGCAACACCAGCTACACTCCACATCCTACAGAAAAGCATTACCCATTTTGCGGCCAAGTTCCCGACGAGAGGCTG
DENND1A_BOVINE        aaaca------------caccaacacttgccagtctaccgaaaagcattgcccat-ttgtggccaagtccccgattagagcttg
DENND1A.V2_F1_BOVINE  ------------------------------------------------------------------------------------
DENND1A.V2_F2_BOVINE  ------------------------------------------------------------------------------------
DENND1A.V2_R_BOVINE   ------------------------------------------------------------------------------------
```

```
DENND1A.V2_HUMAN        GACCTCTTCATCACATTGACTTACGCCGTTGCTTTTCCAGACTGGGCAGAGGGGCTGACTTCG-CAGTGTGTGCCAAAGAGCCG
DENND1A_BOVINE          Gacctccccaccacacgtgtttatgctgttgcttttccagccccgtgataggagattgctgcgtcagtgtatg-caaagagcca
DENND1A.V2_F1_BOVINE    ------------------------------------------------------------------------------------
DENND1A.V2_F2_BOVINE    ------------------------------------------------------------------------------------
DENND1A.V2_R_BOVINE     ----------------------------------------------GATAGGAGATTGCTGCGTCA------------------

DENND1A.V2_HUMAN        GTGTCTGATAATCCCATTTTCCTGCTTATCACCTGAACTGTGTCAGTATCACTTTTAGTTTTGTTGGTTGGTTGGTTTGTTGTT
DENND1A_BOVINE          gggtctggcacccccatcattctggtggttaccagagctgggtcggtaccactttg--------tgcgggttttgttttgtttt
DENND1A.V2_F1_BOVINE    ------------------------------------------------------------------------------------
DENND1A.V2_F2_BOVINE    ------------------------------------------------------------------------------------
DENND1A.V2_R_BOVINE     ------------------------------------------------------------------------------------

DENND1A.V2_HUMAN        TGTTTAATATGCCCTGTTTTCTACTTCTGTTGGA-AAATATTTGGGGTTGAAATAAACCAGTGGGAGCATGGG-AGCCAGTTTG
DENND1A_BOVINE          attgcattatgccttgttatggatttctttgggggaaacatttgaggttgaaatgaaccggtgggagcagggttggctagttta
DENND1A.V2_F1_BOVINE    ------------------------------------------------------------------------------------
DENND1A.V2_F2_BOVINE    ------------------------------------------------------------------------------------
DENND1A.V2_R_BOVINE     ------------------------------------------------------------------------------------

DENND1A.V2_HUMAN        GTGG-TTGGCAAACTACCAGTGATAGAAAACAAATGACAGGTTTTCTGTGAGCGTACGTCACAGTGGCTCG---------GGCC
DENND1A_BOVINE          Gtgggctggcacactggcagttatagaagacaaatgacaggttctctgtgatcacgcataacagtgctccaccgtacagtggcc
DENND1A.V2_F1_BOVINE    ------------------------------------------------------------------------------------
DENND1A.V2_F2_BOVINE    ------------------------------------------------------------------------------------
DENND1A.V2_R_BOVINE     ------------------------------------------------------------------------------------

DENND1A.V2_HUMAN        CACAAGGAGACAGAGGGGTACGTTTCAGGACATCATTCCAAGGGTTTCTGTTCACGTTTGTTTAAC-----------GAGGAAG
DENND1A_BOVINE          caccaggagatagggaggtacatttagaacatcattccaagagtttctgttcacttctttttttttttatggggtgagggaag
DENND1A.V2_F1_BOVINE    ------------------------------------------------------------------------------------
DENND1A.V2_F2_BOVINE    ------------------------------------------------------------------------------------
DENND1A.V2_R_BOVINE     ------------------------------------------------------------------------------------

DENND1A.V2_HUMAN        GGGAAGTTGTTCTCCCATAATACCAGTGGTATTTCTAGTGCAACCCGGGTGTTTTTCTACGCCTCTTCCCATGCTGCTTCCCCA
DENND1A_BOVINE          gcagggctgtattcctataacgccagtgacatttctggtgtaacccgggtgtttccctgcgtctcttcccatgctgccttctcc
DENND1A.V2_F1_BOVINE    ------------------------------------------------------------------------------------
DENND1A.V2_F2_BOVINE    ------------------------------------------------------------------------------------
DENND1A.V2_R_BOVINE     ------------------------------------------------------------------------------------

DENND1A.V2_HUMAN        C-----CCCCCCACTGTGCTCCGCCCCTTCCAAATGCCATGTCACAACAGCACTTGGATGTGTTTTTCTCAACTGTCATCAGCT
DENND1A_BOVINE          ctgcccccacccccctgttgtgctccacttccaaatgccatgtcacaacagcacttggatgtgtttttctcaactgtcatcagct
DENND1A.V2_F1_BOVINE    ------------------------------------------------------------------------------------
DENND1A.V2_F2_BOVINE    ------------------------------------------------------------------------------------
DENND1A.V2_R_BOVINE     ------------------------------------------------------------------------------------

DENND1A.V2_HUMAN        CCAGCTGGCAGGACCAACTTCTTGAAACACAGGAAGCATCCAGCGGAAAATATTTTAATAAAACAGACTCCTCATAAAATATT
DENND1A_BOVINE          ccagctggcaagaccaacttcttgaaacacaggaagcatccagcggaaaatattttaataaaacagactcctcataaaatgtt
DENND1A.V2_F1_BOVINE    ------------------------------------------------------------------------------------
DENND1A.V2_F2_BOVINE    ------------------------------------------------------------------------------------
DENND1A.V2_R_BOVINE     ------------------------------------------------------------------------------------

DENND1A.V2_HUMAN        GTTCGGGGGAGGGGGAAGAAACCGGCTC-CACCAATCTGTCGGCATTGTATTGGGAGATGTGAGAAGCCAGGCTGGCAG-CAGG
DENND1A_BOVINE          gttttgggg---ggaagaacccgcccctccccagtctgtcagcattgtgtcgggagacacgagaagacaagccggcgggcagg
DENND1A.V2_F1_BOVINE    ------------------------------------------------------------------------------------
DENND1A.V2_F2_BOVINE    ------------------------------------------------------------------------------------
DENND1A.V2_R_BOVINE     ------------------------------------------------------------------------------------

DENND1A.V2_HUMAN        GGCCCGATCCTAATGGGCCTTTGTCAGGTGGTGGTTCCGTGTTGGGCACACGGACTTGGTGCCTGGCCCTTTAATCTGGCCTGG
DENND1A_BOVINE          agccacatcctaatggggctttgttgcg-ggtggttcagtgctgggcacgcggactcggtgcctggccctttaatctggcctgg
DENND1A.V2_F1_BOVINE    ------------------------------------------------------------------------------------
DENND1A.V2_F2_BOVINE    ------------------------------------------------------------------------------------
DENND1A.V2_R_BOVINE     ------------------------------------------------------------------------------------

DENND1A.V2_HUMAN        GTTTTCTTGAATCACAGATCTTCATCTCCACTGGGAATATCAAAGTGCTTGGGAAACTTAGAAGACTGG-GGCAGCAGAAGGGG
DENND1A_BOVINE          attctcttgaatcaca-atctttgtctccgctgggaatatcagagtgcatcagaaacttggaggatttttggtggcagaaggag
DENND1A.V2_F1_BOVINE    ------------------------------------------------------------------------------------
DENND1A.V2_F2_BOVINE    ------------------------------------------------------------------------------------
DENND1A.V2_R_BOVINE     ------------------------------------------------------------------------------------

DENND1A.V2_HUMAN        ATGCAGGATCTTTGAAGTGGAGAGGAGAGATTCATTCCTCCTGACTTCTTGCCCCAACTCTCACTTCCAATT-GCACATTAAAT
DENND1A_BOVINE          acacaggatctttgaagtggag-ggcgggattcggtcctcctgacttcttgccccaactttcacttccaaaaagcacattaaat
DENND1A.V2_F1_BOVINE    ------------------------------------------------------------------------------------
DENND1A.V2_F2_BOVINE    ------------------------------------------------------------------------------------
DENND1A.V2_R_BOVINE     ------------------------------------------------------------------------------------
```

Exon 21

```
DENND1A.V2_HUMAN        AAT-------------------------CCCAGCACT---------------------------TTGGGAGGCCAAGGCGAGCG
DENND1A_BOVINE          aattaactg..........ctggcactgccgcagcactgctggccact..........attgaacagatggggaaactgaggctcgga
DENND1A.V2_F1_BOVINE    ------------------------------------------------------------------------------------
DENND1A.V2_F2_BOVINE    ------------------------------------------------------------------------------------
DENND1A.V2_R_BOVINE     ------------------------------------------------------------------------------------

DENND1A.V2_HUMAN        GA--------------TCACCTGAGGTCAGGGGTTCGAGA----------------------------CCAGCCTGGGCAACA
DENND1A_BOVINE          gagaggctttccataacttgcccaaggtctgggggctggaaaatggagagat..........atcttccctccggcttgctagata
DENND1A.V2_F1_BOVINE    ------------------------------------------------------------------------------------
DENND1A.V2_F2_BOVINE    ------------------------------------------------------------------------------------
DENND1A.V2_R_BOVINE     ------------------------------------------------------------------------------------

DENND1A.V2_HUMAN        TGGCGAAACCCCGTCTCTACT----------AAAAATACAAAAATTAGCCGAGTGTGGTGGCGCACCCCTGTAATTCCAGCTA
DENND1A_BOVINE          tggggcaatttcgcatccaacctaaggcttagaaagagtcgatagcaagctgtaaaaggcagcactgctcagtactgcccgccc
DENND1A.V2_F1_BOVINE    ------------------------------------------------------------------------------------
DENND1A.V2_F2_BOVINE    ------------------------------------------------------------------------------------
DENND1A.V2_R_BOVINE     ------------------------------------------------------------------------------------

DENND1A.V2_HUMAN        CTCGGGA---------------------------GGCTGAGGCATGAGAATCACTT---------------------------G
DENND1A_BOVINE          ctcaggaccacacacag..........actgtcctcaggatgagggatgggtgtttctcagcatctaca..........aaggcacaacg

DENND1A.V2_F1_BOVINE    ------------------------------------------------------------------------------------
DENND1A.V2_F2_BOVINE    ------------------------------------------------------------------------------------
DENND1A.V2_R_BOVINE     ------------------------------------------------------------------------------------

DENND1A.V2_HUMAN        AACCCAGGAGGCGGAGG--------------------------------------------------CTGCCGTGAGCTGAGAT
DENND1A_BOVINE          aacccaggagatggagatgagggcagtggagccagtaagtgccccgtgccggctccccaggccaccaccgccccaccccgacct
DENND1A.V2_F1_BOVINE    ------------------------------------------------------------------------------------
DENND1A.V2_F2_BOVINE    ------------------------------------------------------------------------------------
DENND1A.V2_R_BOVINE     ------------------------------------------------------------------------------------

DENND1A.V2_HUMAN        CACACCACCACACTCCAGCC---------------------------------------TGTGCAACAGAGTGAGACTCTGTCT
DENND1A_BOVINE          cacaccacctggtccctgccaaccccacctgccaccctcaa..........tattcgtccttgaacacccaggggagactctgaac
DENND1A.V2_F1_BOVINE    ------------------------------------------------------------------------------------
DENND1A.V2_F2_BOVINE    ------------------------------------------------------------------------------------
DENND1A.V2_R_BOVINE     ------------------------------------------------------------------------------------

DENND1A.V2_HUMAN        CAAAAATAAAATAACCAGCTTTGTGGACAG-----------------------------CAAGATGGGGCTGATT---------
DENND1A_BOVINE          agggtggagaggtgcaaaatctgtggacaggtgagcgtag..........ggaatgcattcaagatggatttgagcgtcaccaaa
DENND1A.V2_F1_BOVINE    ------------------------------------------------------------------------------------
DENND1A.V2_F2_BOVINE    ------------------------------------------------------------------------------------
DENND1A.V2_R_BOVINE     ------------------------------------------------------------------------------------

DENND1A.V2_HUMAN        ------------------AAGAGTAAGACTGTCTCCTGGAGTAGCTG-------------------------AGCATCCTGGG
DENND1A_BOVINE          g..........agaggactggaaggagagggctctgaactggagtccctgctcatcacct..........agctgaggaaaggatcatgtg
DENND1A.V2_F1_BOVINE    ------------------------------------------------------------------------------------
DENND1A.V2_F2_BOVINE    ------------------------------------------------------------------------------------
DENND1A.V2_R_BOVINE     ------------------------------------------------------------------------------------

DENND1A.V2_HUMAN        ACTCATCAGGGCCCAGAAA----------------------------------CACTAGCCAGCCACTCTCTGGCCAGTCC---
DENND1A_BOVINE          Cctggtgggtgtccagaaagagggataaaatgggcgcgtgcgaggcctcgtctccccagcacgctggcttctgctcagtccctc
DENND1A.V2_F1_BOVINE    ------------------------------------------------------------------------------------
DENND1A.V2_F2_BOVINE    ------------------------------------------------------------------------------------
DENND1A.V2_R_BOVINE     ------------------------------------------------------------------------------------

DENND1A.V2_HUMAN        ------------------------ACCCCTGAAGTTCATTTCTTCCTTCCC-------------------------AGCTGATG
DENND1A_BOVINE          agcagac..........gccagggcacattcctgcgggtcagaccttcctgccctctctcctga..........ccagggacagctgctg
DENND1A.V2_F1_BOVINE    ------------------------------------------------------------------------------------
DENND1A.V2_F2_BOVINE    ------------------------------------------------------------------------------------
DENND1A.V2_R_BOVINE     ------------------------------------------------------------------------------------

DENND1A.V2_HUMAN        TCCATCCCT----------------------------CCATCATTCCATCTGTCCATTCGTGCATTCATTCATT---------
DENND1A_BOVINE          Tctctcacttaccctccag...........atttagacacctgcctttccagtctttttctatgcatttttccattagcaaatgag
DENND1A.V2_F1_BOVINE    ------------------------------------------------------------------------------------
DENND1A.V2_F2_BOVINE    ------------------------------------------------------------------------------------
DENND1A.V2_R_BOVINE     ------------------------------------------------------------------------------------

DENND1A.V2_HUMAN        -CAGCAAGTATCTTTGAAAA----------------------------CCATCATTCCATCTGTCCATTCGTGCATTCATTCAT
DENND1A_BOVINE          acagaaggtttttataaacatagtttaatg..........atttagacacctgcctttccagtctttttctatgcatttttccat
DENND1A.V2_F1_BOVINE    ------------------------------------------------------------------------------------
DENND1A.V2_F2_BOVINE    ------------------------------------------------------------------------------------
DENND1A.V2_R_BOVINE     ------------------------------------------------------------------------------------
```

Exon 21

```
DENND1A.V2_HUMAN        T-----------CAGCAAGTATCTTTGAAAA----------------------------CCTACTAGGCGCAAGCACGG-----
DENND1A_BOVINE          tagcaaatgagacagaaggttttataaacatagtttaatg…………ggtctccttcctcacagggagaatgcactggactg
DENND1A.V2_F1_BOVINE    ------------------------------------------------------------------------------------
DENND1A.V2_F2_BOVINE    ------------------------------------------------------------------------------------
DENND1A.V2_R_BOVINE     ------------------------------------------------------------------------------------

DENND1A.V2_HUMAN        ---------------------ACGATTTTTTTAAAGGAGGCCTTAAAAGGGAG--------------------TCCCCATGCTG
DENND1A_BOVINE          ttact…………atctgtttgcatggtgtttctaatgggagccttcaggggaagggaactgcccagaggggctctccccttgctc
DENND1A.V2_F1_BOVINE    ------------------------------------------------------------------------------------
DENND1A.V2_F2_BOVINE    ------------------------------------------------------------------------------------
DENND1A.V2_R_BOVINE     ------------------------------------------------------------------------------------

DENND1A.V2_HUMAN        ACTCTTGGTTGAC-----------------------------TTGCATTTTCCCAAAATGAAATTTTCC----TGTGTCAAATT
DENND1A_BOVINE          cccccttgtcagccccccttcact…………aaaataaagattgcatcattggaataagaaatgtccagccatgtatcaactt
DENND1A.V2_F1_BOVINE    ------------------------------------------------------------------------------------
DENND1A.V2_F2_BOVINE    ------------------------------------------------------------------------------------
DENND1A.V2_R_BOVINE     ------------------------------------------------------------------------------------

DENND1A.V2_HUMAN        -------------------TTCTTTCTGAATCAAT-------------------------------------------------
DENND1A_BOVINE          ac…………gctggccacgttctttttaaattaattgatgcctta…………tccagcctgctctgcctccccccagGCCACA
DENND1A.V2_F1_BOVINE    ------------------------------------------------------------------------------------
DENND1A.V2_F2_BOVINE    ------------------------------------------------------------------------------------
DENND1A.V2_R_BOVINE     ------------------------------------------------------------------------------------

DENND1A.V2_HUMAN        ------------------------------------------------------------------------------------
DENND1A_BOVINE          GCCATATCGGACACTCAAGGAGTCAGACAGTGCAGGGGACGAGGCCGAAAGCCCGGAGCAGCGAGCGCGGGAGCCTGTGGGCCC
DENND1A.V2_F1_BOVINE    ------------------------------------------------------------------------------------
DENND1A.V2_F2_BOVINE    ------------------------------------------------------------------------------------
DENND1A.V2_R_BOVINE     ------------------------------------------------------------------------------------

DENND1A.V2_HUMAN        ------------------------------------------------------------------------------------
DENND1A_BOVINE          CACCCCAGCTCCACACGACCGGGCCGCCAGCATCAACCTCCTGGAGGATGTCTTCAGCAACCTCGACATGGAAGTCCCGCTGCA
DENND1A.V2_F1_BOVINE    ------------------------------------------------------------------------------------
DENND1A.V2_F2_BOVINE    ------------------------------------------------------------------------------------
DENND1A.V2_R_BOVINE     ------------------------------------------------------------------------------------

DENND1A.V2_HUMAN        ------------------------------------------------------------------------------------
DENND1A_BOVINE          GCAGCTGGGCCAGGCCAAAAGCTTGGAAGACCTTCGGACCCCCAAAGACCTGAGGGAGCAGCCGGGGACCTTTGACTATCAGGt
DENND1A.V2_F1_BOVINE    ------------------------------------------------------------------------------------
DENND1A.V2_F2_BOVINE    ------------------------------------------------------------------------------------
DENND1A.V2_R_BOVINE     ------------------------------------------------------------------------------------

DENND1A.V2_HUMAN        --------------------------GATAGGTTTCCTAGTCCCTTA----------------------------AAACTGGCC
DENND1A_BOVINE          atggtggt…………gggagaaagagccacttttccaagtccctcagccagtgctc…………gcctgatgaaaatctgccc
DENND1A.V2_F1_BOVINE    ------------------------------------------------------------------------------------
DENND1A.V2_F2_BOVINE    ------------------------------------------------------------------------------------
DENND1A.V2_R_BOVINE     ------------------------------------------------------------------------------------

DENND1A.V2_HUMAN        GTTG----------------------------GCAATTGGAAGA----------------------------------------
DENND1A_BOVINE          gctgcttcatgggc…………tcgtggcccagcaggtggcagagctggacaga…………tctccggcagAGGCTGGACCTG
DENND1A.V2_F1_BOVINE    ------------------------------------------------------------------------------------
DENND1A.V2_F2_BOVINE    ------------------------------------------------------------------------------------
DENND1A.V2_R_BOVINE     ------------------------------------------------------------------------------------

DENND1A.V2_HUMAN        ------------------------------------------------------------------------------------
DENND1A_BOVINE          GGCAGAAGTGACAGGAGCCGTGGGACACCAGGGGCCTTGAAGCTCGCCCACCCGCACAGCAGGCTCTGGAGCCTAGGCCAGGAT
DENND1A.V2_F1_BOVINE    ------------------------------------------------------------------------------------
DENND1A.V2_F2_BOVINE    ------------------------------------------------------------------------------------
DENND1A.V2_R_BOVINE     ------------------------------------------------------------------------------------

DENND1A.V2_HUMAN        ------------------------------------------------------------------------------------
DENND1A_BOVINE          GACATGGCCATCCCCAGCAAGCCGCCCGCCGCCTGCCCCGAGAAGCCCTCAGCCCTGCTCGGGAACTCCCTGGCCTCACCCTGC
DENND1A.V2_F1_BOVINE    ------------------------------------------------------------------------------------
DENND1A.V2_F2_BOVINE    ------------------------------------------------------------------------------------
DENND1A.V2_R_BOVINE     ------------------------------------------------------------------------------------

DENND1A.V2_HUMAN        ------------------------------------------------------------------------------------
DENND1A_BOVINE          AGGCCCCAGAACCAGAATGGCCTCCTGAACCCCAGCGACAAGGAGGAGGTGCCCACACCCACCCTGGGCAGCATCACTATCCCC
DENND1A.V2_F1_BOVINE    ------------------------------------------------------------------------------------
DENND1A.V2_F2_BOVINE    ------------------------------------------------------------------------------------
DENND1A.V2_R_BOVINE     ------------------------------------------------------------------------------------
```

Exon 21

Exon 22 bovine

```
DENND1A.V2_HUMAN        ------------------------------------------------------------------------------------
DENND1A_BOVINE          CGGCCCCAGGGCAGGAAGACCCCAGAGCTGGGCATCGTGCCCCCGCCTCCTACTGCCCGCCCAGCCAAGCTCCAGGCCCCCGGC
DENND1A.V2_F1_BOVINE    ------------------------------------------------------------------------------------
DENND1A.V2_F2_BOVINE    ------------------------------------------------------------------------------------
DENND1A.V2_R_BOVINE     ------------------------------------------------------------------------------------

DENND1A.V2_HUMAN        ------------------------------------------------------------------------------------
DENND1A_BOVINE          ATCGCCCTTGGCGACTTCTTGGAACAACTGCCGGCTGAGCGGGAAAGGCGGGCTGCCCTCAGCTCAACCCCATTCCCTGGGCTC
DENND1A.V2_F1_BOVINE    ------------------------------------------------------------------------------------
DENND1A.V2_F2_BOVINE    ------------------------------------------------------------------------------------
DENND1A.V2_R_BOVINE     ------------------------------------------------------------------------------------

DENND1A.V2_HUMAN        ------------------------------------------------------------------------------------
DENND1A_BOVINE          CTCTCCAGTGCTGCACCCCAAGACCCCACTGAACTGCTCCAGCCACTCAGCCTGGCCCCAGGGGCTGCCGGCAGCAGCAGTGAT
DENND1A.V2_F1_BOVINE    ------------------------------------------------------------------------------------
DENND1A.V2_F2_BOVINE    ------------------------------------------------------------------------------------
DENND1A.V2_R_BOVINE     ------------------------------------------------------------------------------------

DENND1A.V2_HUMAN        ------------------------------------------------------------------------------------
DENND1A_BOVINE          GCCCTGCTGGCCCTGCTGGATCCACTTAACACAACCTGGTCGGGCAGCTCCCTTCCACCAGGCCCTACAGCCCCAAATGTAGCC
DENND1A.V2_F1_BOVINE    ------------------------------------------------------------------------------------
DENND1A.V2_F2_BOVINE    ------------------------------------------------------------------------------------
DENND1A.V2_R_BOVINE     ------------------------------------------------------------------------------------

DENND1A.V2_HUMAN        ------------------------------------------------------------------------------------
DENND1A_BOVINE          ACCCCATTTACTCCCCAATTTAGTTTTCCCCCCATGGGGACCCCCACCCCATTTCCACAGCCATCACTCAACCCCTTTGTCCCA
DENND1A.V2_F1_BOVINE    ------------------------------------------------------------------------------------
DENND1A.V2_F2_BOVINE    ------------------------------------------------------------------------------------
DENND1A.V2_R_BOVINE     ------------------------------------------------------------------------------------

DENND1A.V2_HUMAN        ------------------------------------------------------------------------------------
DENND1A_BOVINE          CCTCTGCCAGCGACACTGCCCACCATGCCCCTGGTCTCTGCACCAGCTGGGCCTTTTGGGGCCCCTCCTGCTTCCCTGGGGCCA
DENND1A.V2_F1_BOVINE    ------------------------------------------------------------------------------------
DENND1A.V2_F2_BOVINE    ------------------------------------------------------------------------------------
DENND1A.V2_R_BOVINE     ------------------------------------------------------------------------------------

DENND1A.V2_HUMAN        ------------------------------------------------------------------------------------
DENND1A_BOVINE          GCCTTTGCCCCCAGCCTCCTGCTGTCCAATTCTGGCTTCTGTGCCCCACATCGATCTCAGCCCAACCTGTCTGCCCTCTCCATG
DENND1A.V2_F1_BOVINE    ------------------------------------------------------------------------------------
DENND1A.V2_F2_BOVINE    ------------------------------------------------------------------------------------
DENND1A.V2_R_BOVINE     ------------------------------------------------------------------------------------

DENND1A.V2_HUMAN        ------------------------------------------------------------------------------------
DENND1A_BOVINE          CCCAACCTCTTTGGCCAAGTGCCCATGGGTACCCACTCCCTGCAGCCTCTGGGTCCCCCAGCAGTCGCCCCCTCAAGGATCCGA
DENND1A.V2_F1_BOVINE    ------------------------------------------------------------------------------------
DENND1A.V2_F2_BOVINE    ------------------------------------------------------------------------------------
DENND1A.V2_R_BOVINE     ------------------------------------------------------------------------------------

DENND1A.V2_HUMAN        ------------------------------------------------------------------------------------
DENND1A_BOVINE          ACATTGCCCCTGGCCCGCTCAAGTGCCAGGGCCGCCGAGGCCAAGCAAGGGCTGGCCCTGAGGCCTGGAGAACCCCCACTCCTC
DENND1A.V2_F1_BOVINE    ------------------------------------------------------------------------------------
DENND1A.V2_F2_BOVINE    ------------------------------------------------------------------------------------
DENND1A.V2_R_BOVINE     ------------------------------------------------------------------------------------

DENND1A.V2_HUMAN        --------------------------------------------------------------AGACTTTTTTGAAGATTTGTGG
DENND1A_BOVINE          CCAGCCAGGCCCCCCCAGGGCCTGGAGCCAGCACTGCAGCCCTCTGCTCCACGAGAGGCCAGAGACCCCTTTGAGGATTTGTTA
DENND1A.V2_F1_BOVINE    ------------------------------------------------------------------------------------
DENND1A.V2_F2_BOVINE    ------------------------------------------------------------------------------------
DENND1A.V2_R_BOVINE     ------------------------------------------------------------------------------------

DENND1A.V2_HUMAN        GGGAAA------------------------------------------------------------------------------
DENND1A_BOVINE          CGGAAAACCAAGCAAGATGTGAGCTCGGCCCCAGCCCCCGGCTCCGTGGAGCAGCTCAGGAAGCAATGGGAGACCTTCGAGTGA
DENND1A.V2_F1_BOVINE    ------------------------------------------------------------------------------------
DENND1A.V2_F2_BOVINE    ------------------------------------------------------------------------------------
DENND1A.V2_R_BOVINE     ------------------------------------------------------------------------------------

DENND1A.V2_HUMAN        ------------------------------------------------------------------------------------
DENND1A_BOVINE          CTGCTGTTTCTACCCAGGTTCTACTGGTGGGAAGGGATGGGAACCCTCTCTGCCGCCCCTCCTCCTTTCCACACTGCCCATCTC
DENND1A.V2_F1_BOVINE    ------------------------------------------------------------------------------------
DENND1A.V2_F2_BOVINE    ------------------------------------------------------------------------------------
DENND1A.V2_R_BOVINE     ------------------------------------------------------------------------------------
```

Exon 21 (human)/22 (bovine)

```
DENND1A.V2_HUMAN        -------------------------------------------------------CATTCCCTTTACTTTTCAGGTAACATTAC
DENND1A_BOVINE          TGGAGAATGGCGCCAGTTCCAGCCTGGGAATCGACCCAGCTCCTGGGTGCCTGTCCTGTCCCGACCCTTCCCCTGCCCCCCTAG
DENND1A.V2_F1_BOVINE    ------------------------------------------------------------------------------------
DENND1A.V2_F2_BOVINE    ------------------------------------------------------------------------------------
DENND1A.V2_R_BOVINE     ------------------------------------------------------------------------------------

DENND1A.V2_HUMAN        TTGGTT------------------------------------------------------------------------------
DENND1A_BOVINE          TTGGGTTTTGCACTAAAGAGGTCAGCTGGGCCAGCGATTGCCCCAGGCCACATCTTACCCACCTTCCCTCTGGAAACTGCCCAC
DENND1A.V2_F1_BOVINE    ------------------------------------------------------------------------------------
DENND1A.V2_F2_BOVINE    ------------------------------------------------------------------------------------
DENND1A.V2_R_BOVINE     ------------------------------------------------------------------------------------

DENND1A.V2_HUMAN        ------------------------------------------------------------------------------------
DENND1A_BOVINE          CAGGAGCCCCGCGCGTCCTCAGGATGTCTCCTCCTGAGCCCCGTTCTCTTGCCCCAGCTGCAGCCCAGCCCGCACACCCCACCT
DENND1A.V2_F1_BOVINE    ------------------------------------------------------------------------------------
DENND1A.V2_F2_BOVINE    ------------------------------------------------------------------------------------
DENND1A.V2_R_BOVINE     ------------------------------------------------------------------------------------

DENND1A.V2_HUMAN        ------------------------------------------------------------------------------------
DENND1A_BOVINE          TGATGGGCACGAGTGTTGGCGGTACACCAGCTCGTCTGCACGAGAGGCACGTGGTTTGGAGTTTGAAGTAAGGAACCCCCCTCC
DENND1A.V2_F1_BOVINE    ------------------------------------------------------------------------------------
DENND1A.V2_F2_BOVINE    ------------------------------------------------------------------------------------
DENND1A.V2_R_BOVINE     ------------------------------------------------------------------------------------

DENND1A.V2_HUMAN        -------------------------------------------------------------------AATATTCAGTTACTAAA
DENND1A_BOVINE          CCCACCCCTCCCCAGCTGGAGGATCTACCTGGCGAGGCATTTCCAAACCCTGTCTAGCAATATGCACACTCTTTCTTTACTGAG
DENND1A.V2_F1_BOVINE    ------------------------------------------------------------------------------------
DENND1A.V2_F2_BOVINE    ------------------------------------------------------------------------------------
DENND1A.V2_R_BOVINE     ------------------------------------------------------------------------------------

DENND1A.V2_HUMAN        TCTCTTCAAGAA------------------------------------------------------------------------
DENND1A_BOVINE          TCTTCACCCCAACCCCTCCACCTCCGTTTTTCTGACTTGGTTGGGATCTGGGGGC-----------------------------
DENND1A.V2_F1_BOVINE    ------------------------------------------------------------------------------------
DENND1A.V2_F2_BOVINE    ------------------------------------------------------------------------------------
```

Exon 21 (human)/22 (bovine)
