# Supplementary material for: Could perturbed fetal development of the ovary contribute to the development of polycystic ovary syndrome in later life?
Source: PLoS One. 2020 Feb 20;15(2):e0229351. doi: 10.1371/journal.pone.0229351 (PMC7032716; doi:10.1371/journal.pone.0229351)

Fig S5. **An adjacent matrix network graph of bovine gene expression using correlation coefficients from Tables 2 generated with R-program.** The closeness of the genes and the thickness of the interconnecting lines indicate the strength of the correlations between genes. Red and blue lines represent positive and negative correlation, respectively. Age is gestational age.

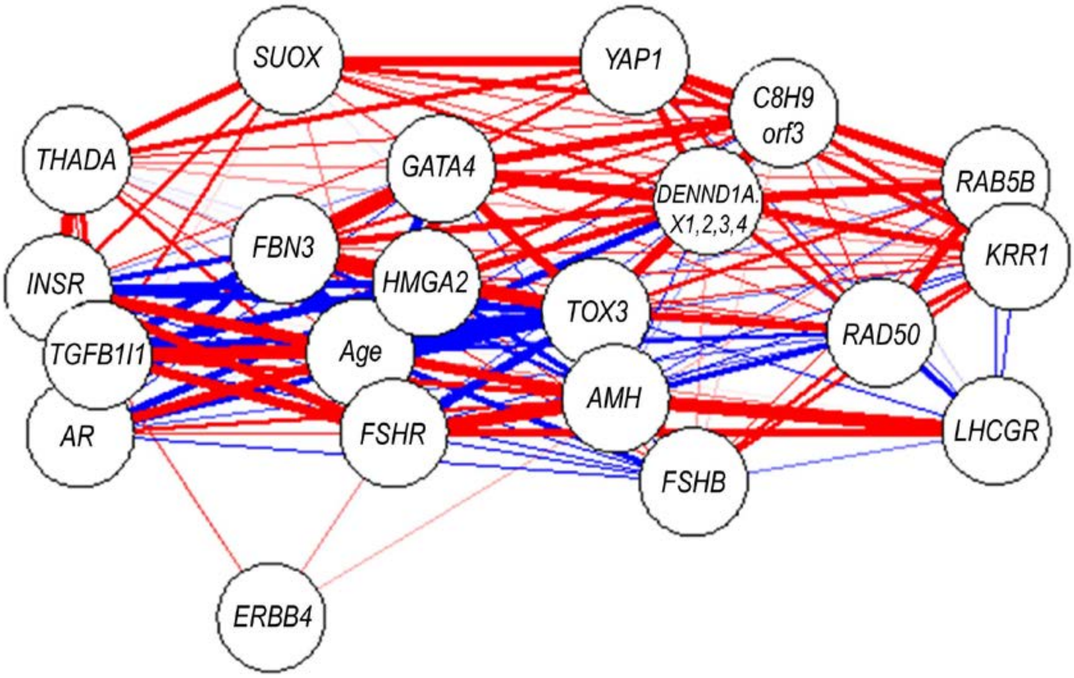

Supplement: S5 Fig — (PDF) [file pone.0229351.s005.pdf]
