# Supplementary material for: Could perturbed fetal development of the ovary contribute to the development of polycystic ovary syndrome in later life?
Source: PLoS One. 2020 Feb 20;15(2):e0229351. doi: 10.1371/journal.pone.0229351 (PMC7032716; doi:10.1371/journal.pone.0229351)

Fig S6. **Differential mRNA expression levels of PCOS candidate genes which are highly expressed during early gestation in the cortex and medulla of bovine fetal ovaries.** Samples were grouped into early (n = 5, 10 – 17 weeks of gestation) and later (n= 5, 36 – 39 weeks of gestation) time of gestation. Data are presented as mean ± s.e.m. (normalised to *PPIA* and *RPL32*). Black and grey bars represent cortex and medulla, respectively. Unpaired t-test were applied to analyse the data. **P* < 0.05.

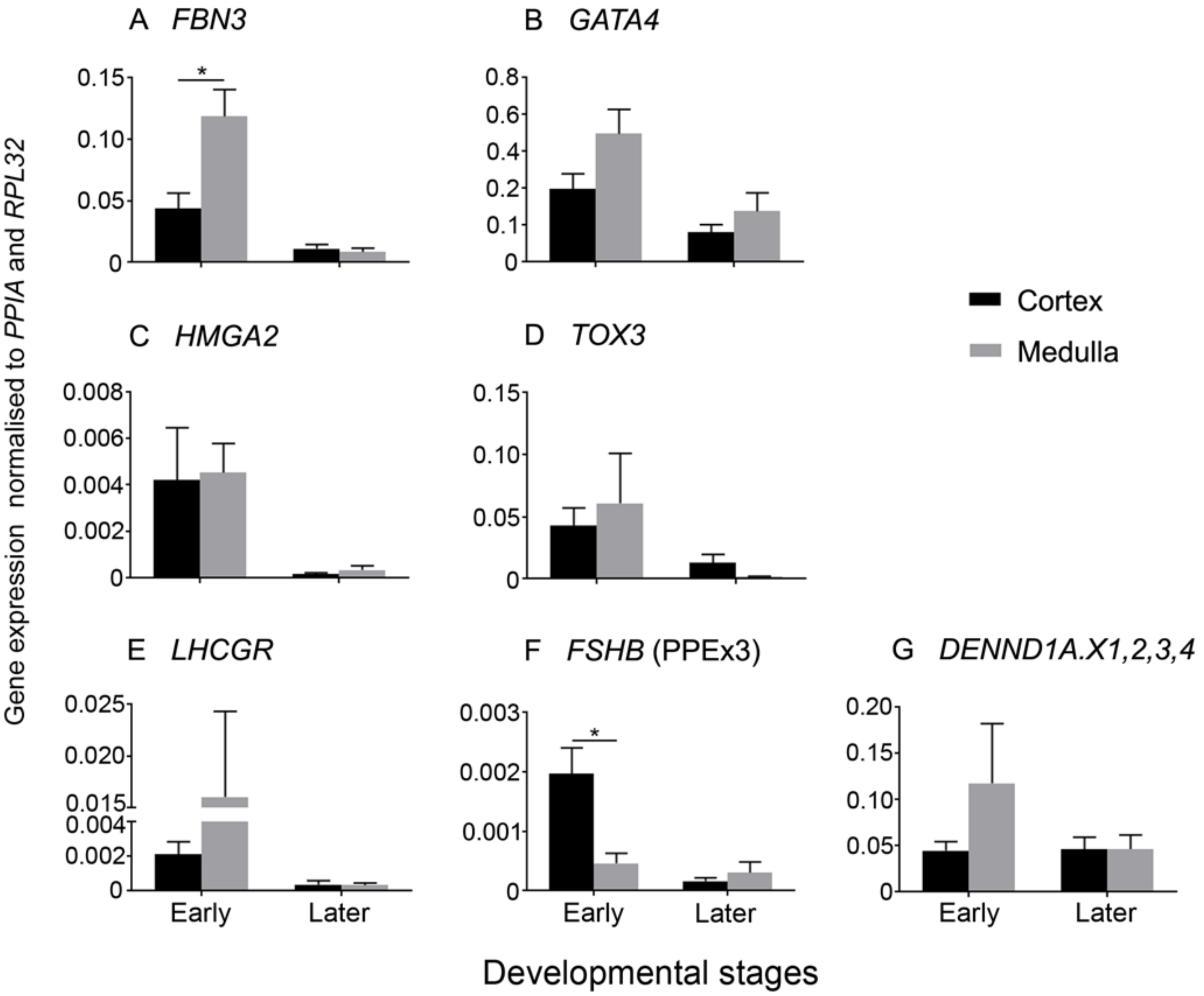

Supplement: S6 Fig — (PDF) [file pone.0229351.s006.pdf]
