# Supplementary material for: Could perturbed fetal development of the ovary contribute to the development of polycystic ovary syndrome in later life?
Source: PLoS One. 2020 Feb 20;15(2):e0229351. doi: 10.1371/journal.pone.0229351 (PMC7032716; doi:10.1371/journal.pone.0229351)

Fig S8. Differential mRNA expression levels of PCOS candidate genes which are steadily expressed throughout gestation in the cortex and medulla of bovine fetal ovaries. Samples were grouped into early (n = 5, 10 – 17 weeks of gestation) and late (n= 5, 36 – 39 weeks of gestation) stages of gestation. Data are presented as mean ± s.e.m. (normalised to *PPIA* and *RPL32*). Black and grey bars represent cortex and medulla, respectively. Unpaired t-test were applied to analyse the data. *$P < 0.05$.

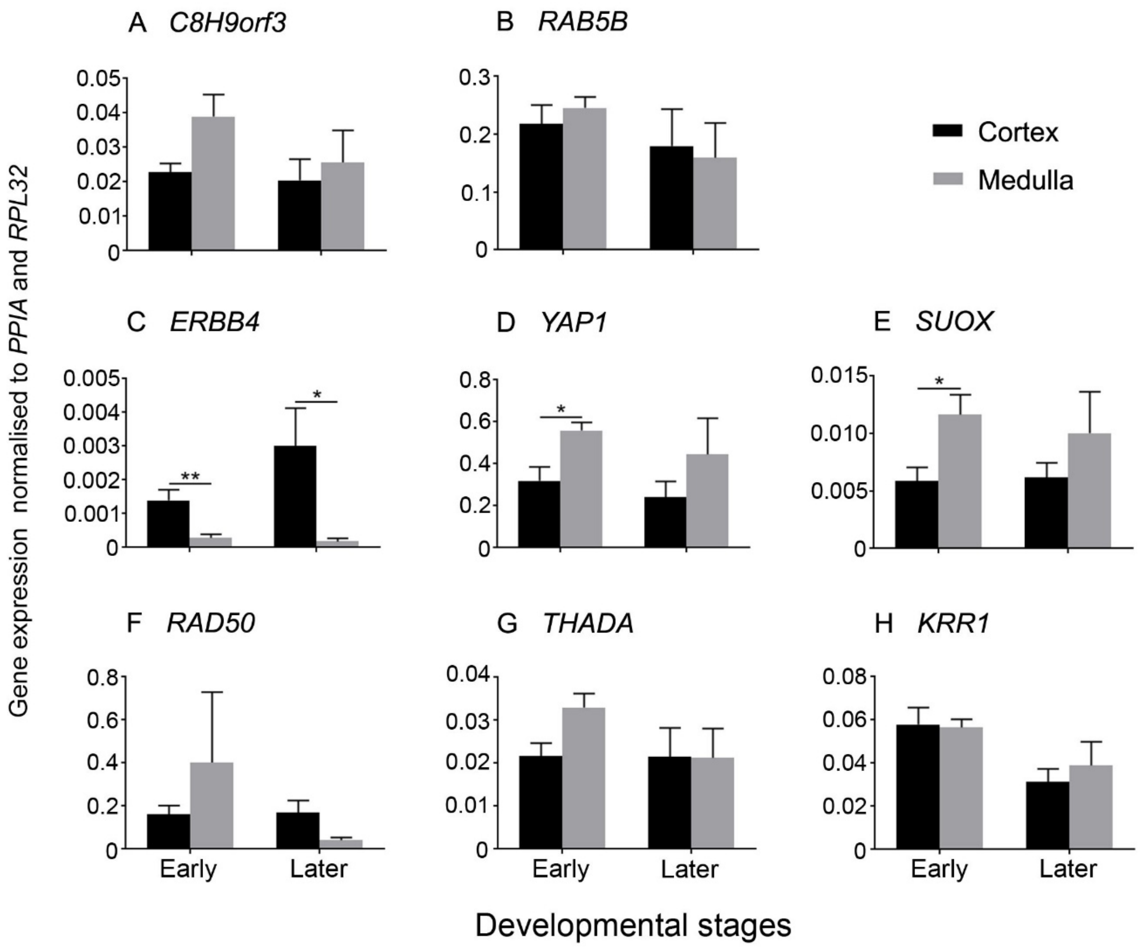

Supplement: S8 Fig — (PDF) [file pone.0229351.s008.pdf]
