# Supplementary material for: Could perturbed fetal development of the ovary contribute to the development of polycystic ovary syndrome in later life?
Source: PLoS One. 2020 Feb 20;15(2):e0229351. doi: 10.1371/journal.pone.0229351 (PMC7032716; doi:10.1371/journal.pone.0229351)

Fig S12. **Expression of PCOS candidate genes which are highly expressed steadily during gestation in bovine fetal fibroblasts from less than 150 days of gestation cultured in the presence of treatments grouped as indicated (A to F) for 18 h.** Data are represented as mean ± s.e.m. of fold change in expression relative to the untreated control (n = 4 ovaries, each from 13, 14, 17 and 19 weeks of gestation). One-way ANOVA with Dunnet's *post hoc* test were used to analyse the data.

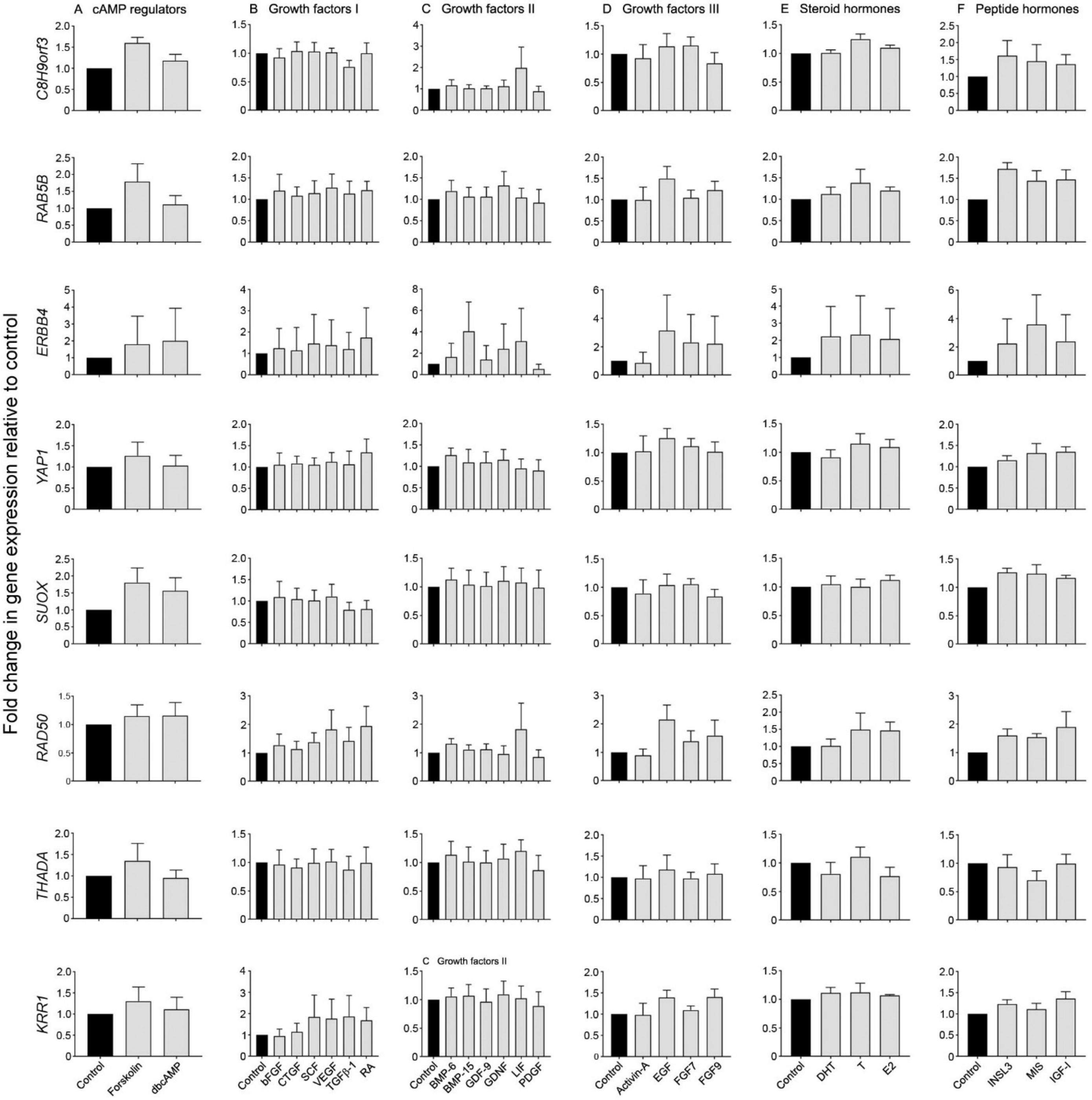

Supplement: S12 Fig — Expression of PCOS candidate genes which are highly expressed steadily during gestation in bovine fetal fibroblasts from less than 150 days of gestation cultured in the presence of treatments grouped as indicated (A to F) for 18 h. (PDF) [file pone.0229351.s012.pdf]
