# Supplementary material for: Could perturbed fetal development of the ovary contribute to the development of polycystic ovary syndrome in later life?
Source: PLoS One. 2020 Feb 20;15(2):e0229351. doi: 10.1371/journal.pone.0229351 (PMC7032716; doi:10.1371/journal.pone.0229351)
Supplement: S1 Table — (PDF) [file pone.0229351.s013.pdf]

Table S1. List of genes and primers used for qRT-PCR *.

Gene name	Gene Symbol	Species	Primers (5'→3') (F = forward, R = reverse)	Genebank Accession Number	Size (bp)
Ribosomal protein L32	*RPL32*	Bovine	F : GCCATCAGAATCACCAATCC<br>R : AAATGTGCACACGAGCTGTC	NM_001034783.2	73
Ribosomal protein L32	*RPL32*	Human	F:CATCTCCTTCTCGGCATCA<br>R:AACCCTGTTGTCAATGCCT	NM_000994	153
Ribosomal protein L19	*RPL19*	Bovine	F: GATCCGGAAGCTGATCAAAG<br>R: TACCCATATGCCTGCCTTTC	NM_001040516.1	113
Peptidylprolyl isomerase A (cyclophilin A)	*PPIA*	Bovine	F : CTGGCATCTTGTCCATGGCAAA<br>R : CCACAGTCAGCAATGGTGATCTTC	NM_178320.2	202
Beta-2-microglobulin	*B2M*	Human	F:ACTGAATTCACCCCCACTGA<br>R:CCTCCATGATGCTGCTTACA	NM_004048	114
Fibrillin 3	*FBN3*	Bovine	F : GCCACAGCCTGCCTAGATGT<br>R : CTGCCCTCAGTGTTTTTGCA	XM_001254849.2	82
Fibrillin 3	*FBN3*	Human	F : TGTCGTACCCATCTGTAGGC<br>R : GCCCCCATTCATACAGCTCA	NM_032447	142
High Mobility Group AT-Hook 2	*HMGA2*	Bovine	F : TTATCCGCCCACGATTAGAG<br>R : TTGAGTGTGTGTGTGCTTGG	XM_002704288	72
High Mobility Group AT-Hook 2	*HMGA2*	Human	F : TCAGAAGAGAGGACGCGG<br>R : TTGAGCTGCTTTAGAGGGAC	NM_003483	124
TOX High Mobility Group Box Family Member 3	*TOX3*	Bovine	F : TATGGCTGAGGCAAACAACG<br>R : TTCAAACTCCTCATCCCCAAGG	XM_015467340	79
TOX High Mobility Group Box Family Member 3	*TOX3*	Human	F : AGCAAATCCAGCAGCAGATG<br>R : GAGGAGAAGGCTGAGACTGG	NM_001146188	180
GATA binding protein 4	*GATA4*	Bovine	F : CAGGAGGCAAAAATGCTAGG<br>R : ATCACCCGTCGTCTTTCTTC	NM_001192877.1	82
GATA binding protein 4	*GATA4*	Human	F : CATCAAGACGGAGCCTGGCC<br>R : TGACTGTCGGCCAAGACCAG	NM_002052	219
DENN Domain Containing 1A	*DENND1A.X1,2,3,4*	Bovine	F : TGTCGTGATCCTGAATGTGG<br>R : ACATCATTTGGGAGGCTCTG	XM_005213126	71
Predicted DENN Domain Containing 1A Variant 2	*DENND1A.V2*	Bovine	F : ACCGAAGAGCAACATCACAG<br>R : TGACGCAGCAATCTCCTATC	NM_001193014	306
DENN Domain Containing 1A Variant 1-7	*DENND1A.V1-7*	Human	F : GTCCATCTCAGCGTGCATTC<br>R : CGGCGTTCGTACAGCATAC	NM_020946	149

DENN Domain Containing 1A Variant 1, 3, 4	*DENND1A. V1,3,4*	Human	F : CAGCCAGGGACCTTTGACTA<br>R : CAGAGCTTGTTGTACGGGTG	NM_020946	104
Anti-Mullerian hormone	*AMH*	Bovine	F : ACACCGGCAAGCTCCTCAT<br>R : TCTCGTCCGCTACTCCAAGT	NM_178318.4	202
Follicle stimulating hormone receptor (FSHR)	*FSHR*	Bovine	F : GACCCTGATGCCTTCCAGA<br>R : TGGCAAGTGCTTAATACCTGTGTT	NM_174061.1	74
Follicle stimulating hormone receptor (FSHR)	*FSHR*	Human	F: GCTGCCTACTCTGGAAAAGC<br>R: ATCTCTGACCCCTAGCCTGA	NM_000145	173
Insulin receptor	*INSR*	Bovine	F : AGGAGCTGGAGGAGTCCTCGTTCA<br>R : CATTCCCCACGTCACCAAGGGCTC	XM_0154640	147 bp from X1,2 and 111 bp from X3,4
Androgen Receptor	*AR*	Bovine	F : TGCCCCTGACCTGGTTTTC<br>R : TCGGACACACTGGCTGTACATC	NM_001244127.1	67
Transforming growth factor beta 1 induced transcript 1	*TFGB1I1*	Bovine	F : TCCCCTGTTCTCCCAAAGC<br>R : GCCCTGAGGCTGGAAGATG	NM_001035313.1	109
Thyroid adenoma associated	*THADA*	Bovine	F : TGTGGTTAGGAGGCTTTTGG<br>R : ACAGTGAGCTGGTGCATTTG	XM_015465336	117
Erb-B2 Receptor Tyrosine Kinase 4	*ERBB4*	Bovine	F : CCTGGAAATAACCAGCATCG<br>R : CTTTGTCCCACGGATAATGC	XM_015462361	142
RAD50 Double Strand Break Repair Protein	*RAD50*	Bovine	F : GCTTTGAGCTTGGACCATTC<br>R : AACAGTTGGCTGGCAGTTTC	NM_001206868	100
Chromosome 8 open reading frame, human C9orf3	*C8H9orf3*	Bovine	F : ACAGGGGATGAAAGTTGTGG<br>R : AAATCTCAGAAGGGGCTTCC	NM_001206980	111
Yes Associated Protein 1	*YAP1*	Bovine	F : GATGGTGGGACTCAAAATCC<br>R : TGAGCTATTGGTCGTCATGG	XM_015474584	75
RAB5B, Member RAS Oncogene Family	*RAB5B*	Bovine	F : AAGCGCATGGTGGAGTATG<br>R : TTCTGGGGTTCACTCTTTGG	XM_005206651	146
Predicted SUMO Pseudogene 1	*SUMO1P1*	Bovine	F : CAGGGTTATTGGACAGGATAGC<br>R : TGAGGGAATTCACTGGAACG	NM_001035458.1	116
Luteinising hormone/chorionic gonadotrophin receptor	*LHCGR*	Bovine	F : GCCACTGCTGTGCTTTTAGAAA<br>R : CCAGCCACTCAGTTCACTCTCA	NM_174381.1	158 bp from X1,2,4 and 5 and 77 bp from X3
Luteinising hormone/chorionic gonadotrophin receptor	*LHCGR*	Human	F : TCAATGTGGTGGCCTTCTTCATA<br>R : TTGGCACAAGAATTGATGGGATA	NM_000233	256
Sulphite Oxidase	*SUOX*	Bovine	F : TGGTGATAACTCCAGCACCAG	NM_001034366	79

			R : ATCATGACAGGCCAACACTG		
KRR1, Small Subunit Processome Component Homolog	*KRR1*	Bovine	F : CCGAGATGAATCTGAACTCCTC<br>R : TCTGGGATTGTCCTCTTTGG	NM_001037819	76
Follicle Stimulating Hormone Beta Subunit.	*FSHB*	Bovine	Primer pair 1 in exon 3.<br>F: GTCACCACTCAGACCTGTATTC<br>R: GGGATTGCCTGAGAGGATTT	NM_174060.1	120
Follicle Stimulating Hormone Beta Subunit.	*FSHB*	Bovine	Primer pair 2 in exons 2/3.<br>F: TGACCAACATCACCATCACG<br>R: TCTGGATATTGGGCCTTGCTG	NM_174060.1	132
Follicle Stimulating Hormone Beta Subunit	*FSHB*	Human	F : TGAGCTGACCAACATCACCA<br>R : TGGCTGGGTCCTTATACACC	NM_000510	122
Cytokeratin 19	*KRT19*	Bovine	F : AAGCTTTGCGCATGAGTGTG<br>R : TCAATCTGCATCTCCAGGTCAG	NM_001015600.3	97
DEAD (Asp-Glu-Ala-Asp) box polypeptide 4	*VASA*	Bovine	F : ATGAAGCTGATCGCATGCTG<br>R : TGACGCTGTTCCTTTGATGG	NM_001007819.1	91
POU class 5 homeobox 1	*OCT4*	Bovine	F : AGGCTTTGCAGCTCAGTTTC<br>R : TTGTTGTCAGCTTCCTCCAC	NM_174580.2	79

* To check correct amplification of bovine genes, PCR products from a young and old fetuses, and additionally the anterior pituitary for *FSHB* were subjected to electrophoresis on agar gels and then stained with ethidium bromide. Purified PCR products were then sequenced by Sanger method at the Australian Genome Facility using the forward primers. The following all proved to amplify the correct sequences: *FBN3*, *GATA4*, *HMGA2*, *TOX3*, *LHCGR* (158 bp from X1,2,4 and 5 and 77 bp from X3), *FSHB* (primer pair, Exon 3), *FSHB* (primer pair, Exon 2/3), *DENND1A.X1,2,3,4*, *INSR* (147 bp from X1,2 and 111 bp from X3,4), *FSHR*, *AMH*, *AR*, *TGFB1I1*, *C8H9orf3*, *RAB5B*, *ERBB4*, *YAP1*, *SUOX*, *RAD50*, *THADA* and *KRR1*.
