# Supplementary material for: Could perturbed fetal development of the ovary contribute to the development of polycystic ovary syndrome in later life?
Source: PLoS One. 2020 Feb 20;15(2):e0229351. doi: 10.1371/journal.pone.0229351 (PMC7032716; doi:10.1371/journal.pone.0229351)
Supplement: S2 Table — (PDF) [file pone.0229351.s014.pdf]

Table S2. Treatments used for bovine fetal fibroblasts.

Category	Treatments	Concentration	Distribution
Stimulators	Forskolin	4.1 μg/mL	Sigma-Aldrich
	Dibutyryladenosine cyclic monophosphate	1mM	Sigma-Aldrich
Growth factors	Basic fibroblast growth factor	100 ng/mL	Roche Australia Pty Ltd, Thebarton, SA, Australia
	Connective tissue growth factor	25 ng/mL	Invitrogen/Life Technologies
	Stem cell factor	100 ng/mL	R&D Systems
	Vascular endothelial growth factor	10 ng/mL	R&D Systems
	Transforming growth factor β1	10 ng/mL	R&D Systems
	Bone morphogenetic protein 6	100 ng/mL	R&D Systems
	Bone morphogenetic protein 15	100 ng/mL	R&D Systems
	Glial-derived factor 9	100 ng/mL	R&D Systems
	Glial-cell derived neurotrophic factor	100 ng/mL	R&D Systems
	Leukemia inhibitory factor	10^3 U/mL	Sigma-Aldrich
	Platelet-derived growth factor	10 ng/mL	R&D Systems
	Activin A	100 ng/mL	R&D Systems
	Epidermal growth factor	10 ng/mL	Boehringer Ingelheim Pty Ltd, North Ryde, NSW, Australia
	Fibroblast growth factor 7	10 ng/mL	R&D Systems
	Fibroblast growth factor 9	30 ng/mL	R&D Systems
	Retinoic acid	3 μg/mL	Sigma-Aldrich
Hormones	Dihydroxytestosterone	100 ng/mL	Sigma-Aldrich
	Testosterone	100 ng/mL	Sigma-Aldrich
	Estradiol	100 ng/mL	Sigma-Aldrich
	Insulin-like protein 3	100 ng/mL	From Dr Ross Bathgate – University of Melbourne, Australia
	Mullerian-inhibiting substance	10 ng/mL	Biogen Idec Australia Pty Ltd, North Ryde, NSW, Australia
	Insulin-like growth factor 1	30 ng/mL	GroPep Bioreagents Pty Ltd, Thebarton, SA, Australia
