# Supplementary material for: Could perturbed fetal development of the ovary contribute to the development of polycystic ovary syndrome in later life?
Source: PLoS One. 2020 Feb 20;15(2):e0229351. doi: 10.1371/journal.pone.0229351 (PMC7032716; doi:10.1371/journal.pone.0229351)
Supplement: S3 Table — (PDF) [file pone.0229351.s015.pdf]

Table S3. Pearson correlation coefficients (r) of mRNA expression levels of PCOS-candidate genes and gestational age in less than 150 day bovine fetal ovaries (n = 27).

	**Age**	***FBN3***	***GATA4***	***HMGA2***	***TOX3***	***LHCGR***	***FSHB***	***DENND1A.X12,3,4***	***C8H9orf3***	***RAB5B***	***ERBB4***	***YAP1***	***SUOX***	***RAD50***	***THADA***	***KRR1***	***INSR***	***FSHR***	***AMH***	***AR***
FBN3	-0.675[a]																			
GATA4	-0.650[a]	0.920[d]																		
HMGA2	-0.651[a]	0.850[c]	0.802[b]																	
TOX3	0.223	0.119	0.219	0.021																
LHCGR	-0.922[d]	0.609[a]	0.719[a]	0.651[a]	-0.126															
FSHB (PPEx3)	-0.530	0.515	0.679[a]	0.688[a]	0.059	0.730[a]														
DENND1A. X1,2,3,4	-0.165	0.641[a]	0.708[a]	0.591	0.502	0.295	0.518													
C8H9orf3	-0.163	0.723[a]	0.572	0.536	0.168	0.006	0.128	0.569												
RAB5B	0.592	-0.325	-0.216	-0.231	0.375	-0.504	-0.314	0.088	-0.095											
ERBB4	0.426	-0.266	-0.237	-0.049	0.287	-0.412	-0.045	-0.047	-0.038	0.758[b]										
YAP1	-0.064	0.617[a]	0.671[a]	0.632[a]	0.553	0.159	0.448	0.607[a]	0.501	0.324	0.335									
SUOX	-0.253	0.821[b]	0.819[b]	0.761[b]	0.491	0.300	0.424	0.817[b]	0.720[a]	0.048	-0.027	0.841[b]								
RAD50	0.237	0.043	0.0510	0.118	0.473	-0.247	0.014	0.048	0.092	0.679[a]	0.872[c]	0.628[a]	0.234							
THADA	-0.189	0.139	0.104	0.436	0.242	0.222	0.420	0.172	-0.158	0.225	0.600	0.389	0.159	0.629[a]						
KRR1	0.392	-0.081	-0.155	0.146	0.460	-0.355	0.151	0.180	0.017	0.385	0.688[a]	0.489	0.192	0.695[a]	0.740[b]					
INSR	0.764[b]	-0.114	-0.165	-0.245	0.387	-0.773[b]	-0.323	0.208	0.469	0.393	0.289	0.333	0.256	0.276	-0.287	0.385				
FSHR	0.276	0.418	0.381	0.155	0.570	-0.291	-0.183	0.565	0.661[a]	0.316	-0.030	0.625a	0.708a	0.217	-0.281	0.119	0.707[a]			
AMH	0.719[a]	-0.615[a]	-0.458	-0.453	0.327	-0.470	-0.050	-0.014	-0.435	0.237	0.100	-0.034	-0.188	-0.047	-0.065	0.385	0.434	0.064		
AR	0.939[d]	-0.584	-0.561	-0.520	0.133	-0.811[b]	-0.319	-0.051	-0.139	0.377	0.266	-0.054	-0.186	0.064	-0.178	0.417	0.742[b]	0.224	0.811[b]	
TGFβ1I1	0.951[d]	-0.456	-0.411	-0.488	0.371	-0.848[c]	-0.404	0.088	0.071	0.553	0.344	0.132	0.019	0.216	-0.261	0.372	0.887[c]	0.504	0.707[a]	0.921[d]

[a] $P < 0.05$, [b] $P < 0.01$, [c] $P < 0.001$, [d] $P < 0.0001$
