# Supplementary material for: Could perturbed fetal development of the ovary contribute to the development of polycystic ovary syndrome in later life?
Source: PLoS One. 2020 Feb 20;15(2):e0229351. doi: 10.1371/journal.pone.0229351 (PMC7032716; doi:10.1371/journal.pone.0229351)
Supplement: S4 Table — (PDF) [file pone.0229351.s016.pdf]

Table S4. Normalised gene expression in cultured bovine fetal fibroblast (*in vitro*, n = 4) and whole bovine fetal ovaries (*in vivo*, n = 5) at 13-28 weeks of gestation.

Genes	*In vitro*	*In vivo*
FBN3	0.0009 ± 0.0002	0.008 ± 0.002
GATA4	0.136 ± 0.0212	0.004 ± 0.001
HMGA2	0.0005 ± 0.0001	0.003 ± 0.0004
TOX3	0.005 ± 0.003	0.342 ± 0.037
LHCGR	0.0005 ± 0.0003	0.040 ± 0.003
FSHB	Not detected	0.004 ± 0.001
DENND1A.X1,2,3,4	0.022 ± 0.005	0.151 ± 0.026
INSR	0.0166 ± 0.004	0.044 ± 0.002
FSHR	Not detected	0.536 ± 0.046
AMH	0.0004 ± 0.0001	0.004 ± 0.0008
AR	0.012 ± 0.004	0.315 ± 0.024
TGFB1I1	0.231 ± 0.050	0.010 ± 0.0008
C8H9orf3	0.008 ± 0.001	0.061 ± 0.004
RAB5B	0.112 ± 0.024	0.052 ± 0.008
ERBB4	0.002 ± 0.002	0.057 ± 0.013
YAP1	0.330 ± 0.063	0.081 ± 0.005
SUOX	0.007 ± 0.002	0.073 ± 0.011
RAD50	0.043 ± 0.008	0.127 ± 0.023
THADA	0.0276 ± 0.007	0.096 ± 0.017
KRR1	0.025 ± 0.007	0.002 ± 0.0006

Data are presented as mean ± s.e.m.
